# Supplementary material for: SRAS1.1 E3 ligase mediates DSK2A degradation to regulate autophagy and drought tolerance in Arabidopsis
Source: EMBO Rep. 2025 Aug 22;26(19):4794–819. doi: 10.1038/s44319-025-00556-9 (PMC12508185; doi:10.1038/s44319-025-00556-9)
Supplement: Supplementary file 1 — Appendix [file 44319_2025_556_MOESM1_ESM.pdf]

Appendix

Appendix Figure S1-9

Table of Contents

Appendix Figure S1. *sras1.1* mutants display enhanced drought tolerance..................2

Appendix Figure S2. Identification of proteins interacting with SRAS1.1.......................3

Appendix Figure S3. LCI assay identifies the interaction between SRAS1.1 and DSK2A. 4

Appendix Figure S4. SRAS1.1 acts upstream of DSK2A under drought stress.............5

Appendix Figure S5. SRAS1.1 and DSK2A mediate *Arabidopsis* flowering time...........6

Appendix Figure S6. Predicted structural divergence between DSK2A and DSK2B......7

Appendix Figure S7. The RING domain is required for the interaction between SRAS1.1 and DSK2A. 8

Appendix Figure S8. SRAS1.2 Interacts with DSK2B to prevent Its degradation. 9

Appendix Figure S9. Drought stress response of *SRAS1.2*-overexpressing plants.......10

Appendix Figure S1

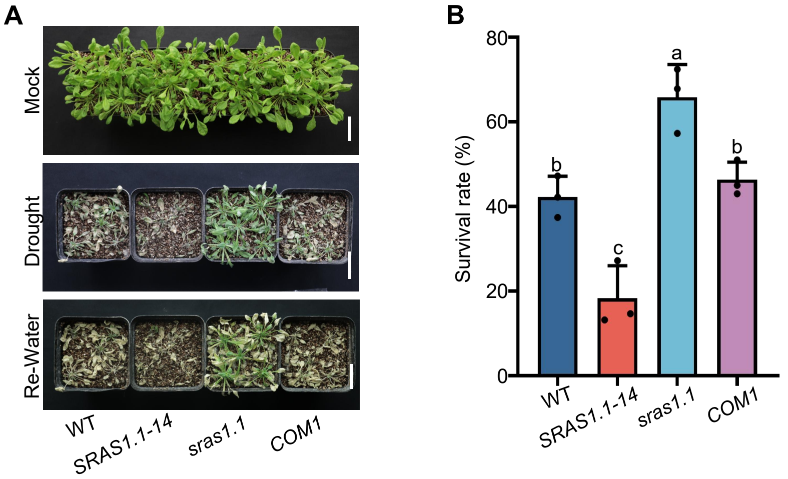

Appendix Figure S1. *sras1.1* mutants display enhanced drought tolerance.

(A) Drought tolerance assay of the wild-type, *SRAS1.1-14*, *sras1.1* mutants and *proSRAS1.1:SRAS1.1/sras1.1* complementation line (*COM1*) plants grown under normal growth conditions for 2 weeks, subjected to drought stress for 16 days and then rewatered for 5 days. Scale bars = 2 cm.
(B) Quantification of survival rates after drought and rewatering treatment. Values shown are means ± SD (n = 3 biological replicates). Statistical significance was determined by one-way ANOVA in combination with Tukey's multiple comparisons test ($P < 0.05$). P values < 0.0001 (wild-type vs *SRAS1.1-14*), <0.0001 (wild-type vs *sras1.1*), 0.9908 (wild-type vs *COM1*), <0.0001 (*SRAS1.1-14* vs *sras1.1*), 0.0007 (*SRAS1.1-14* vs *COM1*), <0.0001 (*sras1.1* vs *COM1*).

Appendix Figure S2

A

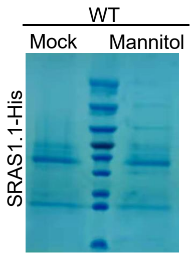

B

Serial number	Gene ID	Gene Name	Gene function
1	AT2G17200	DSK2B	putative DSK2 ubiquitin-proteasome shuttle factor
2	AT1G73310	SCPL4	putative SCPL clade-I acyltransferase
3	AT3G26650	GAPA1	photosynthetic glyceraldehyde-3-phosphate dehydrogenase
4	AT1G06770	DRIP1	DREB2A-INTERACTING PROTEIN 1

Appendix Figure S2. Identification of proteins interacting with SRAS1.1.

(A) Polyacrylamide gel electrophoresis analysis of SRAS1.1-His co-precipitated proteins under control (mock) and drought (250 mM mannitol) conditions. Gels were stained with Coomassie Brilliant Blue. (B) Immunoprecipitation followed by mass spectrometry (IP-MS) was performed to identify SRAS1.1-interacting proteins under drought stress. Sequence data for the identified proteins are available in the *Arabidopsis* UniProt database.

Appendix Figure S3

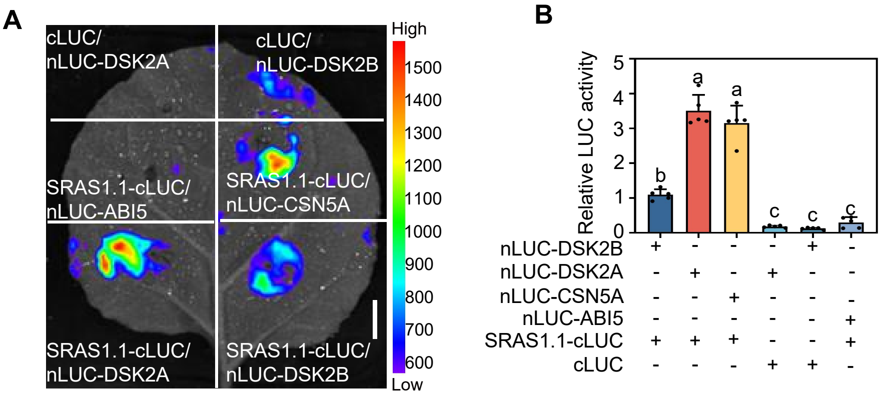

Appendix Figure S3. LCI assay identifies the interaction between SRAS1.1 and DSK2A.

(A, B) Luciferase complementation imaging assay (LCI) (A) and LUC activity measurement (B) showed that SRAS1.1 interacts with DSK2A and DSK2B. The empty vector nLUC-ABI5served as a negative control, and nLUC-CSN5A was used as a positive control. Scale bar = 1 cm. Values shown are means ± SD (n = 5 biological replicates). Statistical significance was determined by one-way ANOVA in combination with Tukey's multiple comparisons test ($P < 0.05$). *P* values < 0.0001 (nLUC-DSK2B + SRAS1.1-cLUC vs nLUC-DSK2A + SRAS1.1-cLUC), <0.0001 (nLUC-DSK2B + SRAS1.1-cLUC vs nLUC-CSN5A + SRAS1.1-cLUC), 0.0027 (nLUC-DSK2B + SRAS1.1-cLUC vs nLUC-DSK2A + cLUC), 0.0006 (nLUC-DSK2B + SRAS1.1-cLUC vs nLUC-DSK2B + cLUC), 0.0103 (nLUC-DSK2B + SRAS1.1-cLUC vs nLUC-ABI5 + SRAS1.1-cLUC), 0.4134 (nLUC-DSK2A + SRAS1.1-cLUC vs nLUC-CSN5A + SRAS1.1-cLUC), <0.0001 (nLUC-DSK2A + SRAS1.1-cLUC vs nLUC-DSK2A + cLUC), <0.0001 (nLUC-DSK2A + SRAS1.1-cLUC vs nLUC-DSK2B + cLUC), <0.0001 (nLUC-DSK2A + SRAS1.1-cLUC vs nLUC-ABI5 + SRAS1.1-cLUC), <0.0001 (nLUC-CSN5A + SRAS1.1-cLUC vs nLUC-DSK2A + cLUC), <0.0001 (nLUC-CSN5A + SRAS1.1-cLUC vs nLUC-DSK2B + cLUC), <0.0001 (nLUC-CSN5A + SRAS1.1-cLUC vs nLUC-ABI5 + SRAS1.1-cLUC), 0.9876 (nLUC-DSK2A + cLUC vs nLUC-DSK2B + cLUC), 0.4335 (nLUC-DSK2A + cLUC vs nLUC-ABI5 + SRAS1.1-cLUC), 0.4996 (nLUC-DSK2B + cLUC vs nLUC-ABI5 + SRAS1.1-cLUC).

Appendix Figure S4

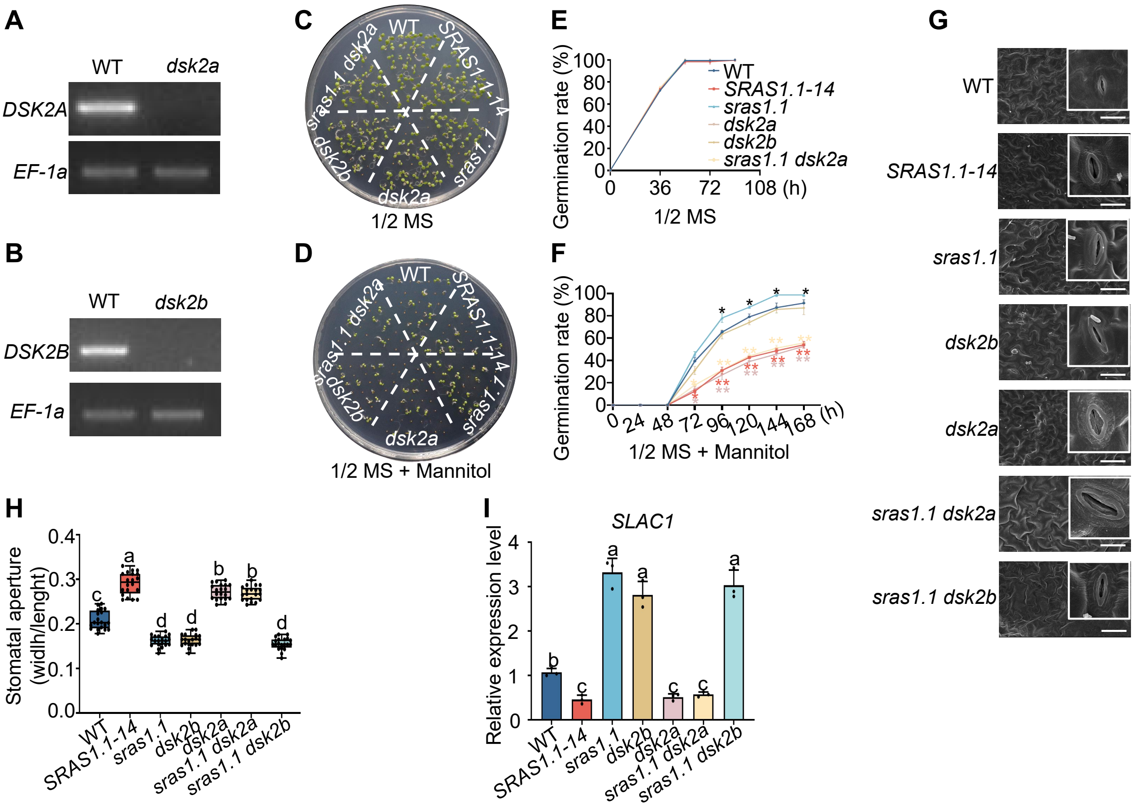

Appendix Figure S4. SRAS1.1 acts upstream of DSK2A under drought stress.

(A, B) RT-PCR analysis showing the expression levels of *DSK2A* (A) and *DSK2B* (B) in wild-type, *dsk2a* and *dsk2b* mutant plants. Elongation factor 1α (*EF-1α*) was used as an internal control.

(C–F) Phenotypic analysis of wild-type and transgenic plants grown on 1/2 MS medium under normal condition (C) and with 250 mM mannitol (D). Images were taken 7 days after germination. Relative germination rates under normal conditions (E) and mannitol treatment (F) were calculated. Values shown are means ± SD (n = 3 biological replicates). (E) T = 36 h: *P* values > 0.9999 (wild-type vs *SRAS1.1-14*), 0.8821 (wild-type vs *sras1.1*), 0.9976 (wild-type vs *dsk2a*), 0.9804 (wild-type vs *dsk2b*), 0.9419 (wild-type vs *sras1.1 dsk2a*). (the following is the same order). T = 72 h: *P* values = 0.8325, 0.9984, >0.9999, >0.9999, 0.9984. T = 96 h: *P* values = 0.538, >0.9999, >0.9999, >0.9999, >0.9999. (F) T = 48 h: *P* values > 0.9999, >0.9999, >0.9999, >0.9999, >0.9999. T = 72 h: *P* values = 0.0401, 0.1187, 0.022, 0.0969, 0.0481. T = 96 h: *P* values = 0.0033, 0.0155, 0.0024, 0.9728, 0.0052. T = 120 h: *P* values = 0.003, 0.0235, 0.0015, 0.3522, 0.0067. T=144 h: *P* values = 0.0032, 0.0226, 0.0012, 0.9699, 0.0042, T = 168 h: *P* values = 0.0047, 0.0121, 0.0037, 0.579, 0.0044.

(G, H) Representative images (G) and quantification (H) of stomatal apertures in wild-type and transgenic plants after drought stress. Scale bars = 20 μm. Data represent mean ± SD (n = 3 biological replicates), with 20 plants analyzed per replicate. (H) *P* values < 0.0001 (wild-type vs *SRAS1.1-14*), <0.0001 (wild-type vs *sras1.1*), <0.0001 (wild-type vs *dsk2b*), <0.0001 (wild-type vs *dsk2a*), <0.0001 (wild-type vs *sras1.1 dsk2a*), <0.0001 (wild-type vs *sras1.1 dsk2b*), <0.0001 (*SRAS1.1-14* vs *sras1.1*), <0.0001 (*SRAS1.1-14* vs *dsk2b*), 0.0147 (*SRAS1.1-14* vs *dsk2a*), 0.0192 (*SRAS1.1-14* vs *sras1.1 dsk2a*), <0.0001 (*SRAS1.1-14* vs *sras1.1 dsk2b*), 0.9998 (*sras1.1* vs *dsk2b*), <0.0001 (*sras1.1* vs *dsk2a*), <0.0001 (*sras1.1* vs *sras1.1 dsk2a*), 0.9489 (*sras1.1* vs *sras1.1 dsk2b*), <0.0001 (*dsk2b* vs *dsk2a*), <0.0001 (*dsk2b* vs *sras1.1 dsk2a*), 0.8103 (*dsk2b* vs *sras1.1 dsk2b*), 0.9967 (*dsk2a* vs *sras1.1 dsk2a*), <0.0001 (*dsk2a* vs *sras1.1 dsk2b*), <0.0001 (*sras1.1 dsk2a* vs *sras1.1 dsk2b*).

(I) Quantitative measurement of *SLAC1* expression in wild-type and transgenic plants. *UBQ10* was used as a reference gene. Values shown are means ± SD (n = 3 biological replicates). *P* values = 0.0009 (wild-type vs *SRAS1.1-14*), <0.0001 (wild-type vs *sras1.1*), <0.0001 (wild-type vs *dsk2b*), 0.0162 (wild-type vs *dsk2a*), 0.0257 (wild-type vs *sras1.1 dsk2a*), <0.0001 (wild-type vs *sras1.1 dsk2b*), <0.0001 (*SRAS1.1-14* vs *sras1.1*), <0.0001 (*SRAS1.1-14* vs *dsk2b*), >0.9999 (*SRAS1.1-14* vs *dsk2a*), 0.9934 (*SRAS1.1-14* vs *sras1.1 dsk2a*), <0.0001 (*SRAS1.1-14* vs *sras1.1 dsk2b*), 0.1418 (*sras1.1* vs *dsk2b*), <0.0001 (*sras1.1* vs *dsk2a*), <0.0001 (*sras1.1* vs *sras1.1 dsk2a*), 0.6851 (*sras1.1* vs *sras1.1 dsk2b*), <0.0001 (*dsk2b* vs *dsk2a*), <0.0001 (*dsk2b* vs *sras1.1 dsk2a*), 0.9221 (*dsk2b* vs *sras1.1 dsk2b*), 0.9998 (*dsk2a* vs *sras1.1 dsk2a*), <0.0001 (*dsk2a* vs *sras1.1 dsk2b*), <0.0001 (*sras1.1 dsk2a* vs *sras1.1 dsk2b*).

Data information: For (E, F) asterisks represent significant differences determined by Student's *t*-test (**P* < 0.05; ***P* < 0.01). For (H, I) different lowercase letters represent significant differences, as determined by one-way ANOVA in combination with Tukey's multiple comparisons test (*P* < 0.05). Data in (H) are plotted with box–whisker plots: the whiskers represent maximum and minimum values, and boxes represent the upper quartile, median, and lower quartile, dots represent data points.

Appendix Figure S5

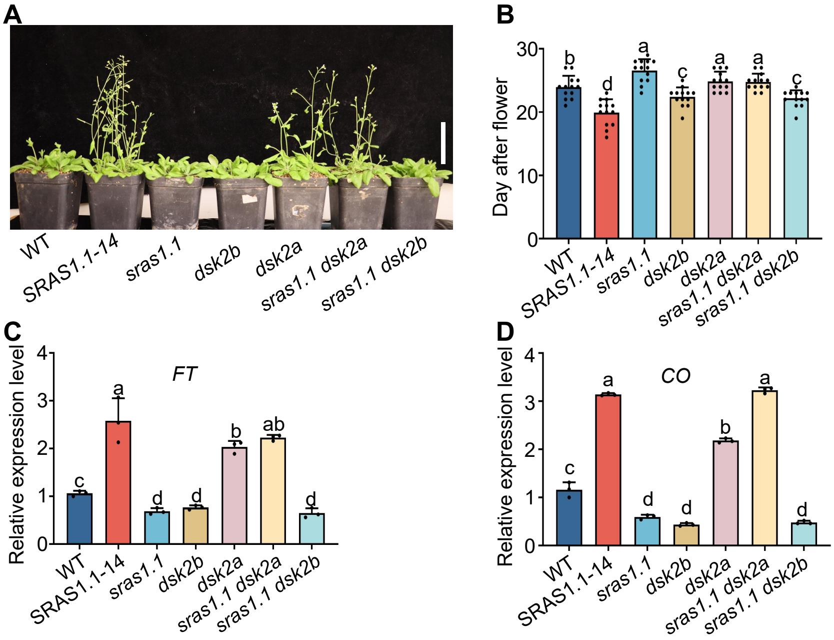

Appendix Figure S5. SRAS1.1 and DSK2A mediate *Arabidopsis* flowering time.

(A) Morphological comparison of wild-type, mutant lines, and transgenic plants grown in soil under identical conditions. Scale bar = 5 cm.
(B) Days to flowering in wild-type, mutant lines, and transgenic plants. Values shown are means ± SD (n = 3 biological replicates). *P* values < 0.0001 (wild-type vs *SRAS1.1-14*), 0.0002 (wild-type vs *sras1.1*), 0.0349 (wild-type vs *dsk2b*), 0.0122 (wild-type vs *dsk2a*), 0.0428 (wild-type vs *sras1.1 dsk2a*), 0.0485 (wild-type vs *sras1.1 dsk2b*), <0.0001 (*SRAS1.1-14* vs *sras1.1*), 0.0352 (*SRAS1.1-14* vs *dsk2b*), <0.0001 (*SRAS1.1-14* vs *dsk2a*), <0.0001 (*SRAS1.1-14* vs *sras1.1 dsk2a*), 0.0274 (*SRAS1.1-14* vs *sras1.1 dsk2b*), <0.0001 (*sras1.1* vs *dsk2b*), 0.1298 (*sras1.1* vs *dsk2a*), 0.0985 (*sras1.1* vs *sras1.1 dsk2a*), <0.0001 (*sras1.1* vs *sras1.1 dsk2b*), 0.0045 (*dsk2b* vs *dsk2a*), 0.0066 (*dsk2b* vs *sras1.1 dsk2a*), 0.9998 (*dsk2b* vs *sras1.1 dsk2b*), 0.9249 (*dsk2a* vs *sras1.1 dsk2a*), 0.0013 (*dsk2a* vs *sras1.1 dsk2b*), 0.002 (*sras1.1 dsk2a* vs *sras1.1 dsk2b*).
(C, D) Quantitative measurement of the expression level of *FLOWERING LOCUS T* (*FT*) (C) and *CONSTANS* (*CO*) (D) in the wild-type and all transgenic plants. Expression levels were normalized to *UBQ10.* Values shown are means ± SD (n = 3 biological replicates). (C) *P* values < 0.0001 (wild-type vs *SRAS1.1-14*), 0.0281 (wild-type vs *sras1.1*), 0.0339 (wild-type vs *dsk2b*), 0.0004 (wild-type vs *dsk2a*), <0.0001 (wild-type vs *sras1.1 dsk2a*), 0.0191 (wild-type vs *sras1.1 dsk2b*), <0.0001 (*SRAS1.1-14* vs *sras1.1*), <0.0001 (*SRAS1.1-14* vs *dsk2b*), 0.0457 (*SRAS1.1-14* vs *dsk2a*), 0.3351 (*SRAS1.1-14* vs *sras1.1 dsk2a),* <0.0001 (*SRAS1.1-14* vs *sras1.1 dsk2b*), 0.9983 (*sras1.1* vs *dsk2b*), <0.0001 (*sras1.1* vs *dsk2a*), <0.0001 (*sras1.1* vs *sras1.1 dsk2a*), >0.9999 (*sras1.1* vs *sras1.1 dsk2b*), <0.0001 (*dsk2b* vs *dsk2a*), <0.0001 (*dsk2b* vs *sras1.1 dsk2a*), 0.9853 (*dsk2b* vs *sras1.1 dsk2b*), 0.8729 (*dsk2a* vs *sras1.1 dsk2a*), <0.0001 (*dsk2a* vs *sras1.1 dsk2b*), <0.0001 (*sras1.1 dsk2a* vs *sras1.1 dsk2b*). (The following is the same order). (D) *P* values < 0.0001, 0.0021, <0.0001, <0.0001, <0.0001, <0.0001, <0.0001, <0.0001, 0.0021, 0.7366, <0.0001, 0.1622, <0.0001, <0.0001, 0.4709, <0.0001 , <0.0001, 0.986, 0.0013, <0.0001, <0.0001.
Data information: For (B, C, D) different lowercase letters represent significant differences, as determined by one-way ANOVA in combination with Tukey's multiple comparisons test (*P* < 0.05).

Appendix Figure S6

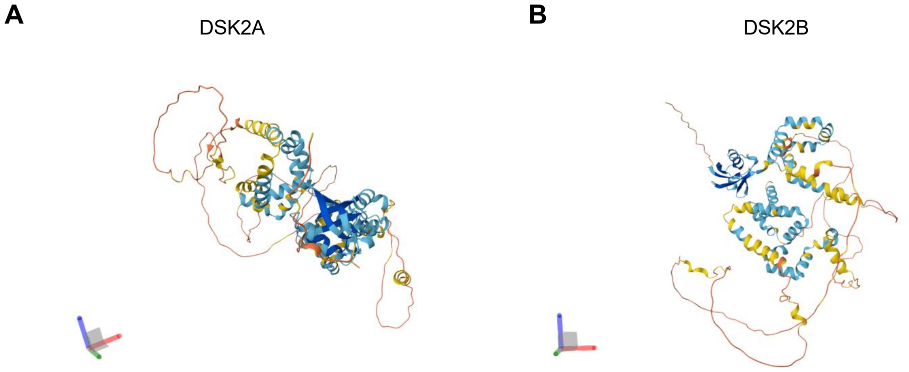

Appendix Figure S6. Predicted structural divergence between DSK2A and DSK2B.

(A, B) Predicted tertiary structures of DSK2A (A) and DSK2B (B) proteins generated using AlphaFold3. Structural models highlight potential regions of functional divergence between DSK2A and DSK2B.

Appendix Figure S7

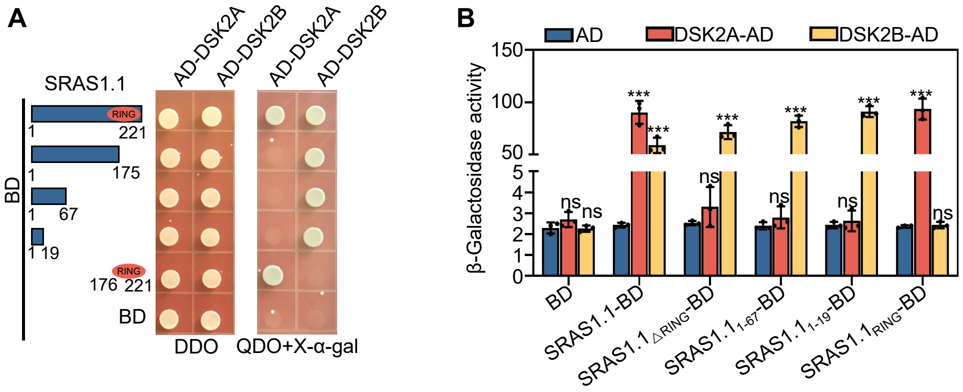

Appendix Figure S7. The RING domain is required for the interaction between SRAS1.1 and DSK2A.

(A) Domain mapping of SRAS1.1 to identify regions interacting with DSK2A and DSK2B. A series of SRAS1.1 deletion constructs were generated based on predicted domain boundaries, and interactions were examined using the yeast two-hybrid (Y2H) assay. (B) Measurement of β-gal activity (A), quantification of β-Gal activity after culturing yeast strains in liquid medium with o-nitrophenyl-β-D-galactoside as substrate. Values shown are means ± SD (n = 3 biological replicates). Asterisks represent significant differences determined by Student's *t*-test ($***P < 0.001$; ns, not significant $P \geq 0.05$). BD: P values = 0.1962 (AD vs DSK2A-AD), 0.8593 (AD vs DSK2B-AD). (the following is the same order). SRAS1.1-BD: P values < 0.0001, 0.0002. SRAS1.1$_{\triangle RING}$-BD: P values = 0.2366, <0.0001. SRAS1.1$_{1\text{-}67}$-BD: P values = 0.2813, <0.0001. SRAS1.1$_{1\text{-}19}$-BD: P values = 0.5391, <0.0001. SRAS1.1$_{RING}$-BD: P values < 0.0001, 0.5185.

Appendix Figure S8

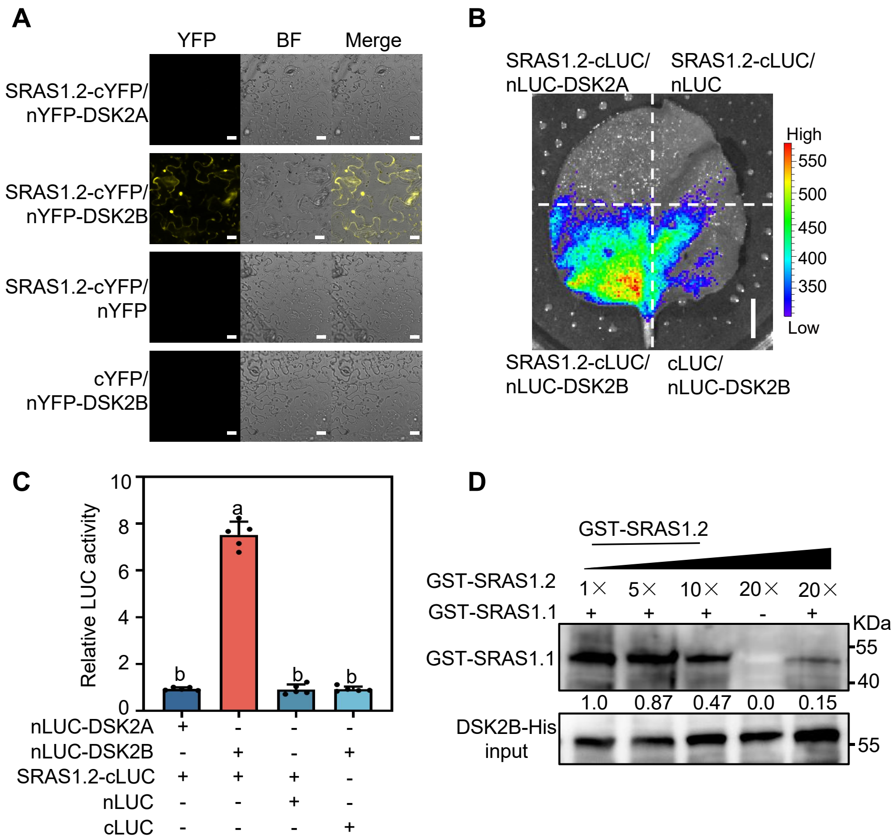

Appendix Figure S8. SRAS1.2 interacts with DSK2B to prevent its degradation.

(A) BiFC analysis showed that SRAS1.2 interacted with DSK2B but not DSK2A. GFP fluorescence was observed in the cytoplasm using confocal microscopy. Scale bars = 20 µm. The experiment was repeated three times with consistent results.
(B, C) LCI assay (B) and quantification of luciferase activity (C) demonstrated that SRAS1.2 interacts with DSK2B. Scale bar = 1 cm. Data represent means ± SD from five biological replicates (n = 5). Different lowercase letters represent significant differences, as determined by one-way ANOVA in combination with Tukey's multiple comparisons test ($P < 0.05$). (C) P values < 0.0001 (nLUC-DSK2A + SRAS1.2-cLUC vs nLUC-DSK2B + SRAS1.2-cLUC), >0.9999 (nLUC-DSK2A + SRAS1.2-cLUC vs nLUC + SRAS1.2-cLUC), >0.9999 (nLUC-DSK2A + SRAS1.2-cLUC vs nLUC-DSK2B + cLUC), <0.0001 (nLUC-DSK2B + SRAS1.2-cLUC vs nLUC + SRAS1.2-cLUC), <0.0001 (nLUC-DSK2B + SRAS1.2-cLUC vs nLUC-DSK2B + cLUC), >0.9999 (nLUC + SRAS1.2-cLUC vs nLUC-DSK2B + cLUC).
(D) *In vitro* competitive pull-down assay shows that SRAS1.2 alters the interaction between SRAS1.1 and DSK2B in a dose-dependent manner. SRAS1.1-GST proteins were incubated with SRAS1.2-GST and immobilized DSK2B-His. Immunoprecipitated fractions probed with anti-GST antibody. The gradient represents increasing amounts of SRAS1.2-GST. The lower panel shows DSK2A-His input.

Appendix Figure S9

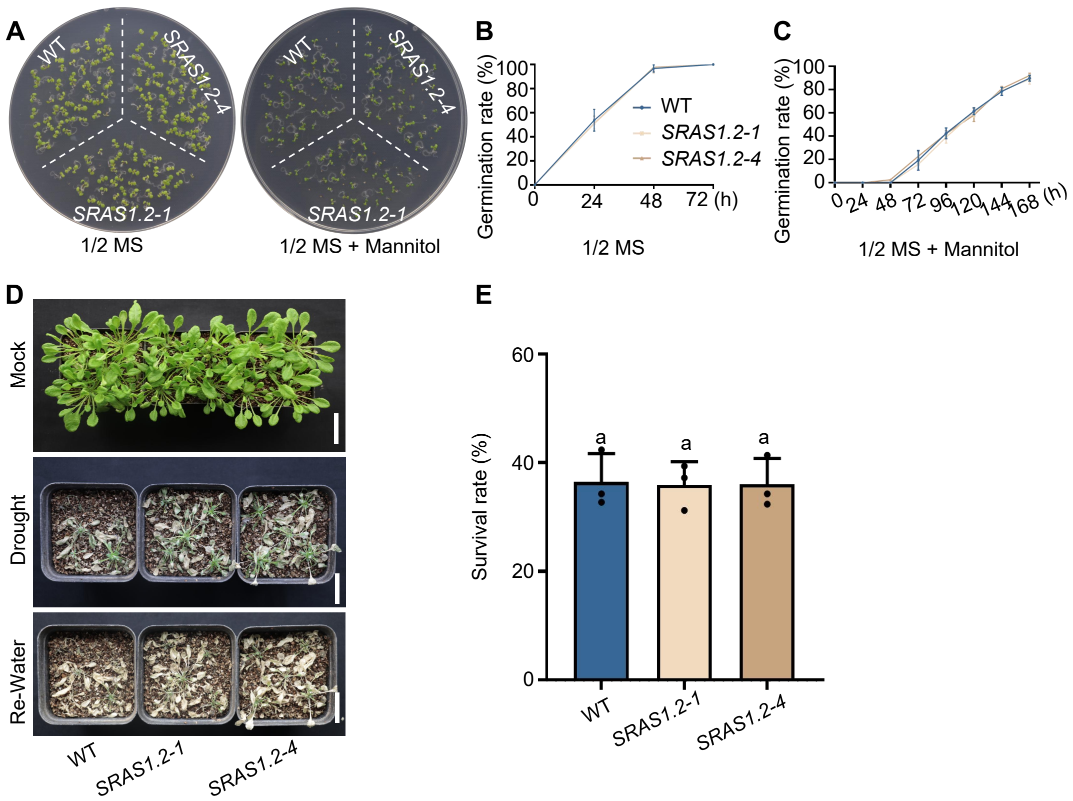

Appendix Figure S9. Drought stress response of *SRAS1.2*-overexpressing plants.

(A–C) Phenotypic analysis of wild-type, *SRAS1.2-1*, and S*RAS1.2-4* seedlings. (A) Plants were grown on 1/2 MS medium with or without 250 mM mannitol, and images were taken 7 days after germination. (B, C) Germination rates of wild-type and transgenic plants under normal conditions (B) and 250 mM mannitol treatment (C).Values shown are means ± SD (n = 3 biological replicates). Significance was determined using Student's *t*-test. (B) T = 24 h: *P* values = 0.8549 (wild-type vs *SRAS1.2-1*), >0.9999, (wild-type vs *SRAS1.2-4*). (the following is the same order). T = 48 h: *P* values = 0.9929, 0.9385. T = 72 h: *P* values = 0.972, 0.9987. (C) T = 48 h: *P* values > 0.9999, 0.0872, T = 72 h: *P* values = 0.7673, 0.771. T = 96 h: *P* values = 0.6264, 0.9949. T = 120 h: *P* values = 0.9582, 0.6423. T = 144 h: *P* values = 0.9582, 0.6164. T = 168 h: *P* values = 0.9155, 0.6367.
(D) Drought tolerance assay of wild-type, *SRAS1.2-1*, and *SRAS1.2-4* overexpressing plants. Seedlings were grown under normal conditions for 2 weeks, subjected to drought stress for 16 days, and then rewatered for 5 days. Scale bars = 2 cm.
(E) Survival rate of seedlings treated as in (D). Values shown are means ± SD (n = 3 biological replicates). Different lowercase letters represent significant differences, as determined by one-way ANOVA in combination with Tukey's multiple comparisons test, ($P < 0.05$). *P* values > 0.9999 (wild-type vs *SRAS1.2-1*), >0.9999 (wild-type vs *SRAS1.2-4*), >0.9999 (*SRAS1.2-1* vs *SRAS1.2-4*).
