# Supplementary figures and images for: SRAS1.1 E3 ligase mediates DSK2A degradation to regulate autophagy and drought tolerance in Arabidopsis
Source: EMBO Rep. 2025 Aug 22;26(19):4794–819. doi: 10.1038/s44319-025-00556-9 (PMC12508185; doi:10.1038/s44319-025-00556-9)

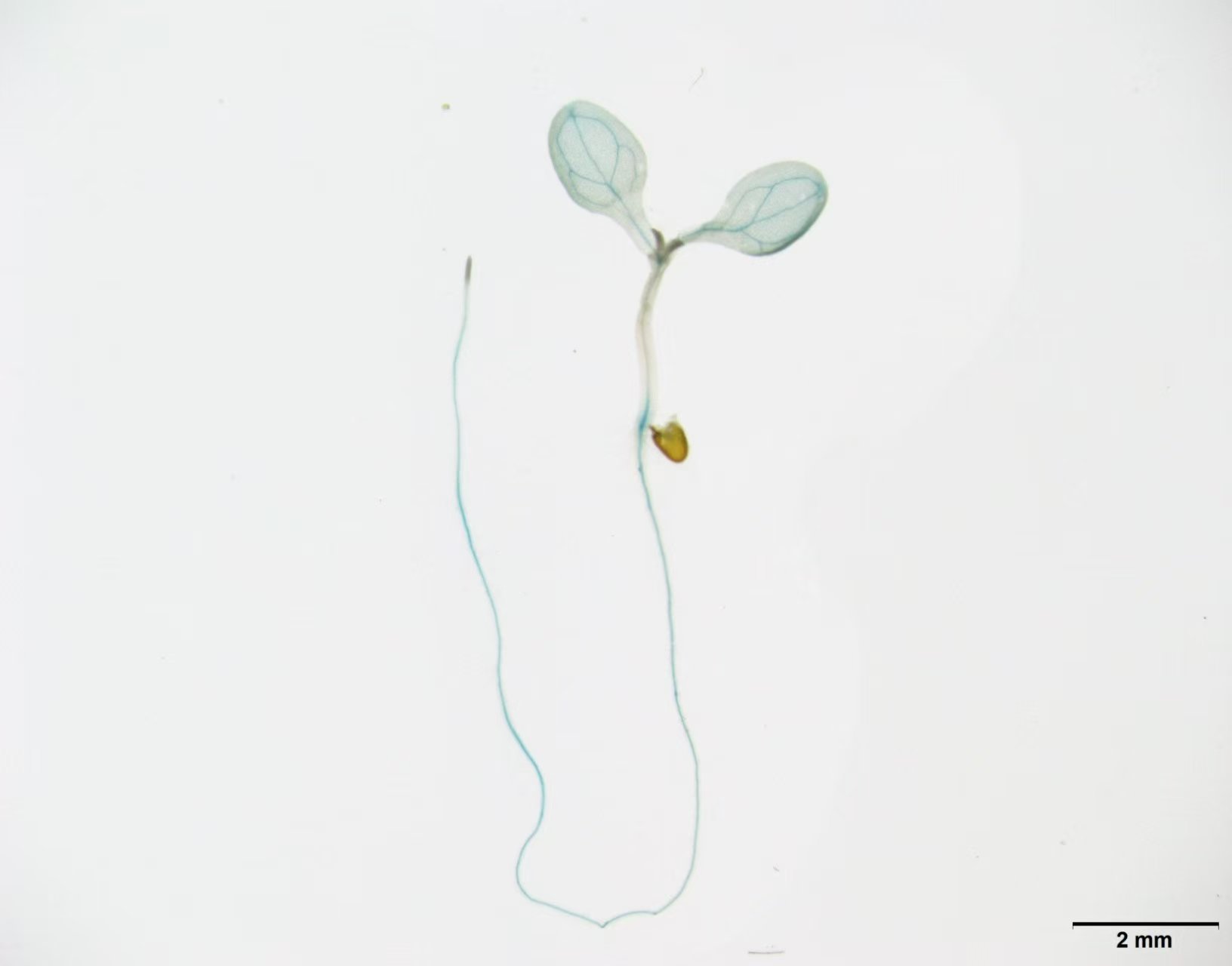

Supplement: Supplementary file 7 — Source data Fig. 1 [file 44319_2025_556_MOESM7_ESM.zip › Figure 1/1B/Drought stress.tif]

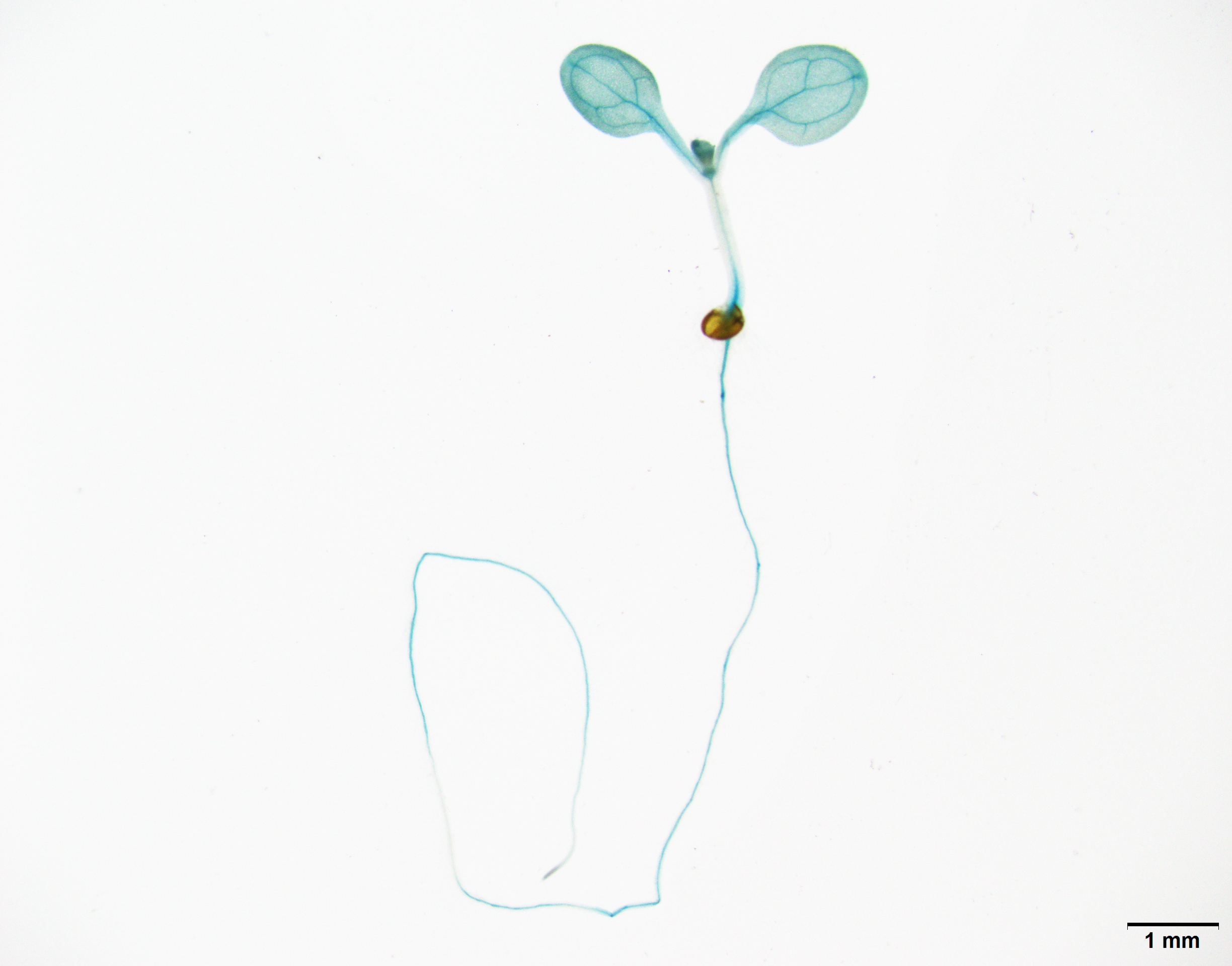

Supplement: Supplementary file 7 — Source data Fig. 1 [file 44319_2025_556_MOESM7_ESM.zip › Figure 1/1B/Mock.tif]

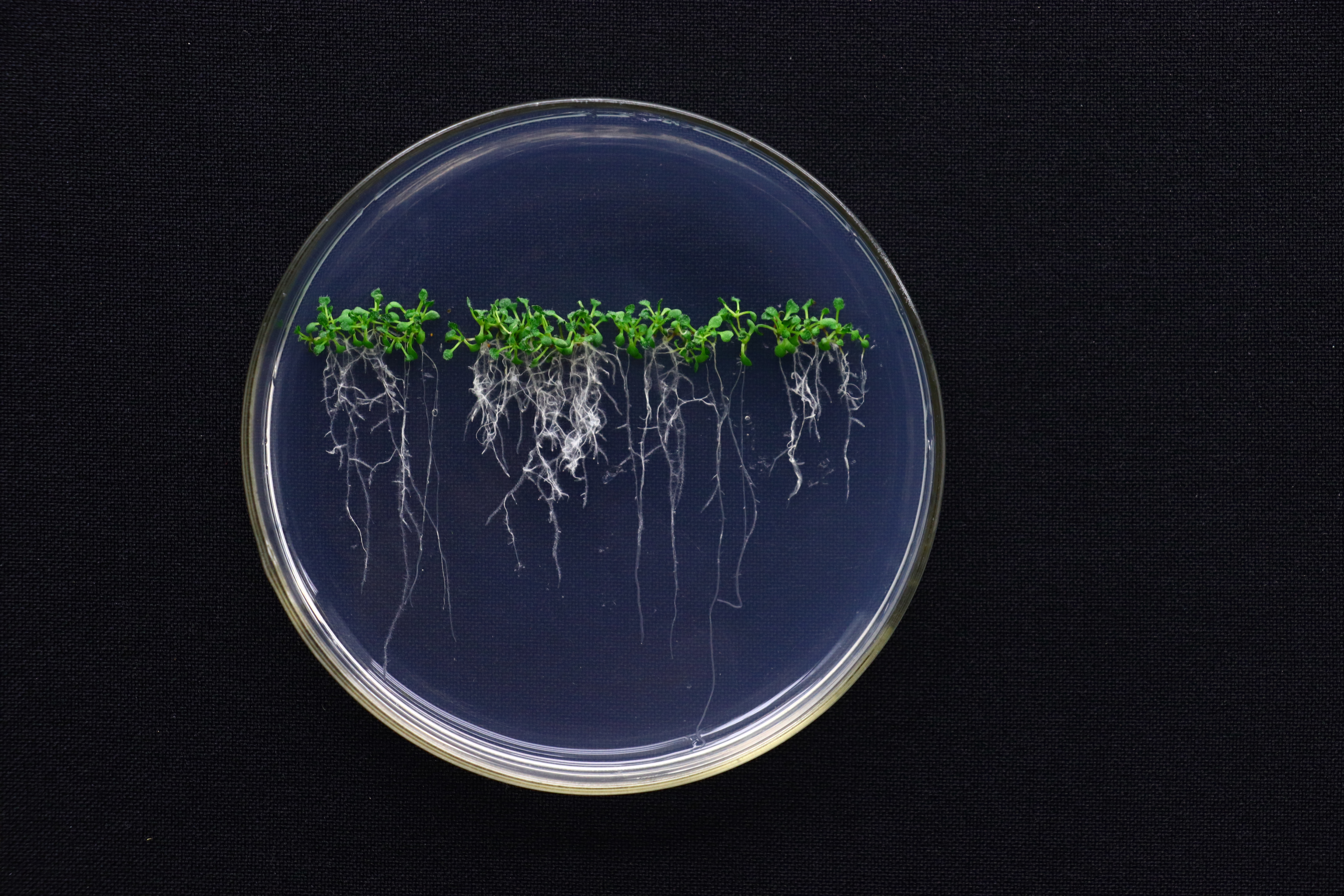

Supplement: Supplementary file 7 — Source data Fig. 1 [file 44319_2025_556_MOESM7_ESM.zip › Figure 1/1D/12 MS+ Mannitol.tif]

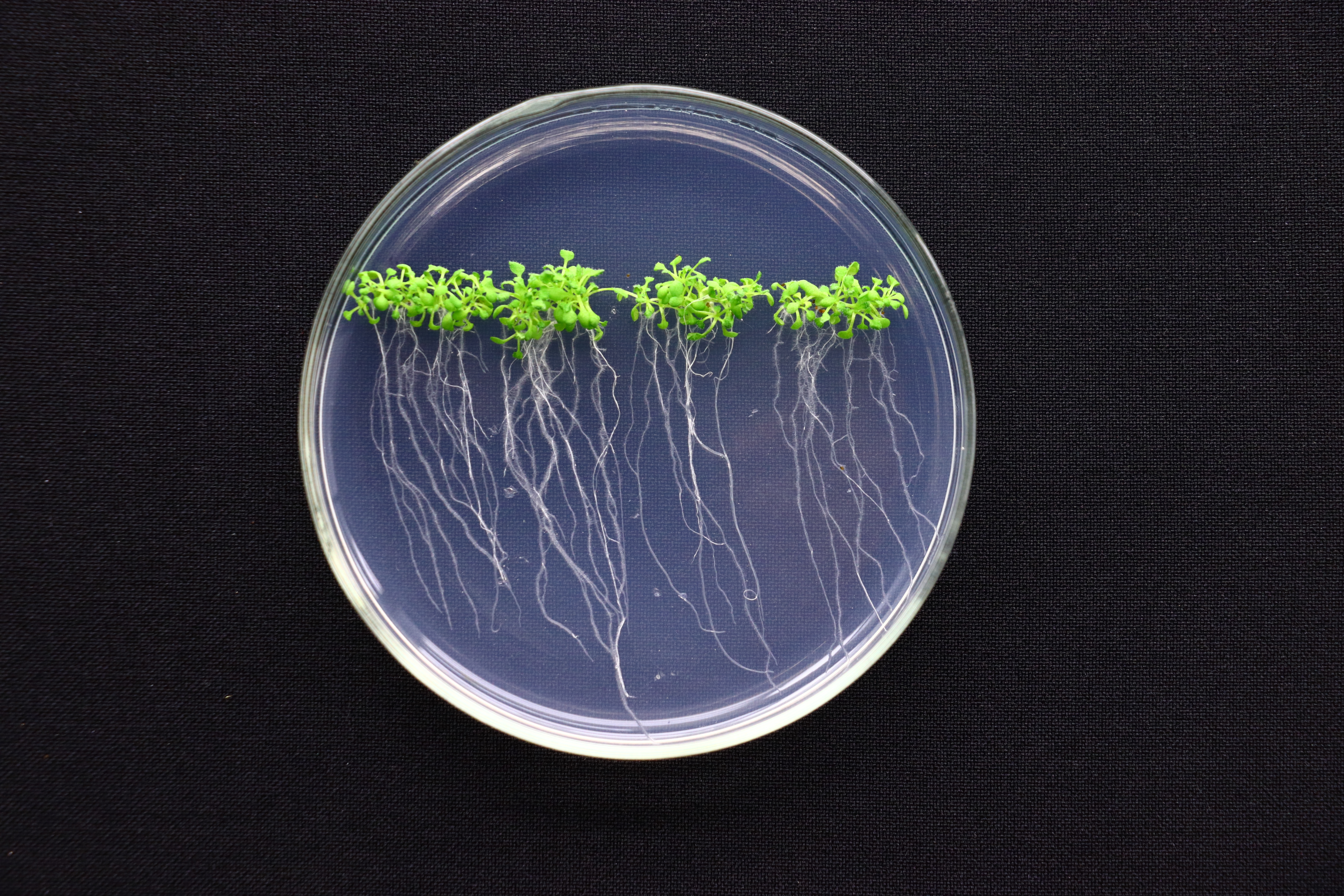

Supplement: Supplementary file 7 — Source data Fig. 1 [file 44319_2025_556_MOESM7_ESM.zip › Figure 1/1D/12MS.tif]

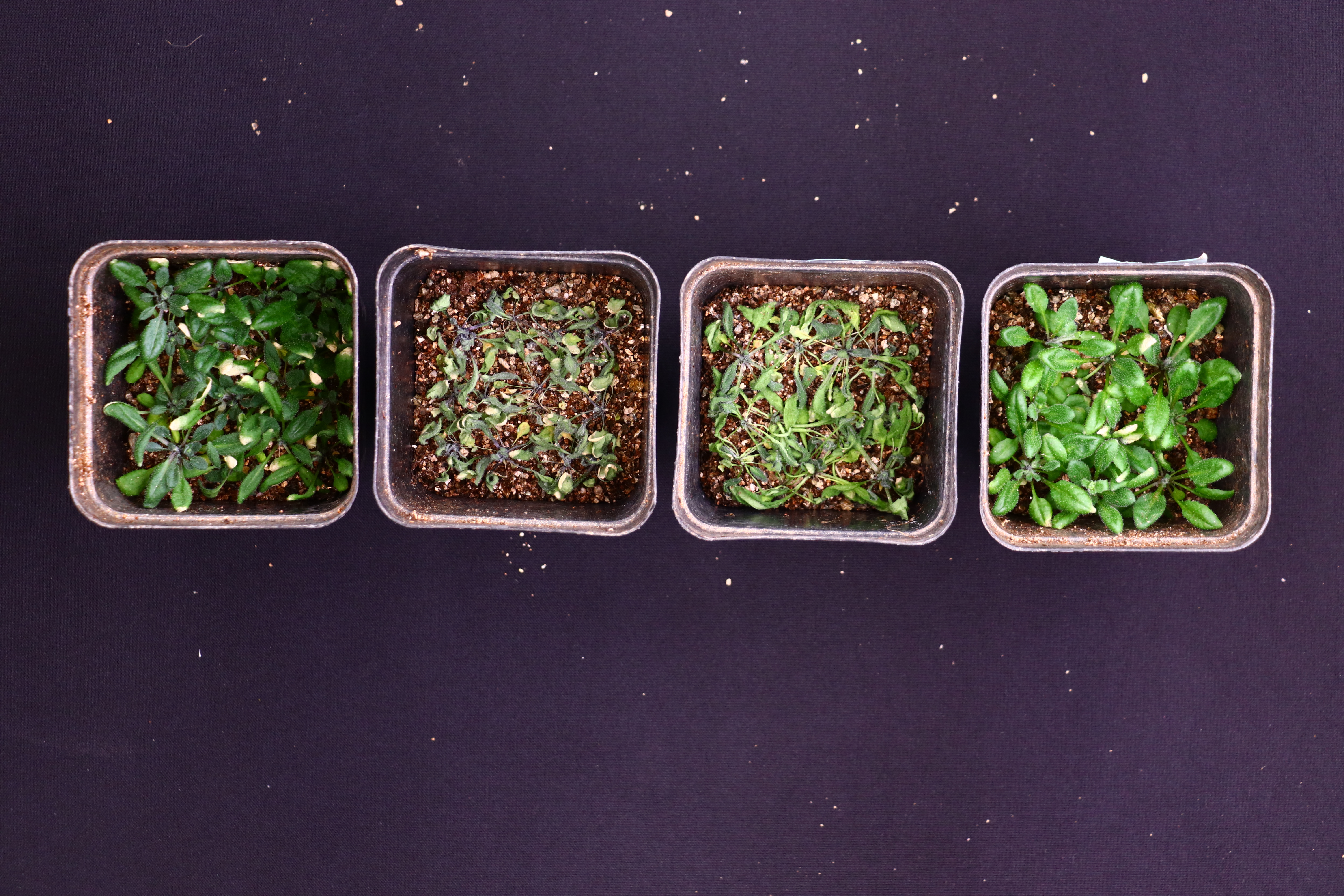

Supplement: Supplementary file 7 — Source data Fig. 1 [file 44319_2025_556_MOESM7_ESM.zip › Figure 1/1G/Drought.JPG]

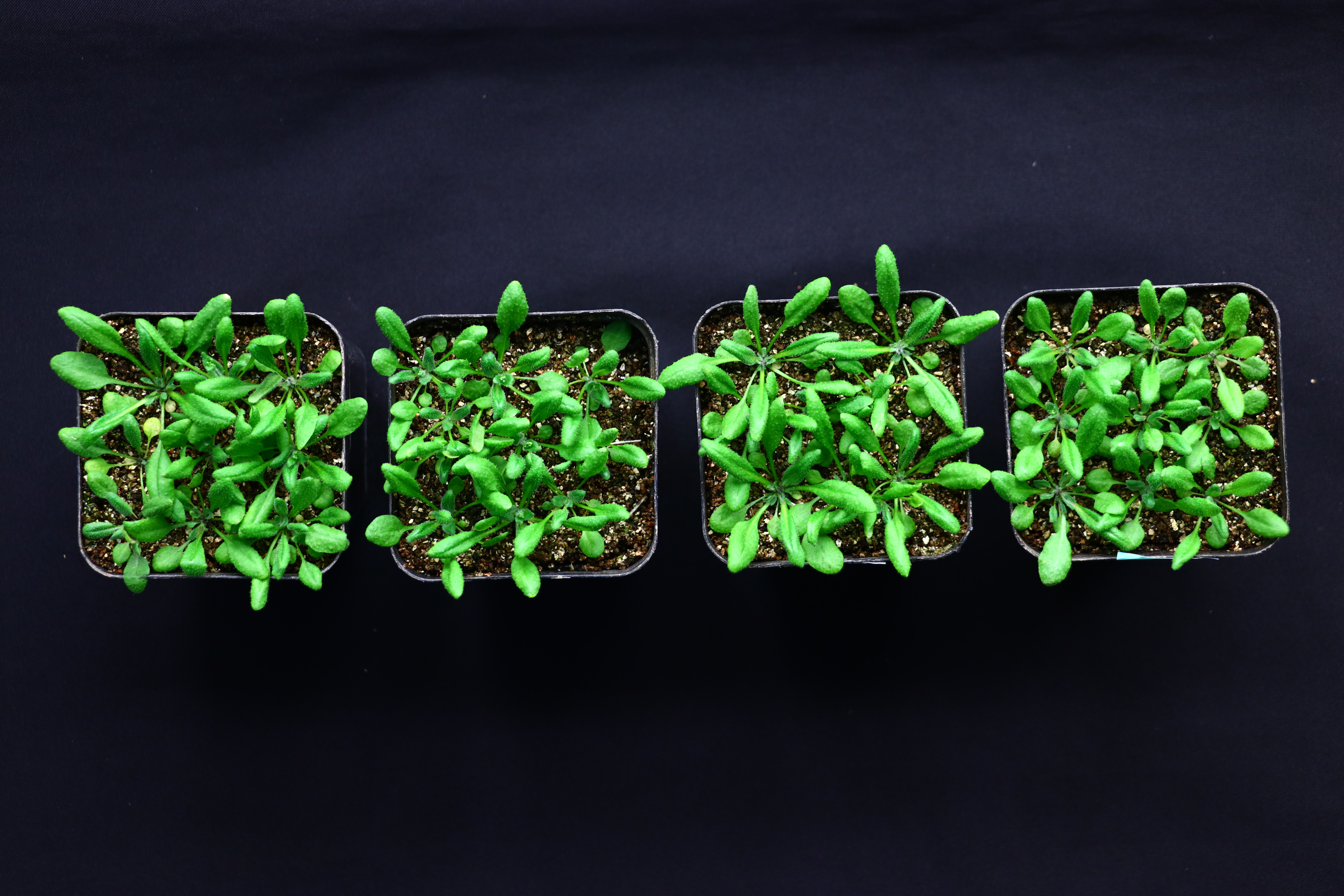

Supplement: Supplementary file 7 — Source data Fig. 1 [file 44319_2025_556_MOESM7_ESM.zip › Figure 1/1G/Mock.JPG]

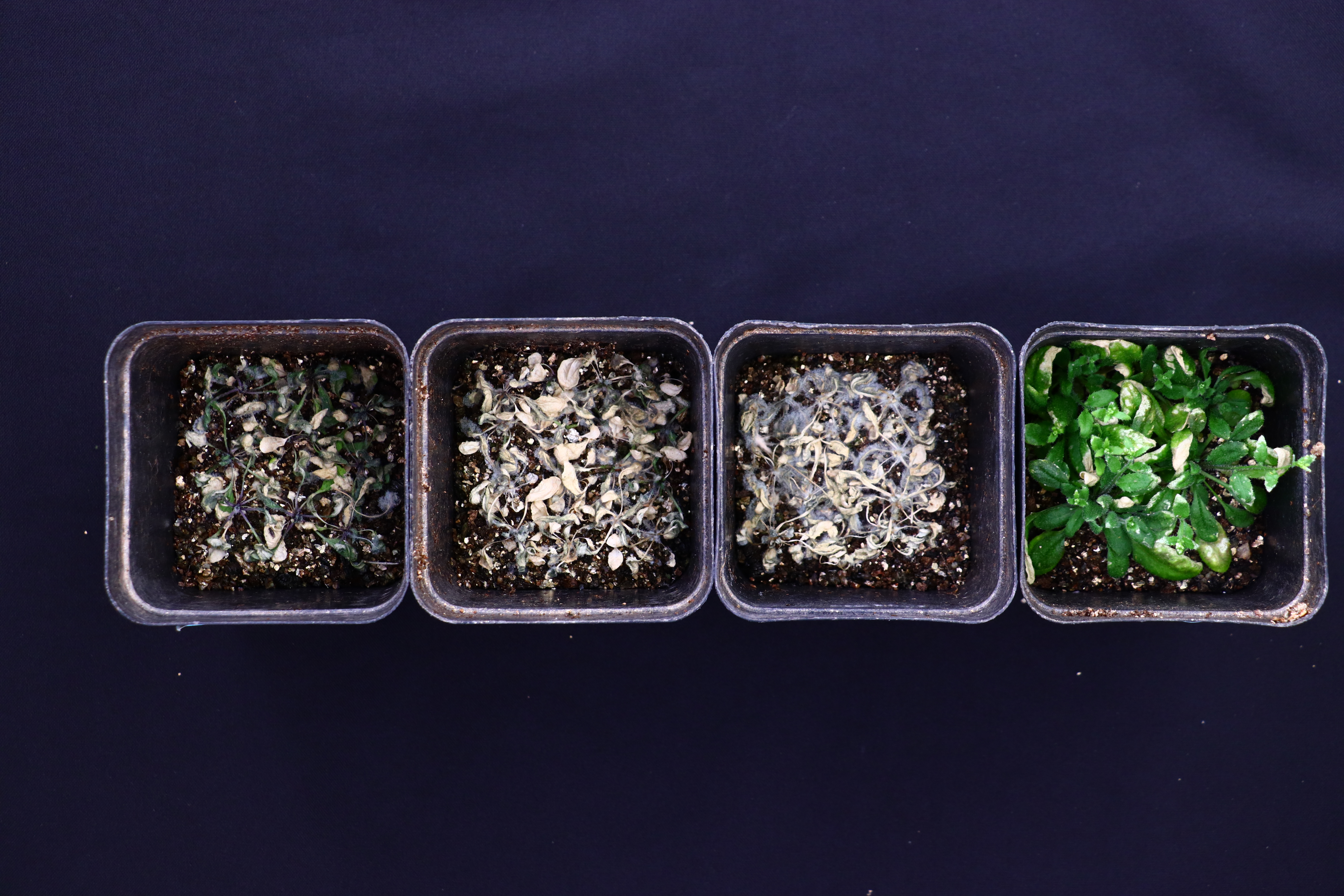

Supplement: Supplementary file 7 — Source data Fig. 1 [file 44319_2025_556_MOESM7_ESM.zip › Figure 1/1G/Re-water.JPG]

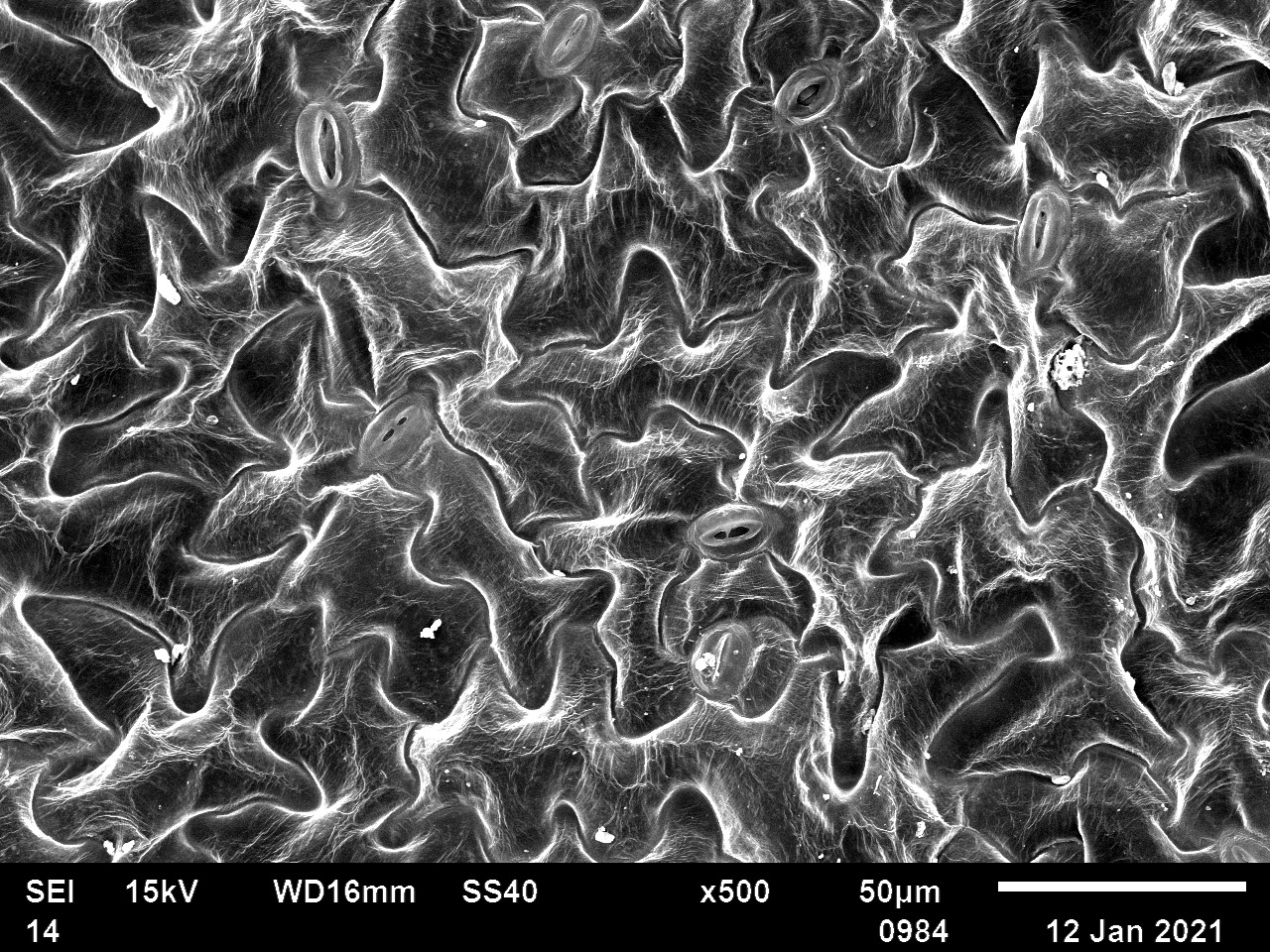

Supplement: Supplementary file 7 — Source data Fig. 1 [file 44319_2025_556_MOESM7_ESM.zip › Figure 1/1J/SRAS1.1-26 .tif]

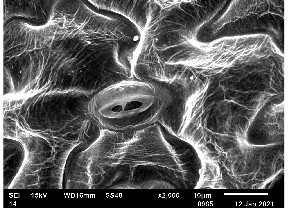

Supplement: Supplementary file 7 — Source data Fig. 1 [file 44319_2025_556_MOESM7_ESM.zip › Figure 1/1J/SRAS1.1-26-X.tif]

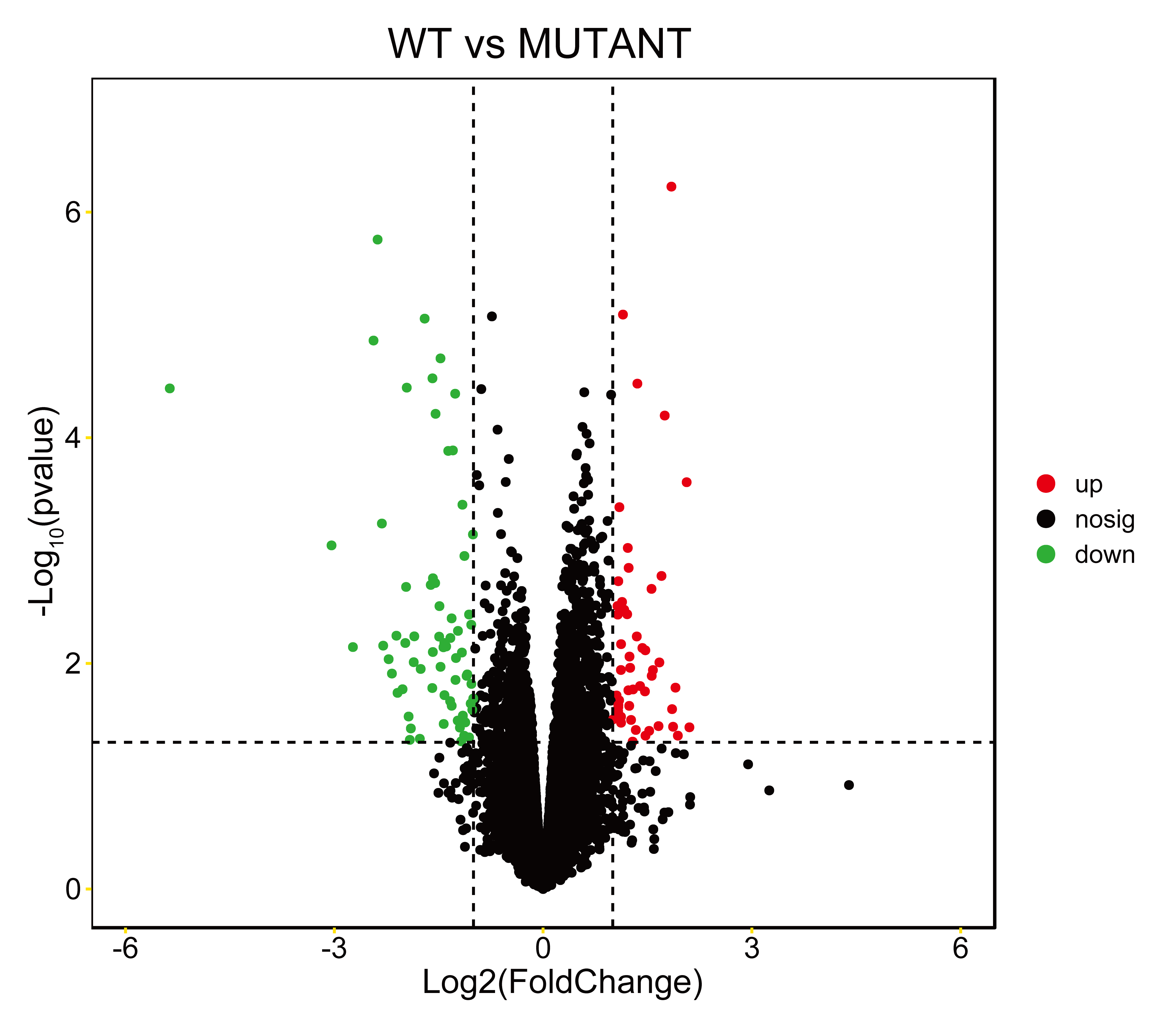

Supplement: Supplementary file 8 — Source data Fig. 2 [file 44319_2025_556_MOESM8_ESM.zip › Figure 2/2A/Mutant(1).tif]

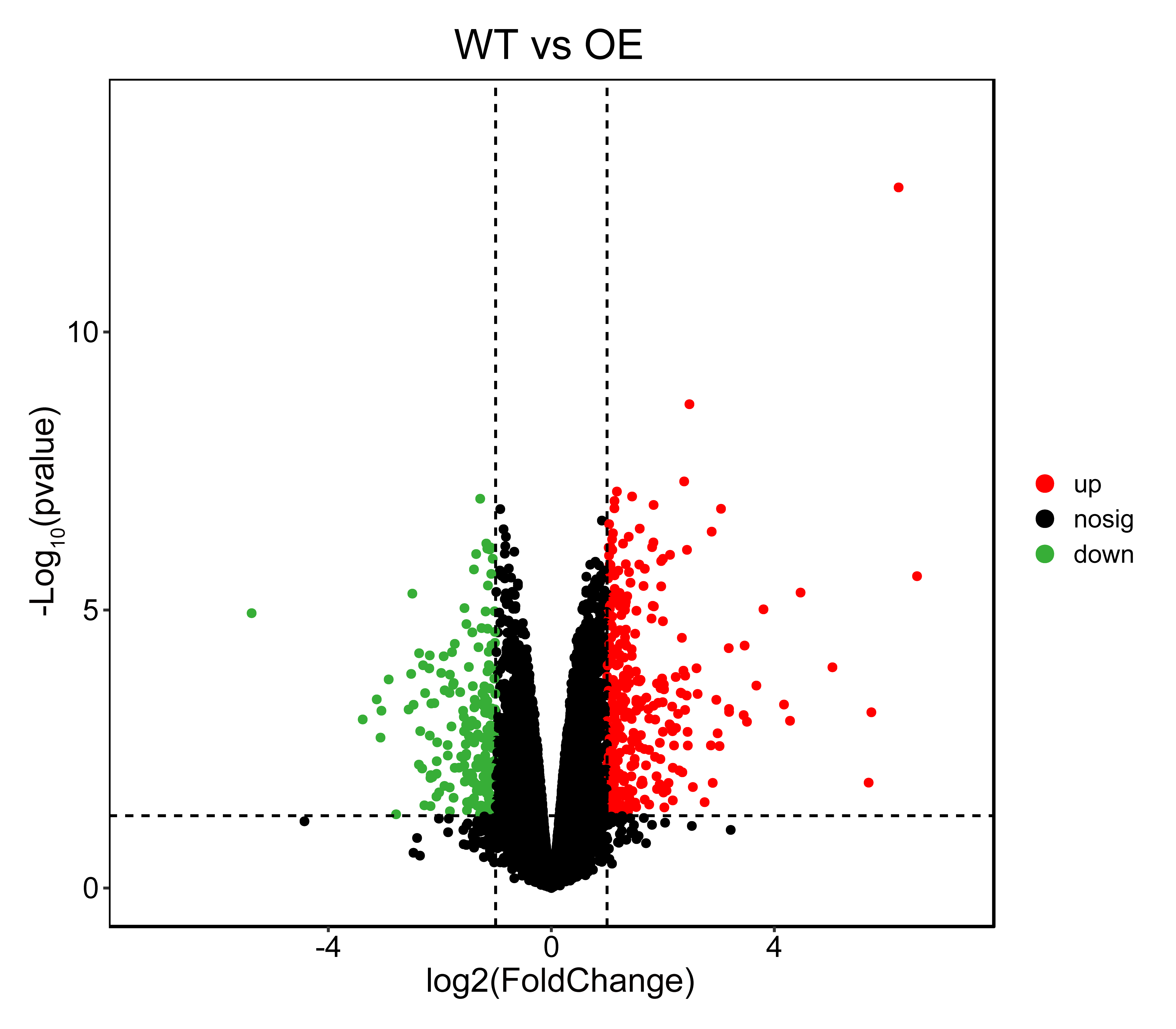

Supplement: Supplementary file 8 — Source data Fig. 2 [file 44319_2025_556_MOESM8_ESM.zip › Figure 2/2B/oe.tif]

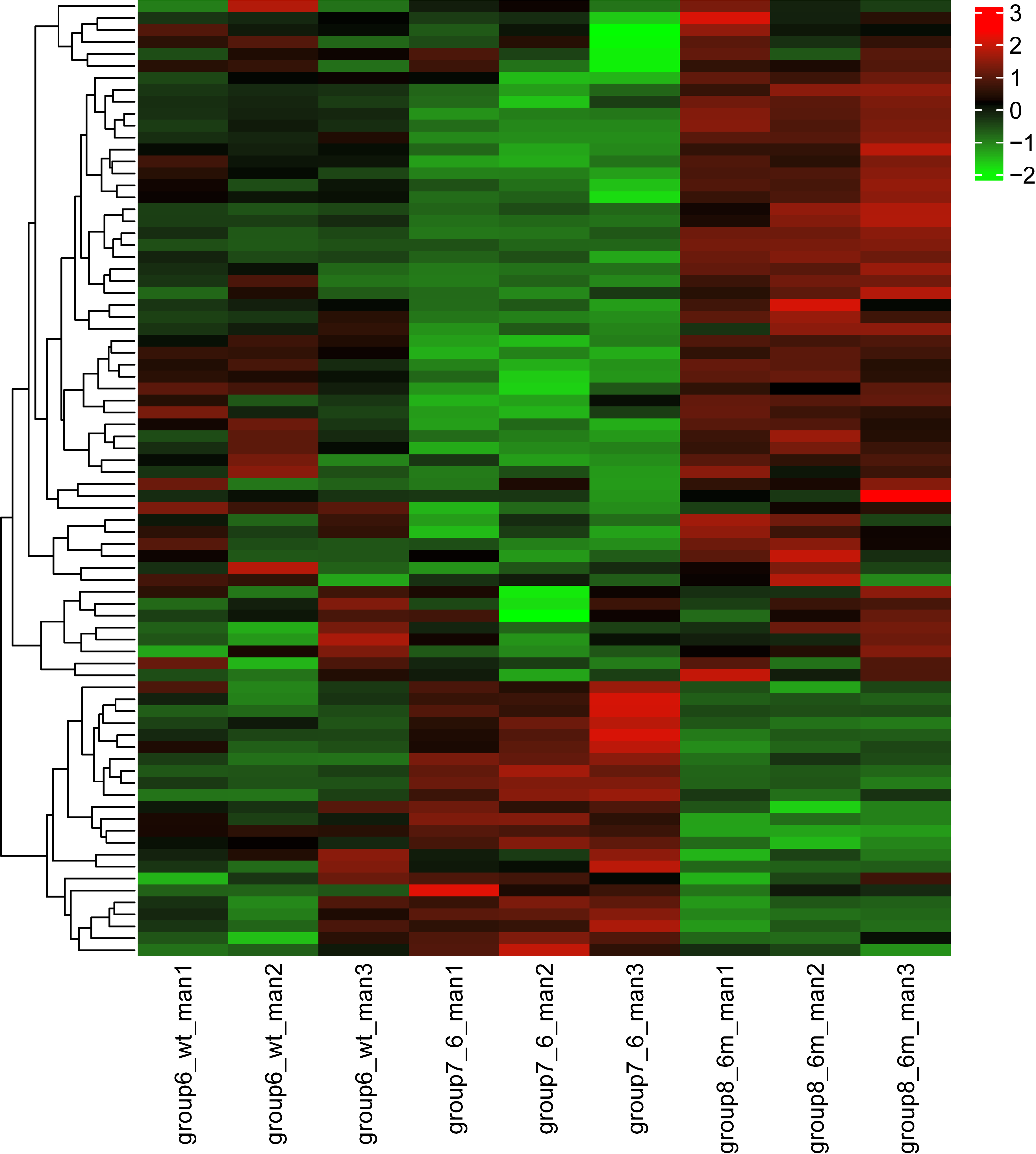

Supplement: Supplementary file 8 — Source data Fig. 2 [file 44319_2025_556_MOESM8_ESM.zip › Figure 2/2C/pheatmap2.tif]

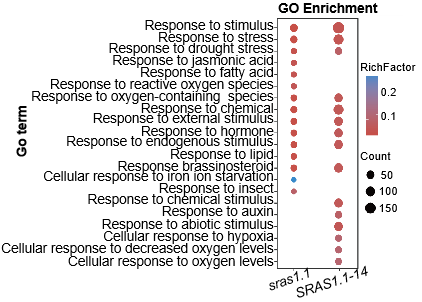

Supplement: Supplementary file 8 — Source data Fig. 2 [file 44319_2025_556_MOESM8_ESM.zip › Figure 2/2D/GO.tif]

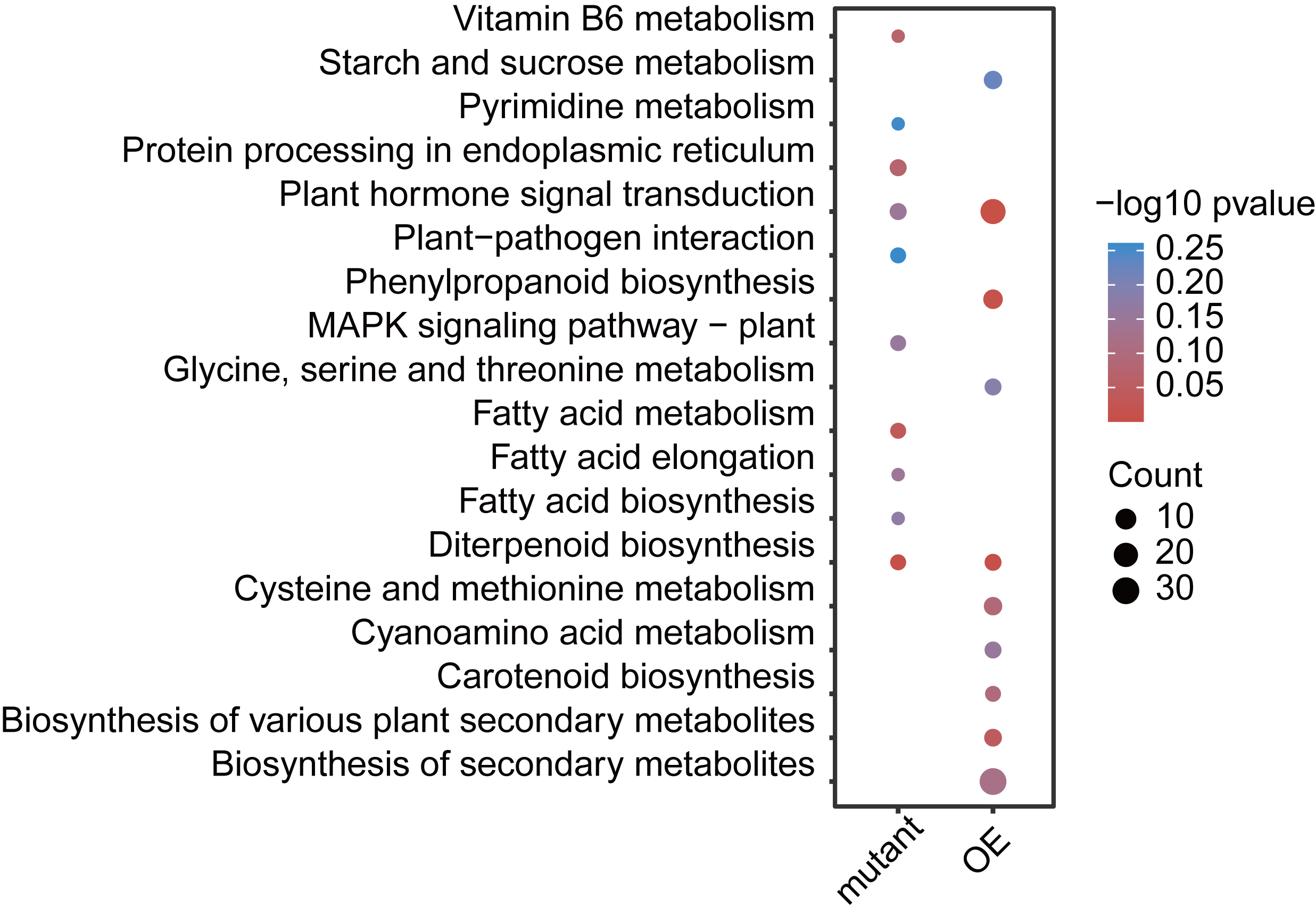

Supplement: Supplementary file 8 — Source data Fig. 2 [file 44319_2025_556_MOESM8_ESM.zip › Figure 2/2E/KEGG.tif]

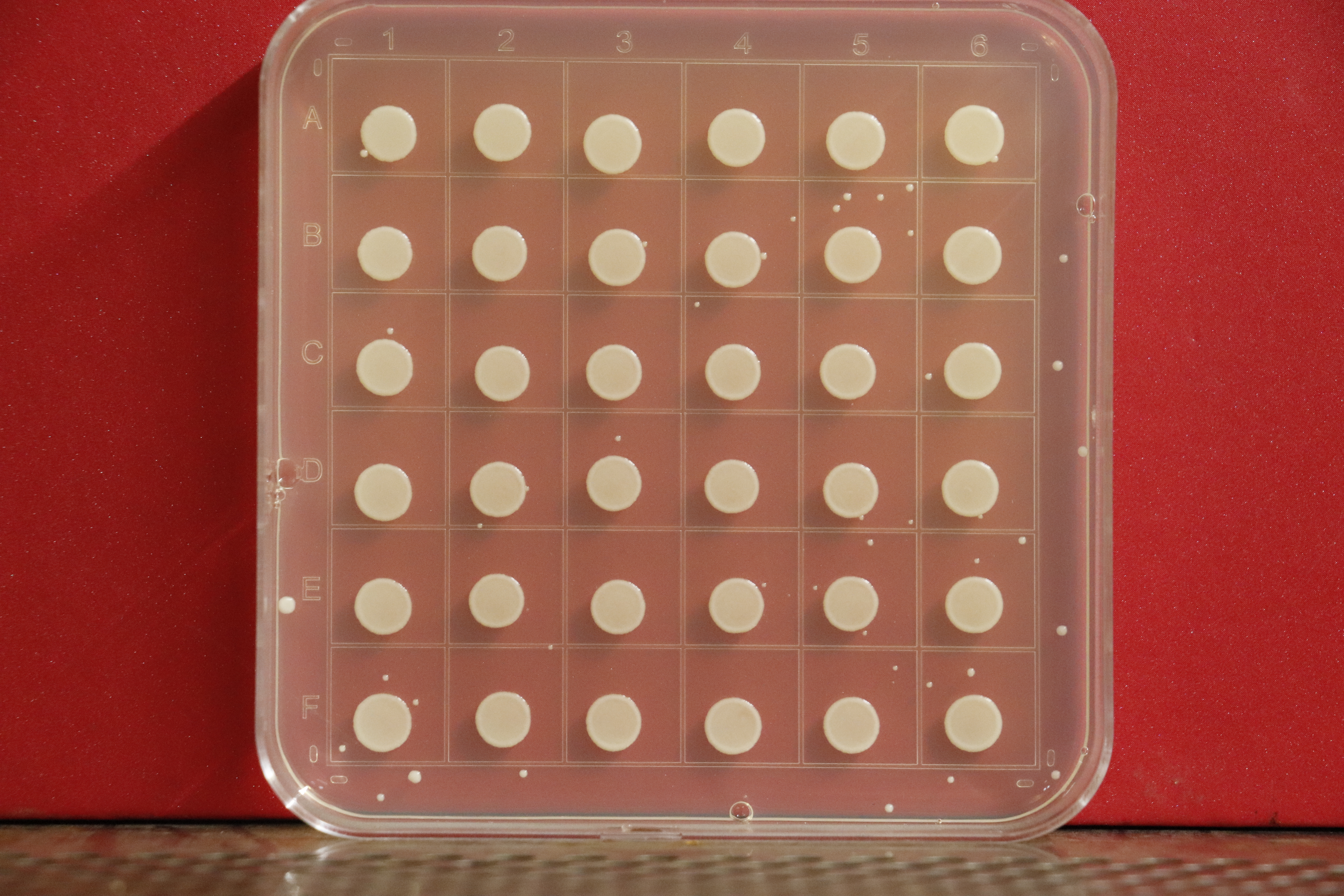

Supplement: Supplementary file 9 — Source data Fig. 3 [file 44319_2025_556_MOESM9_ESM.zip › Figure 3/3A/DDO.JPG]

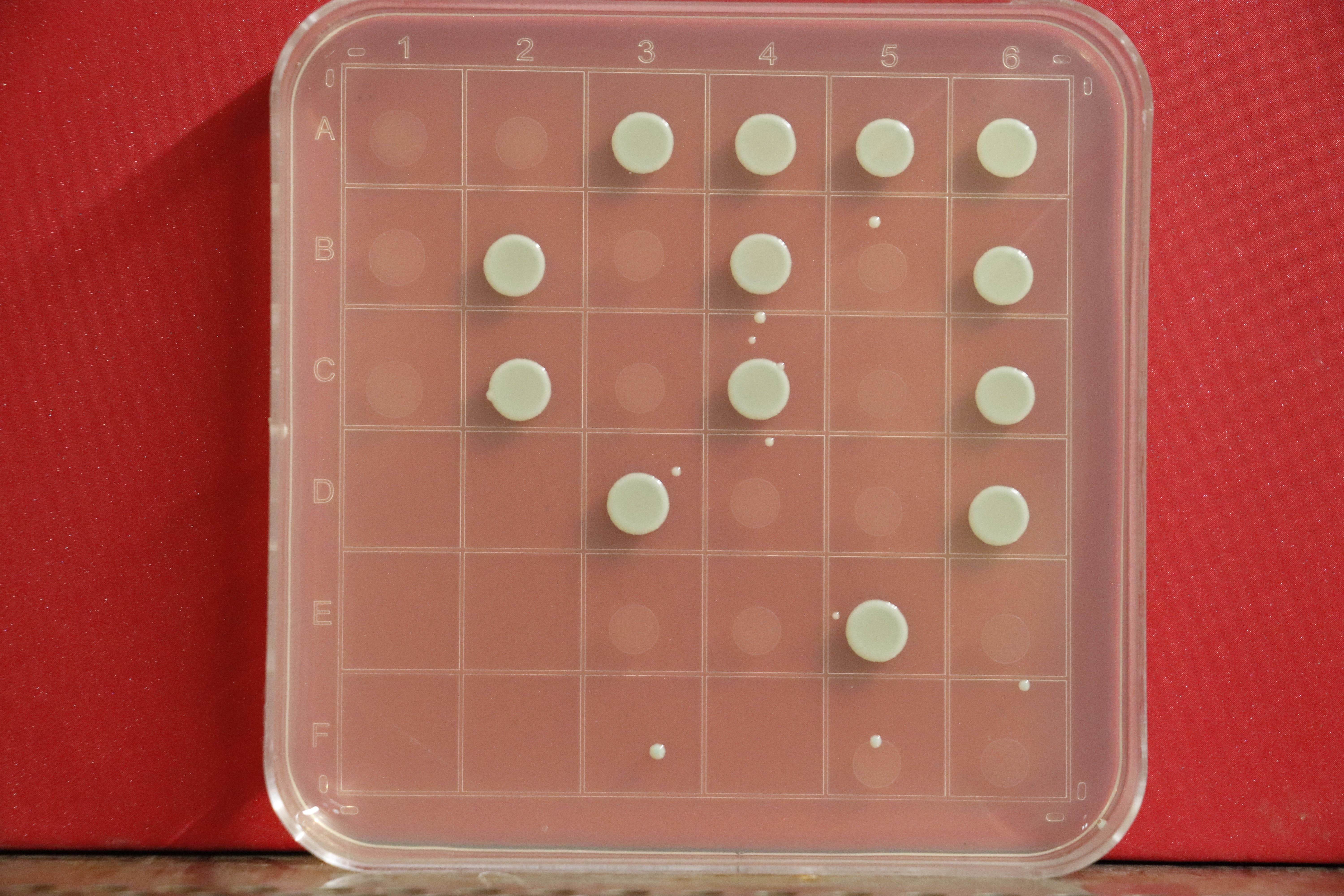

Supplement: Supplementary file 9 — Source data Fig. 3 [file 44319_2025_556_MOESM9_ESM.zip › Figure 3/3A/QDO.JPG]

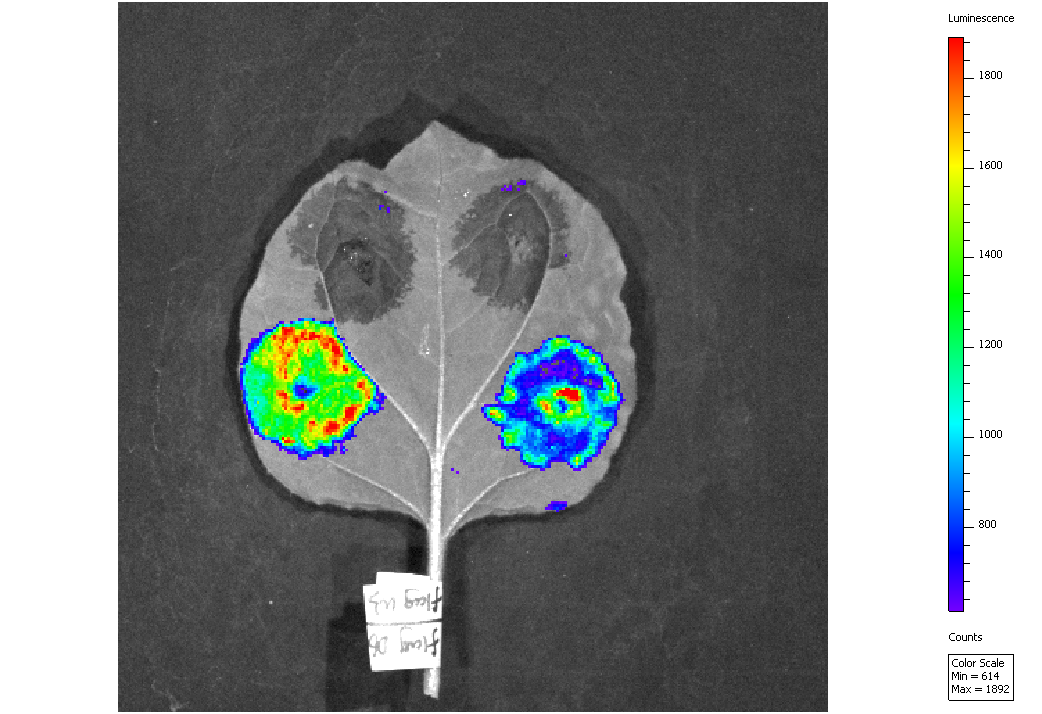

Supplement: Supplementary file 9 — Source data Fig. 3 [file 44319_2025_556_MOESM9_ESM.zip › Figure 3/3B/SRAS1.1+2A2B.tif]

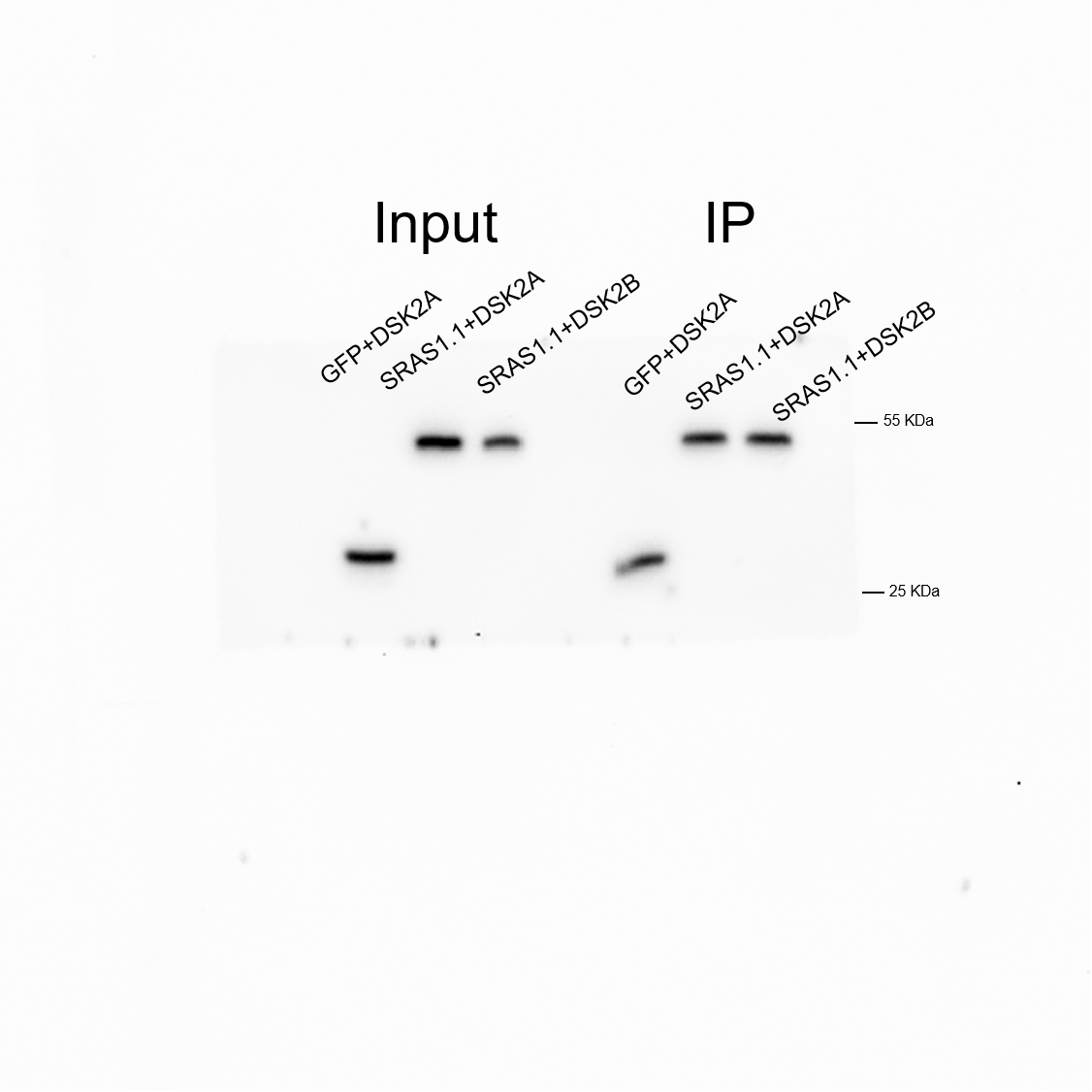

Supplement: Supplementary file 9 — Source data Fig. 3 [file 44319_2025_556_MOESM9_ESM.zip › Figure 3/3D/图片1.tif]

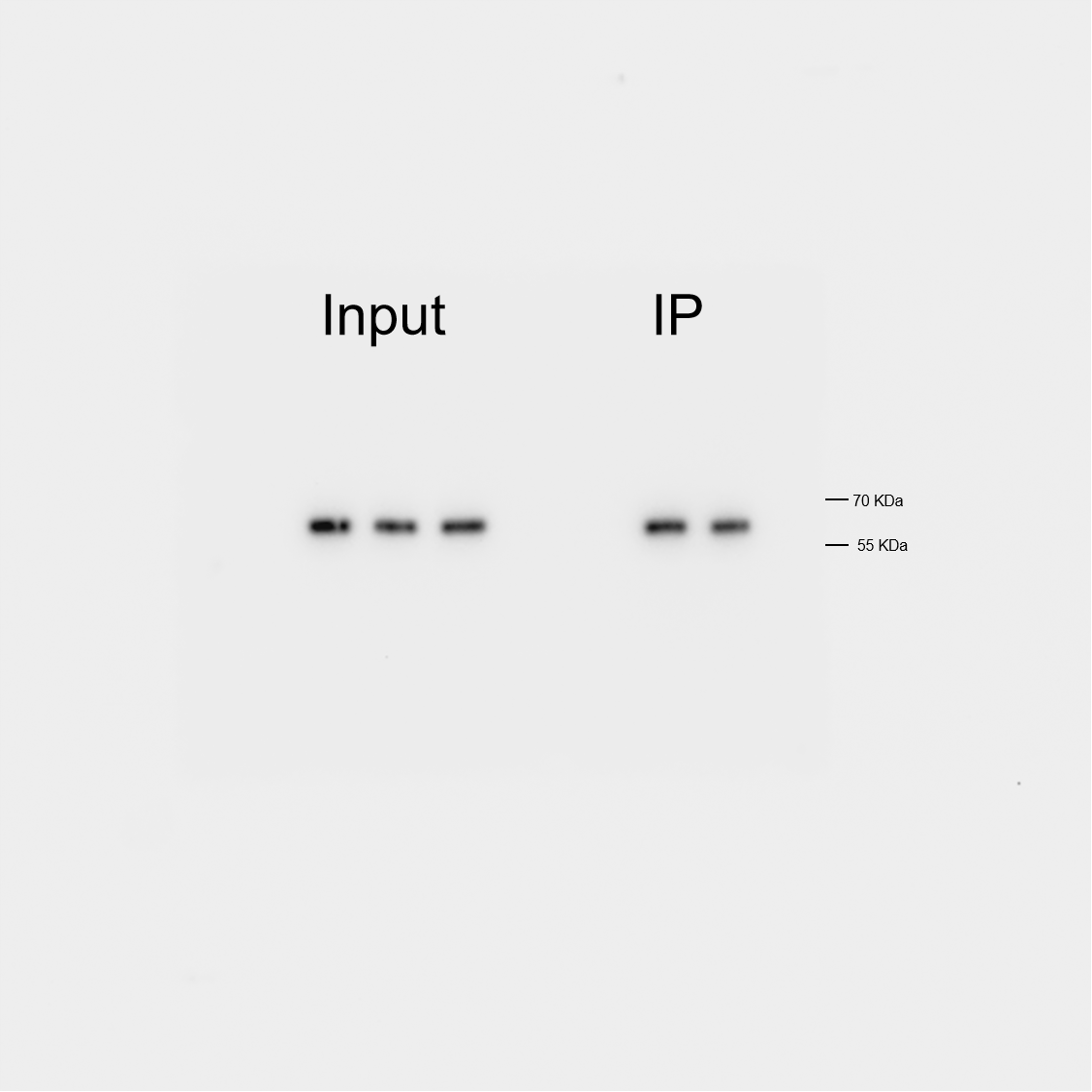

Supplement: Supplementary file 9 — Source data Fig. 3 [file 44319_2025_556_MOESM9_ESM.zip › Figure 3/3D/图片2.tif]

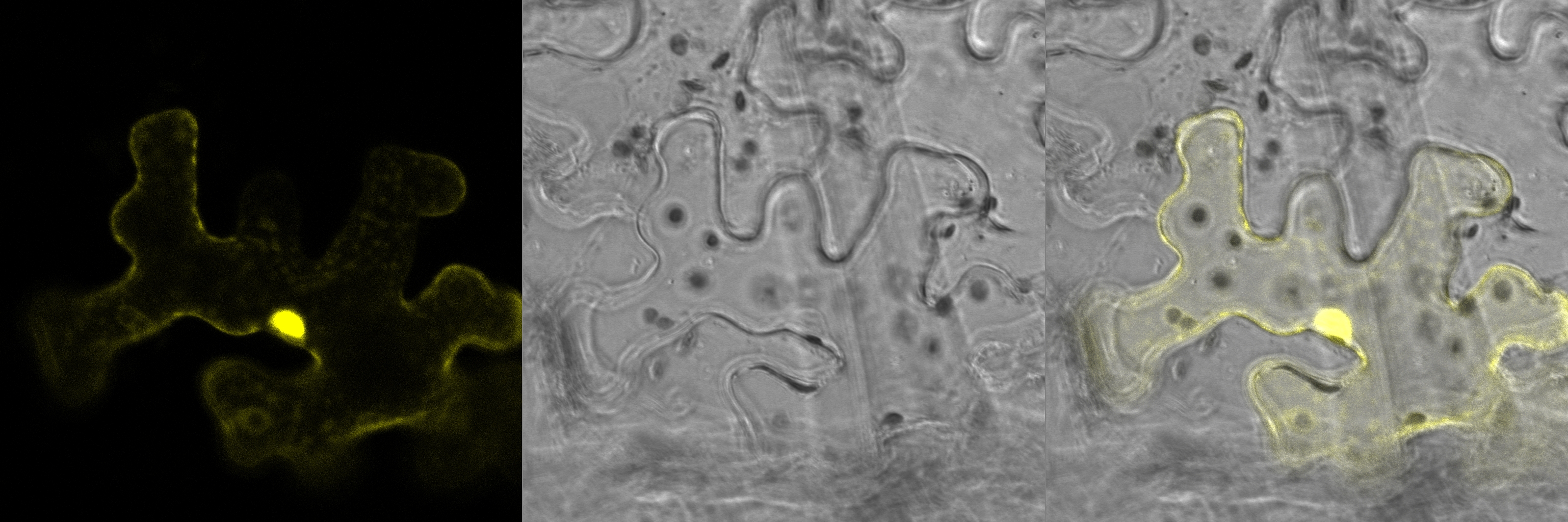

Supplement: Supplementary file 9 — Source data Fig. 3 [file 44319_2025_556_MOESM9_ESM.zip › Figure 3/3E/SRAS1.1+2A.jpg]

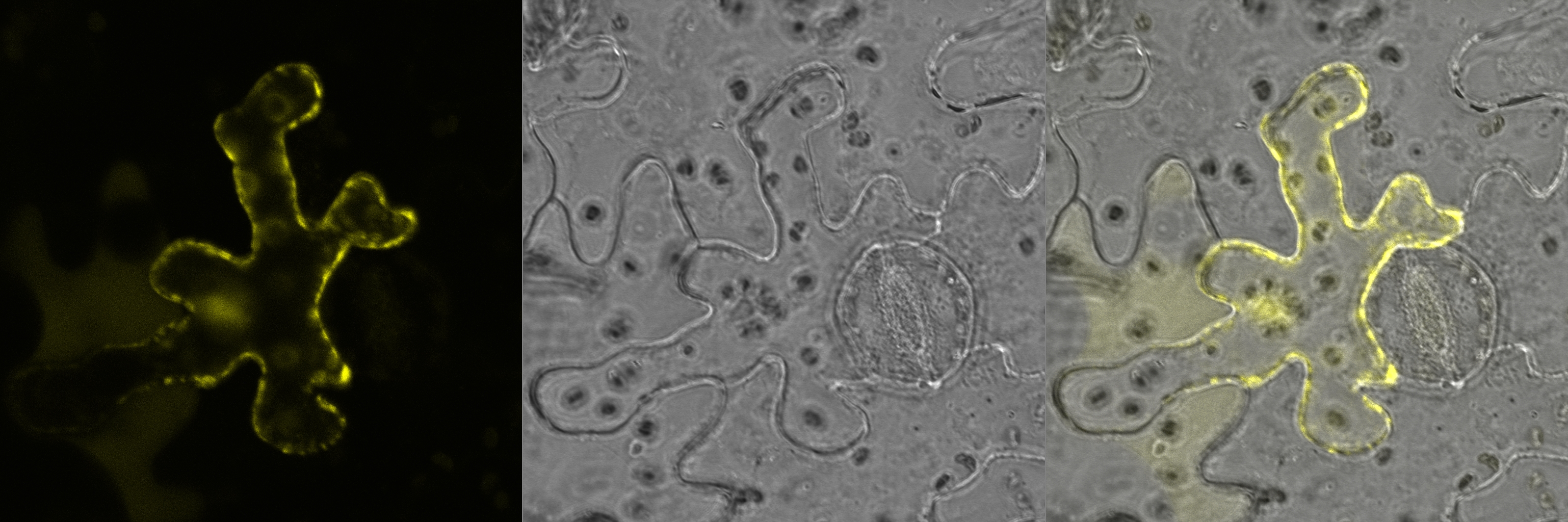

Supplement: Supplementary file 9 — Source data Fig. 3 [file 44319_2025_556_MOESM9_ESM.zip › Figure 3/3E/SRAS1.1+2B.jpg]

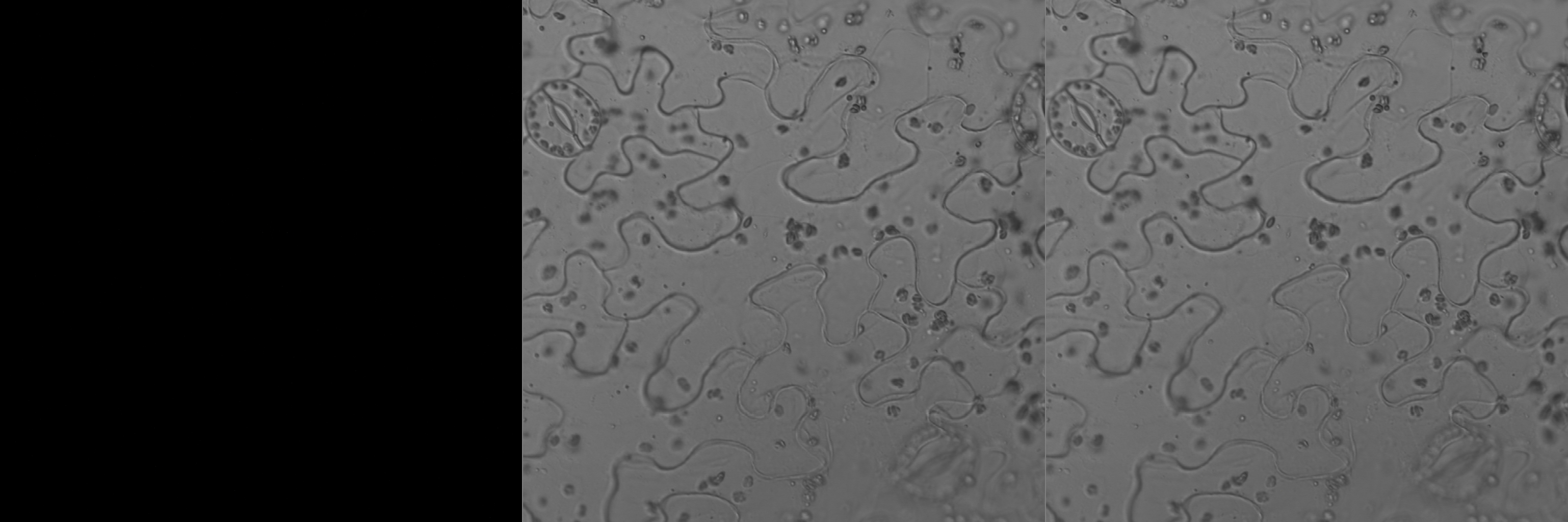

Supplement: Supplementary file 9 — Source data Fig. 3 [file 44319_2025_556_MOESM9_ESM.zip › Figure 3/3E/SRAS1.1+ABI5.jpg]

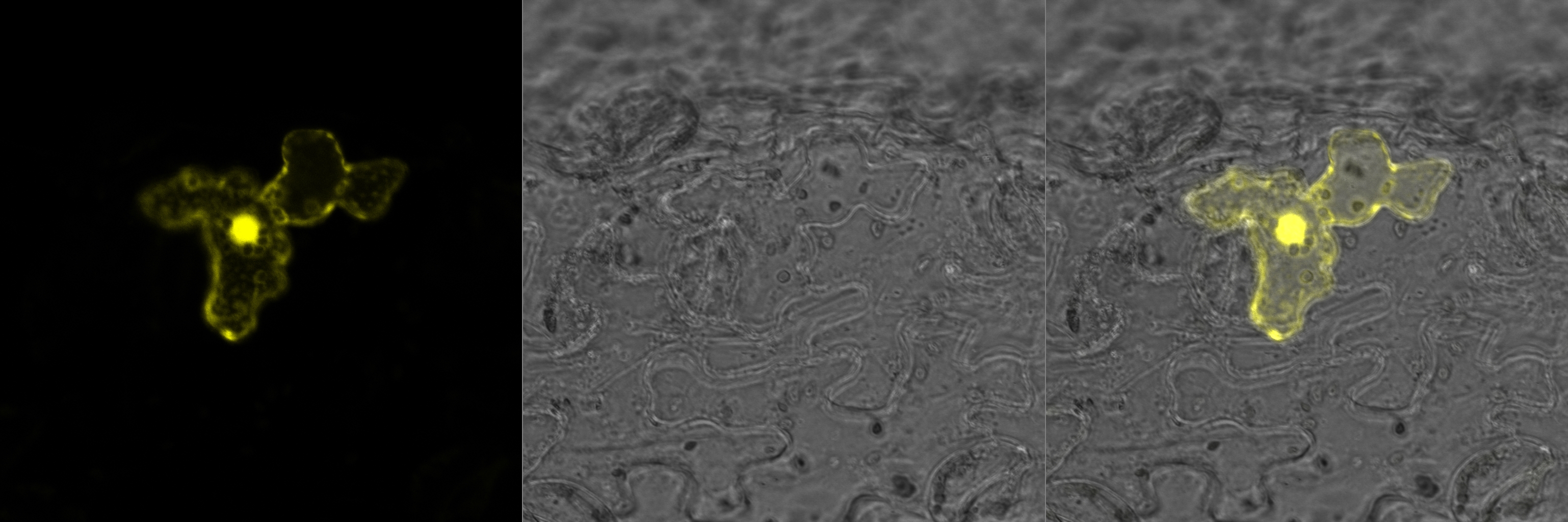

Supplement: Supplementary file 9 — Source data Fig. 3 [file 44319_2025_556_MOESM9_ESM.zip › Figure 3/3E/SRAS1.1+CSN5A.jpg]

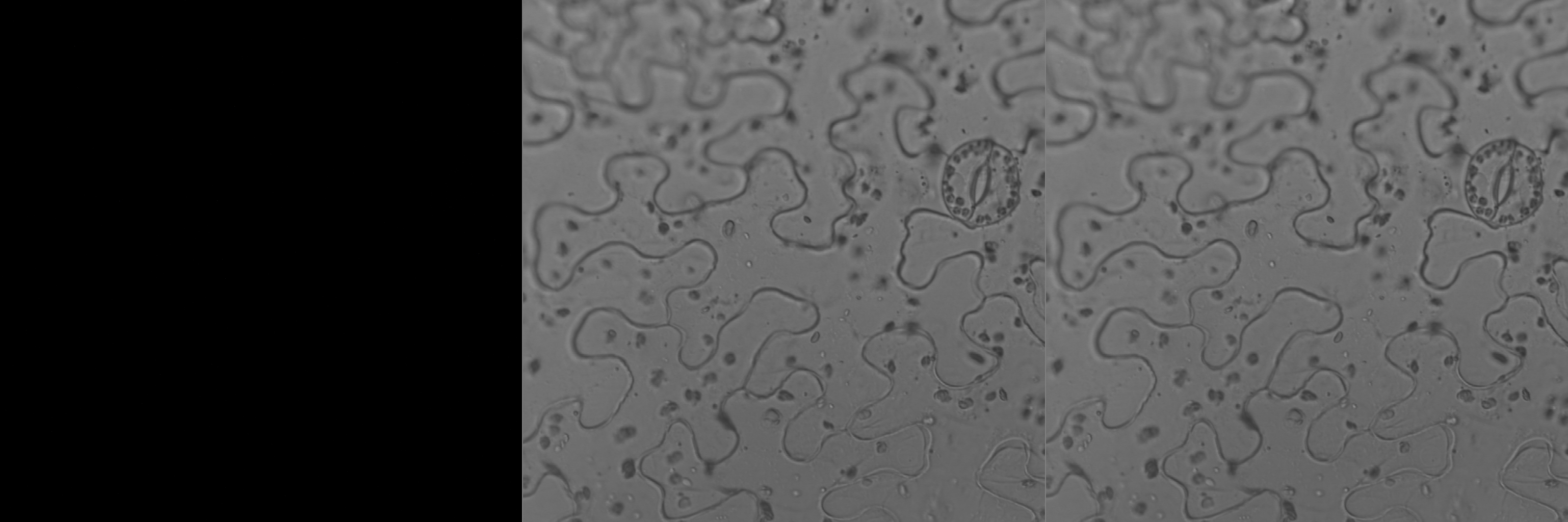

Supplement: Supplementary file 9 — Source data Fig. 3 [file 44319_2025_556_MOESM9_ESM.zip › Figure 3/3E/cYFP+DSK2A-nYFP.jpg]

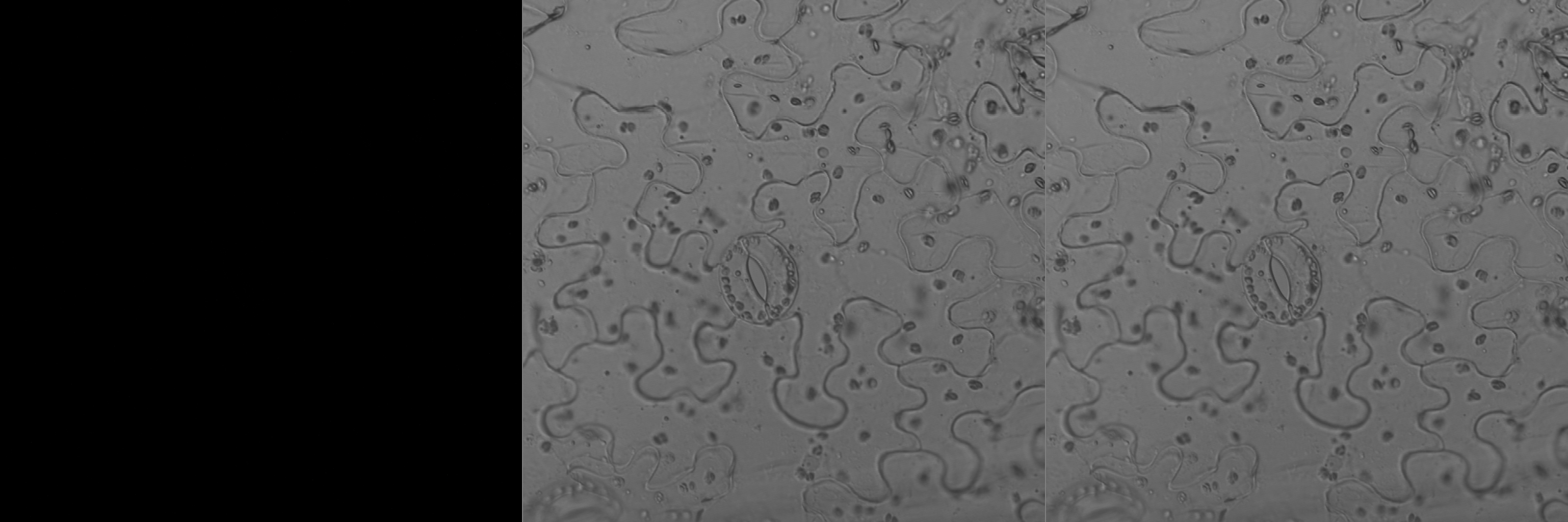

Supplement: Supplementary file 9 — Source data Fig. 3 [file 44319_2025_556_MOESM9_ESM.zip › Figure 3/3E/cYFP+DSK2B-nYFP.jpg]

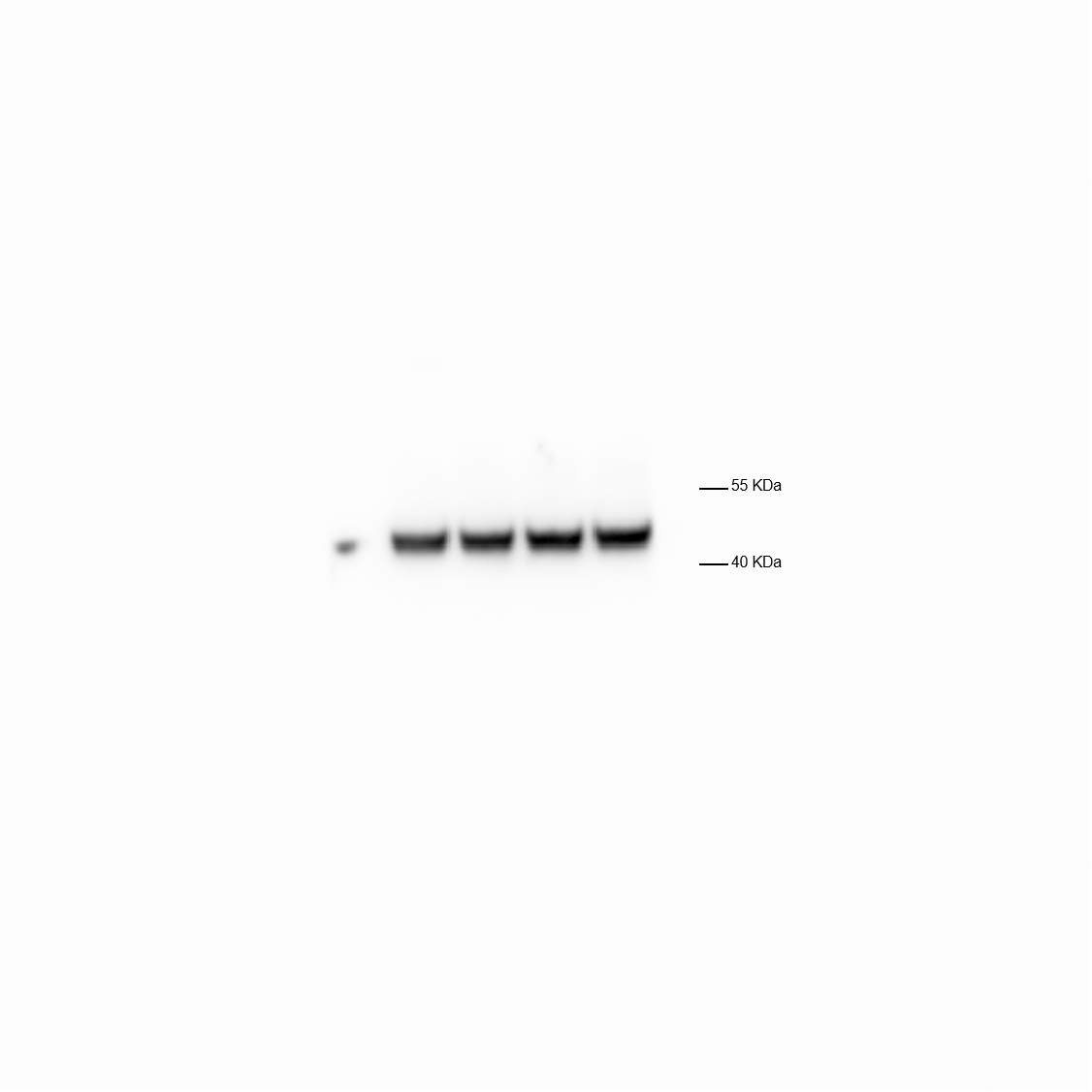

Supplement: Supplementary file 10 — Source data Fig. 4 [file 44319_2025_556_MOESM10_ESM.zip › Figure 4/4A/ACTIN.tif]

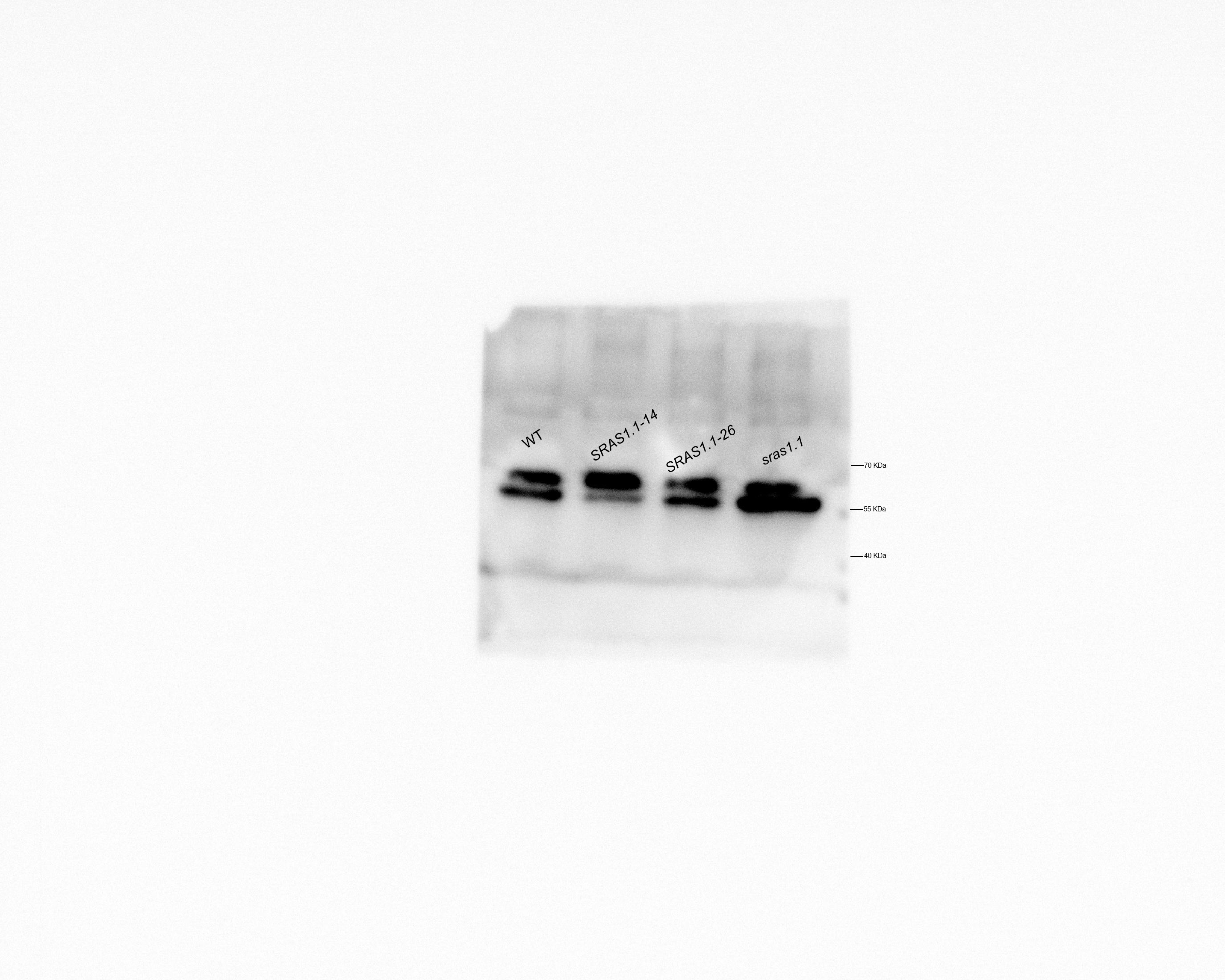

Supplement: Supplementary file 10 — Source data Fig. 4 [file 44319_2025_556_MOESM10_ESM.zip › Figure 4/4A/DSK2.tif]

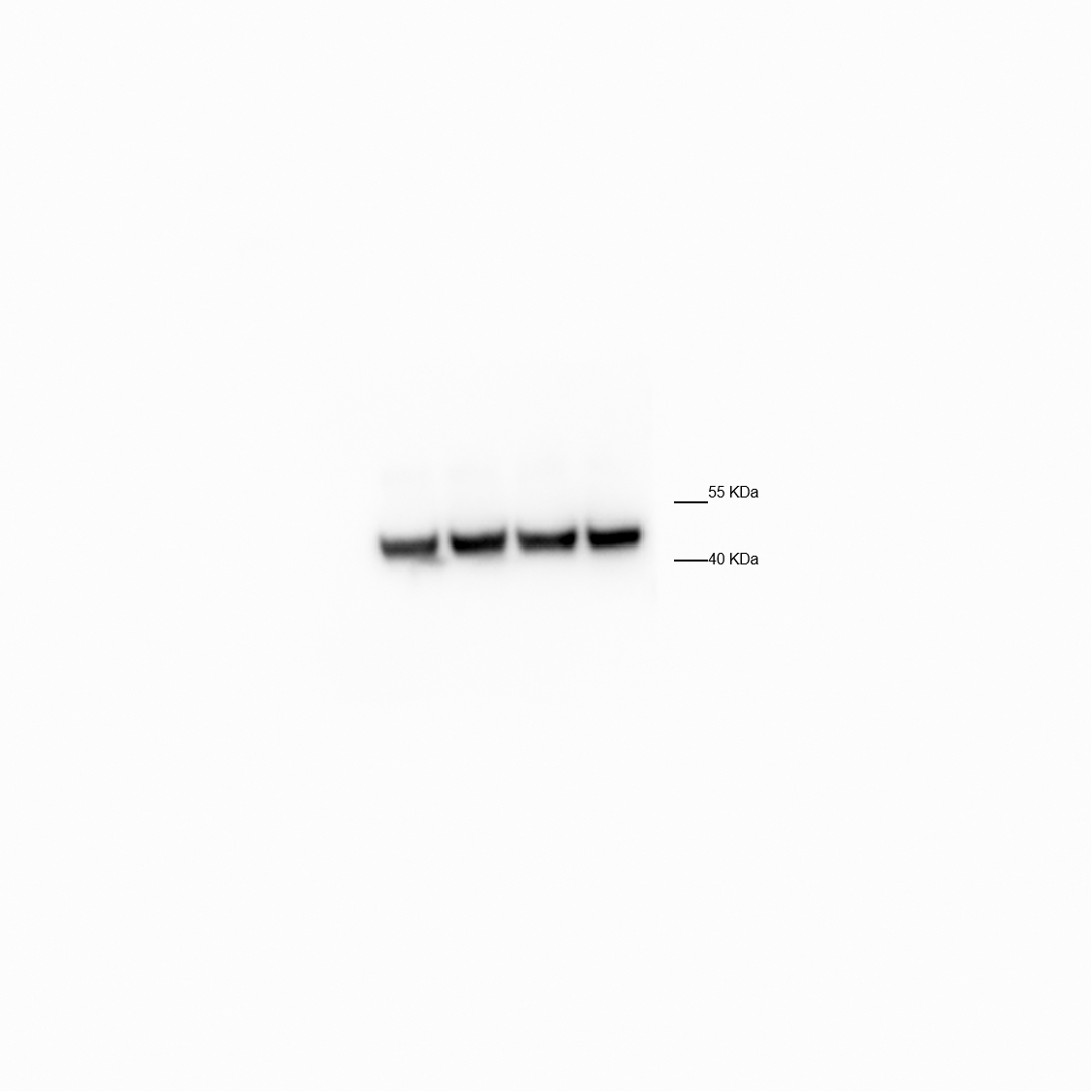

Supplement: Supplementary file 10 — Source data Fig. 4 [file 44319_2025_556_MOESM10_ESM.zip › Figure 4/4B/SRAS1.1-GST.tif]

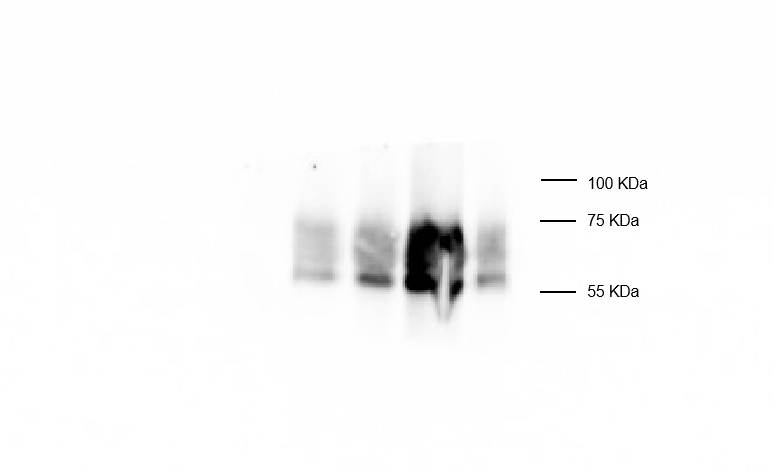

Supplement: Supplementary file 10 — Source data Fig. 4 [file 44319_2025_556_MOESM10_ESM.zip › Figure 4/4B/ubqitin.tif]

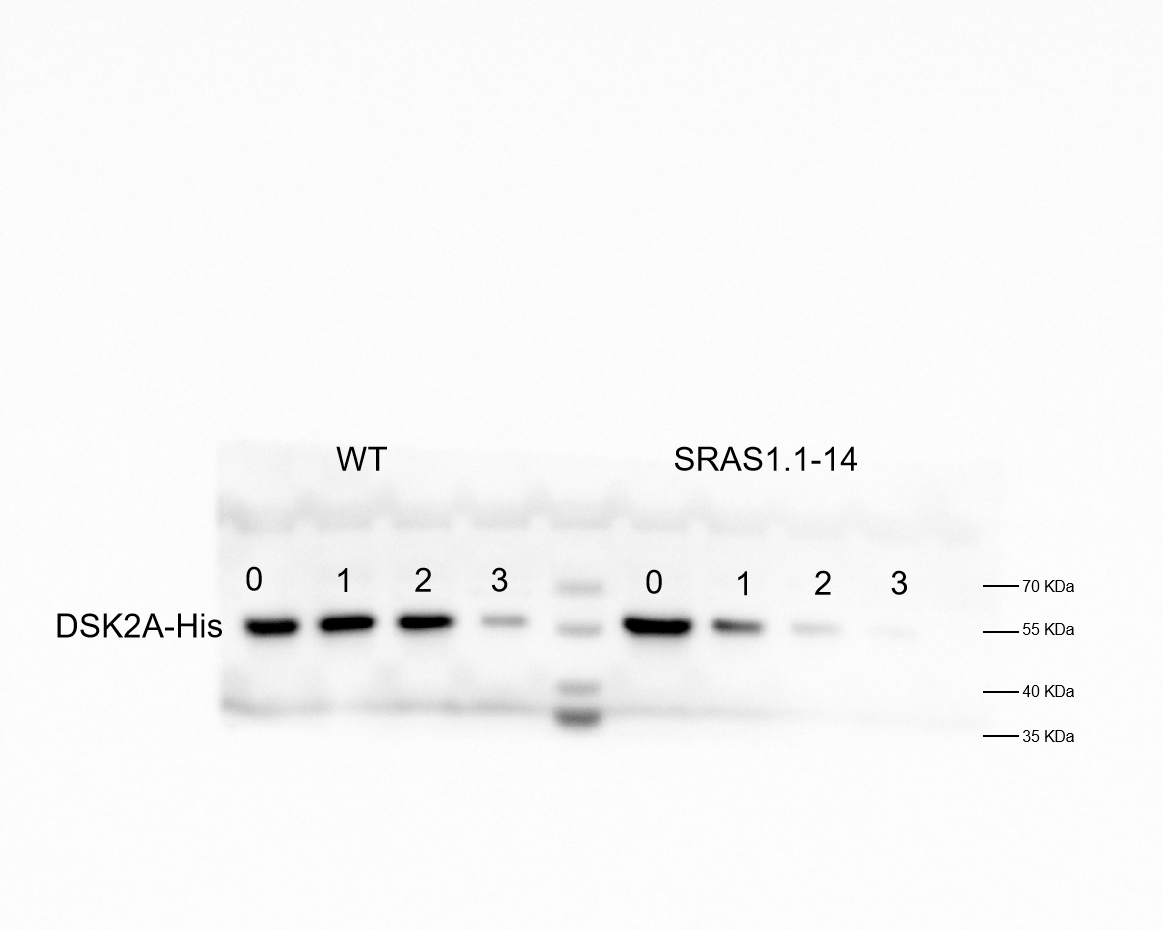

Supplement: Supplementary file 10 — Source data Fig. 4 [file 44319_2025_556_MOESM10_ESM.zip › Figure 4/4C/DSK2A-His.tif]

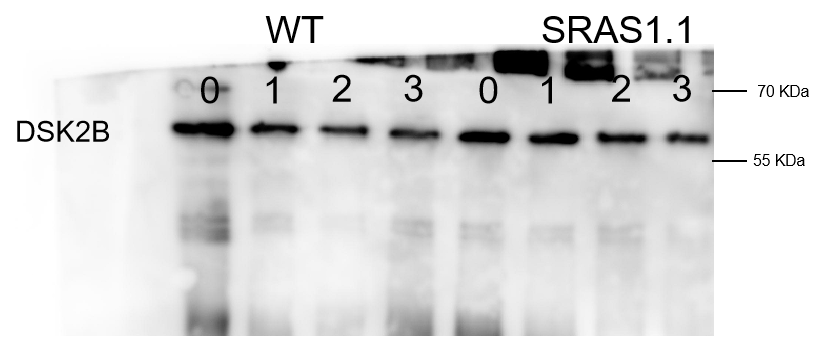

Supplement: Supplementary file 10 — Source data Fig. 4 [file 44319_2025_556_MOESM10_ESM.zip › Figure 4/4D/图片1.tif]

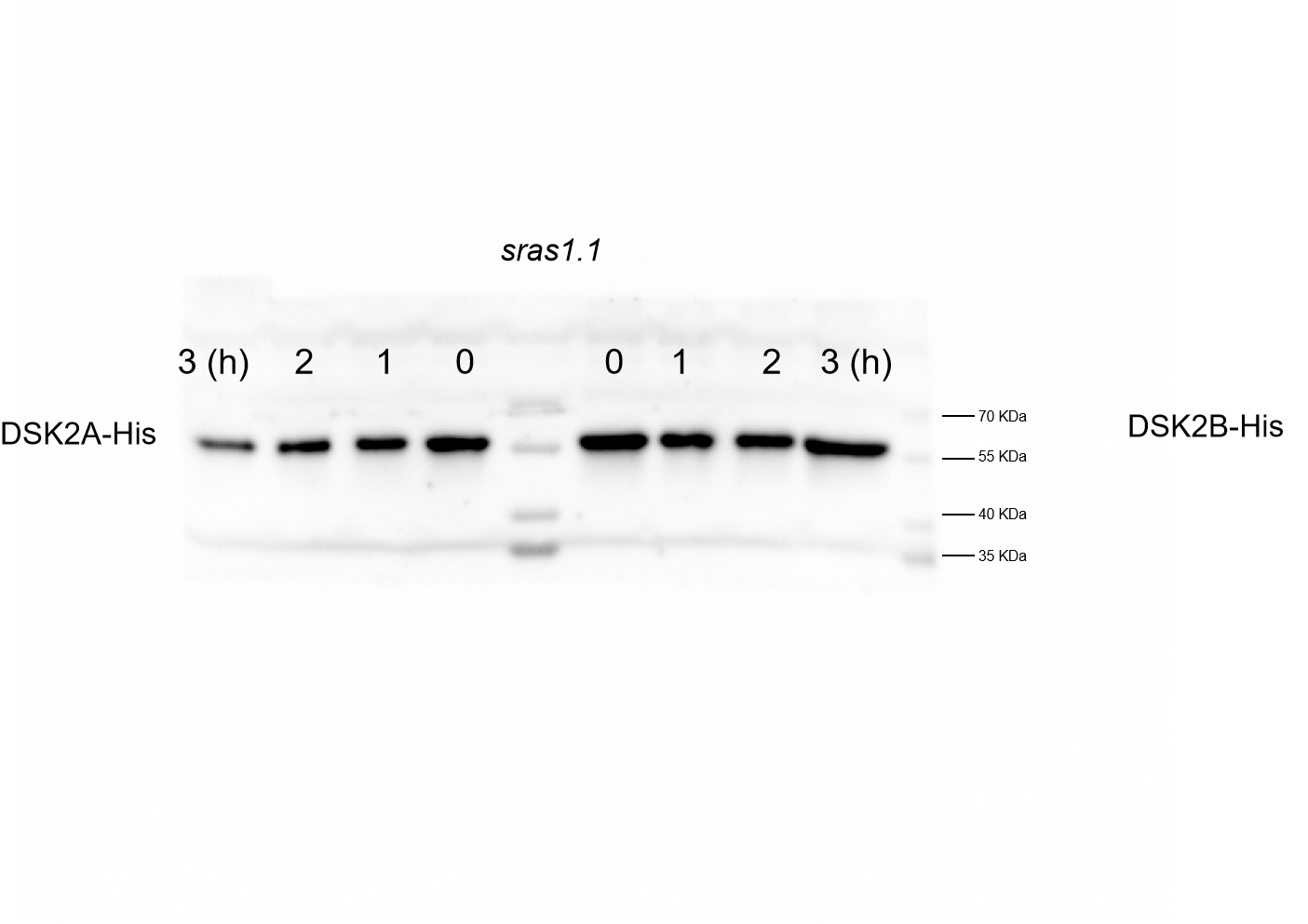

Supplement: Supplementary file 10 — Source data Fig. 4 [file 44319_2025_556_MOESM10_ESM.zip › Figure 4/4D/图片4.tif]

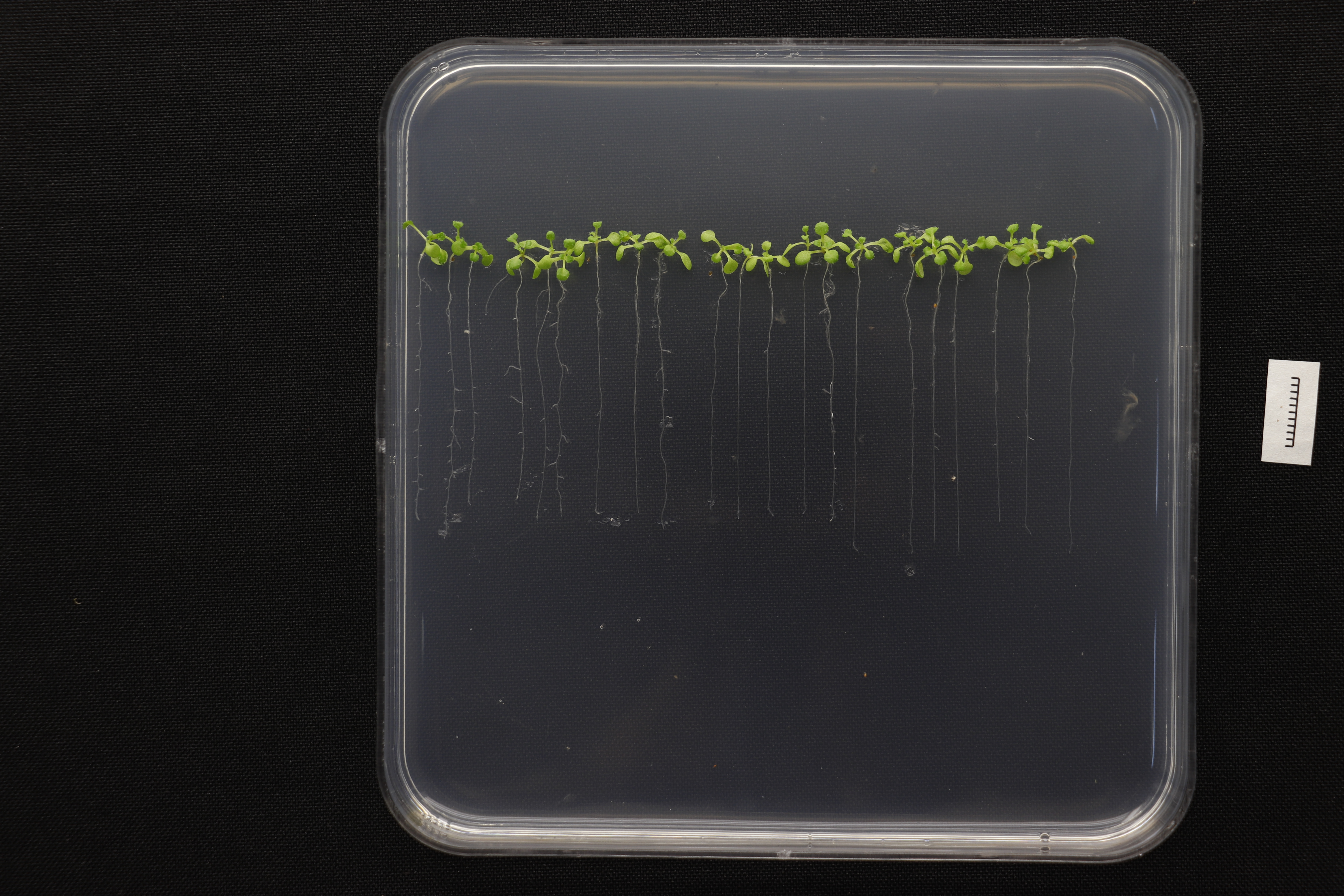

Supplement: Supplementary file 11 — Source data Fig. 5 [file 44319_2025_556_MOESM11_ESM.zip › Figure 5/5A/12MS.tif]

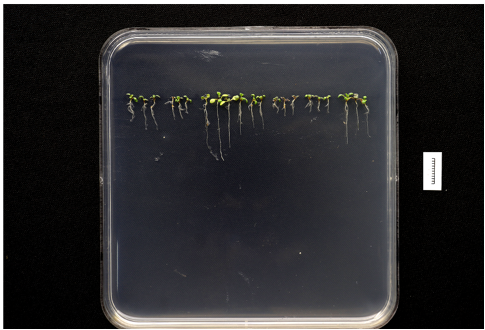

Supplement: Supplementary file 11 — Source data Fig. 5 [file 44319_2025_556_MOESM11_ESM.zip › Figure 5/5A/Drought stress.tif]

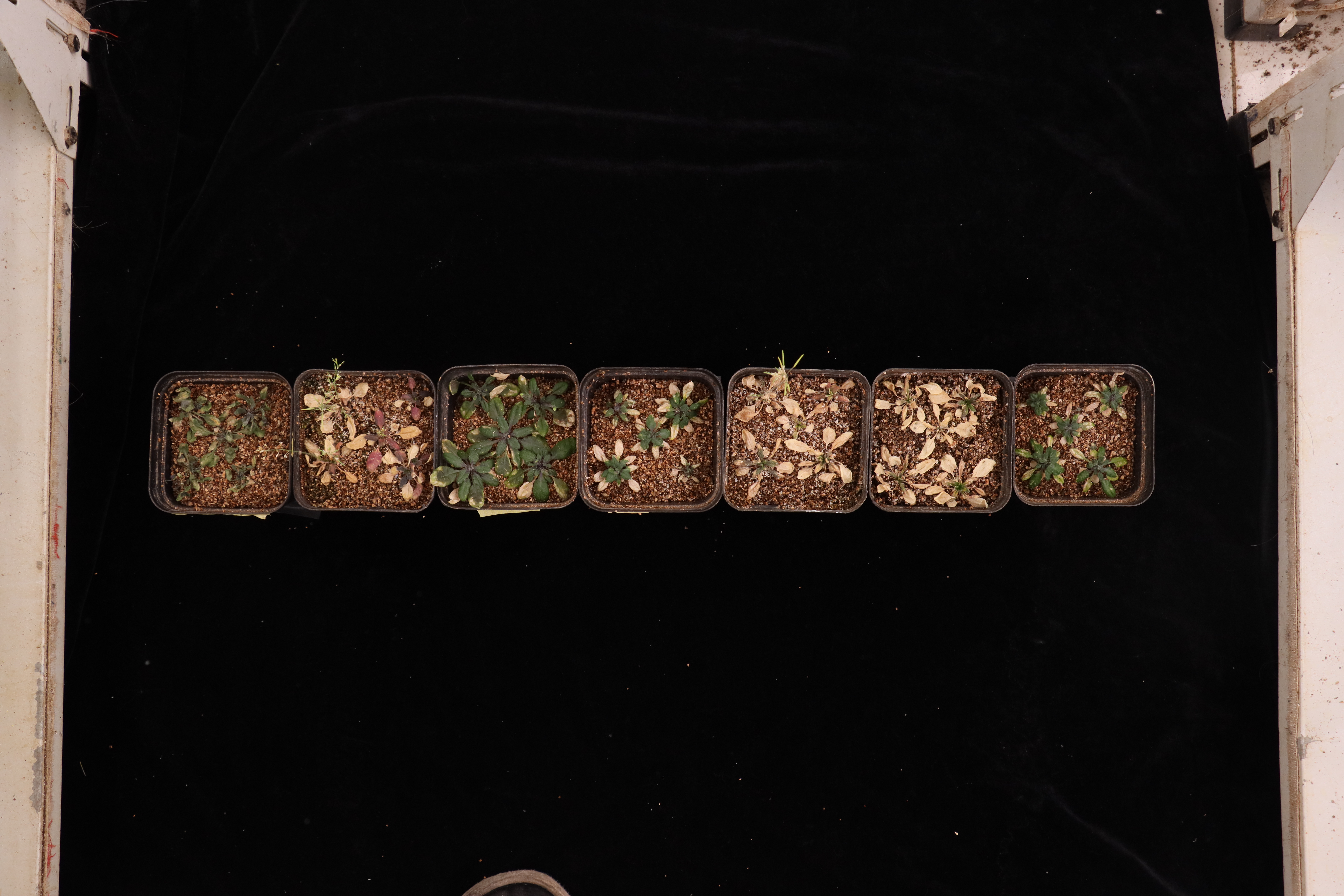

Supplement: Supplementary file 11 — Source data Fig. 5 [file 44319_2025_556_MOESM11_ESM.zip › Figure 5/5D/Drought stress.JPG]

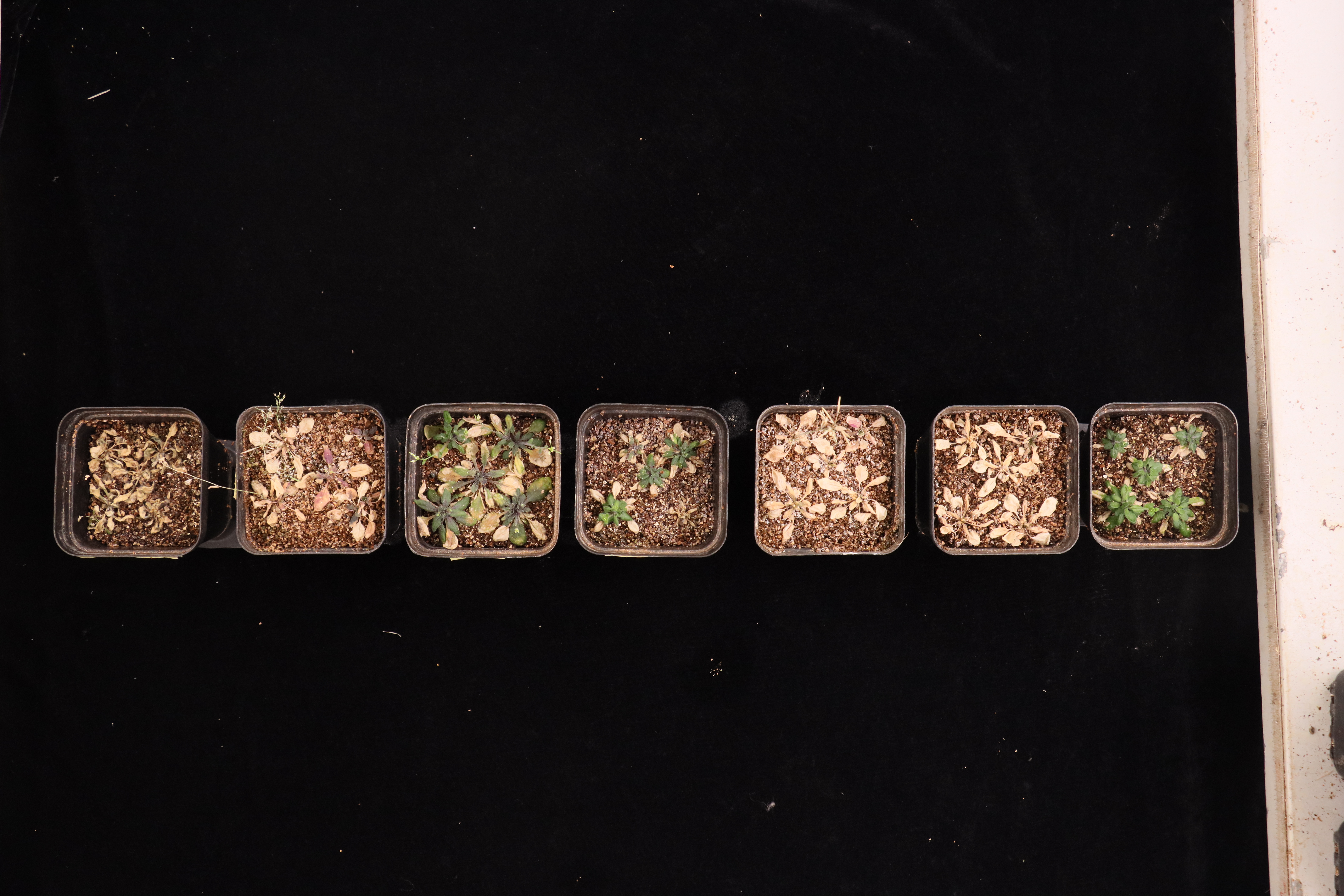

Supplement: Supplementary file 11 — Source data Fig. 5 [file 44319_2025_556_MOESM11_ESM.zip › Figure 5/5D/Re-water.JPG]

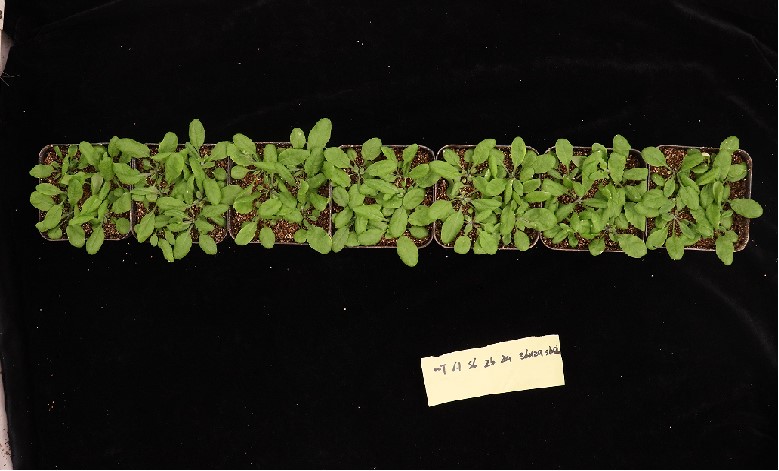

Supplement: Supplementary file 11 — Source data Fig. 5 [file 44319_2025_556_MOESM11_ESM.zip › Figure 5/5D/ck.jpg]

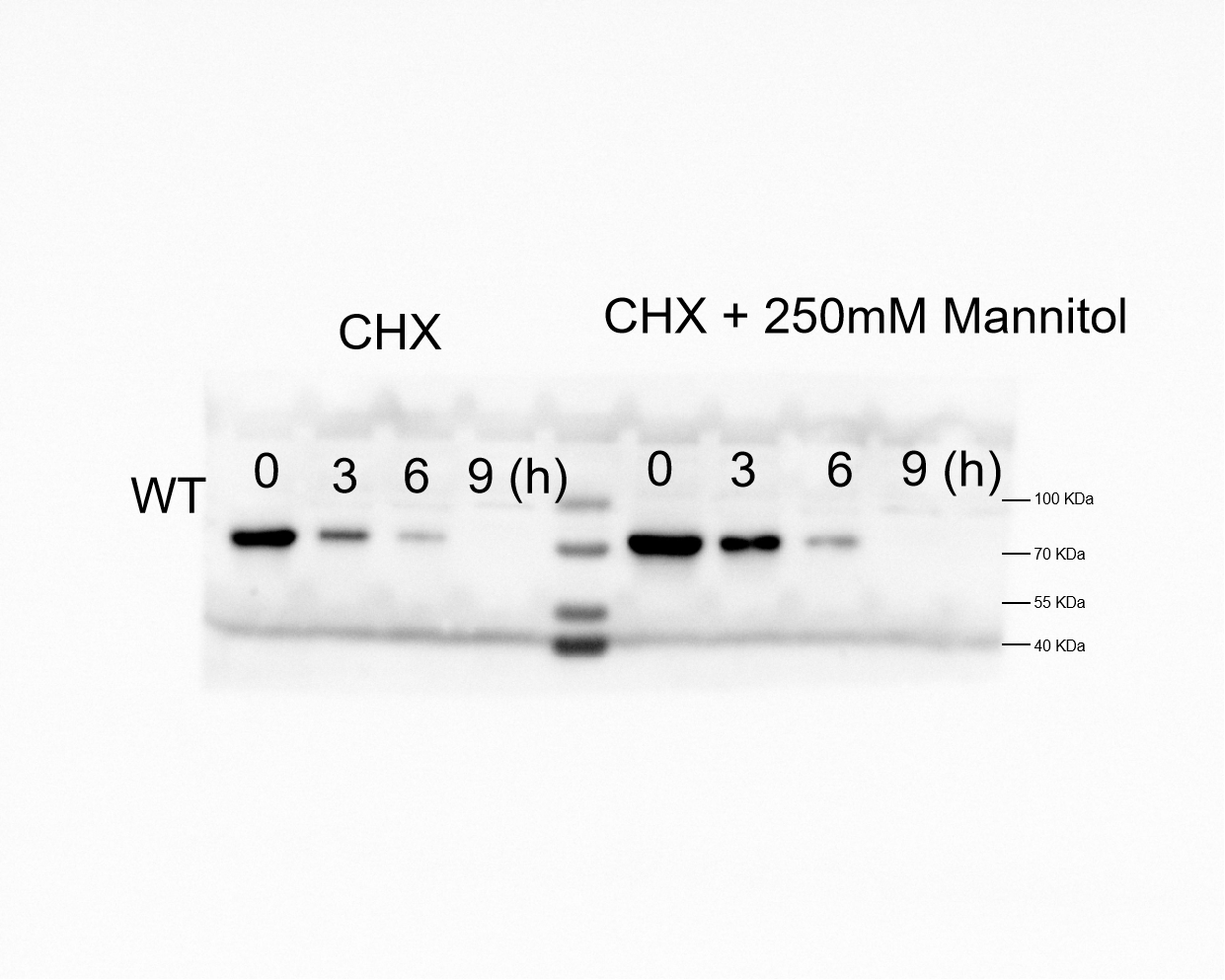

Supplement: Supplementary file 11 — Source data Fig. 5 [file 44319_2025_556_MOESM11_ESM.zip › Figure 5/5G/1.tif]

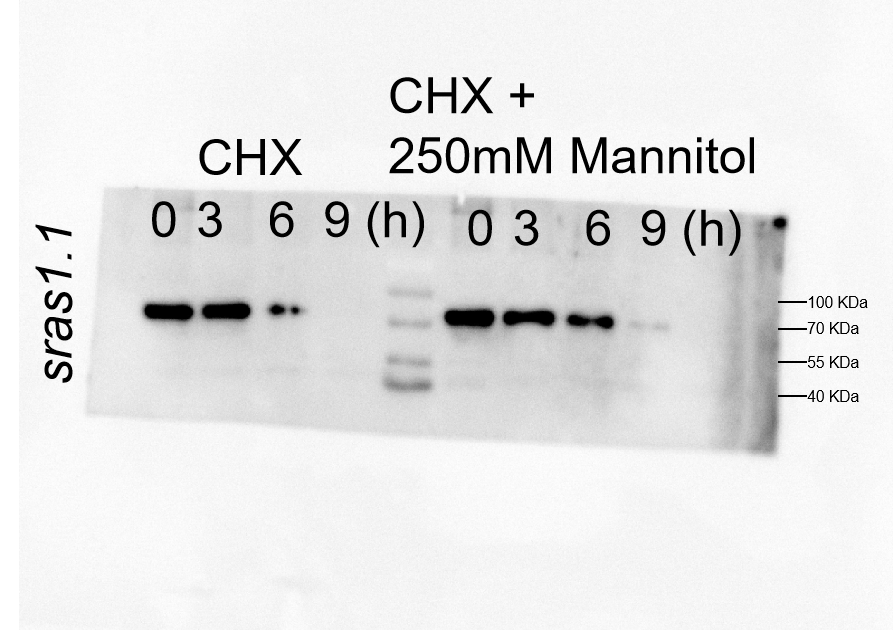

Supplement: Supplementary file 11 — Source data Fig. 5 [file 44319_2025_556_MOESM11_ESM.zip › Figure 5/5G/图片2.tif]

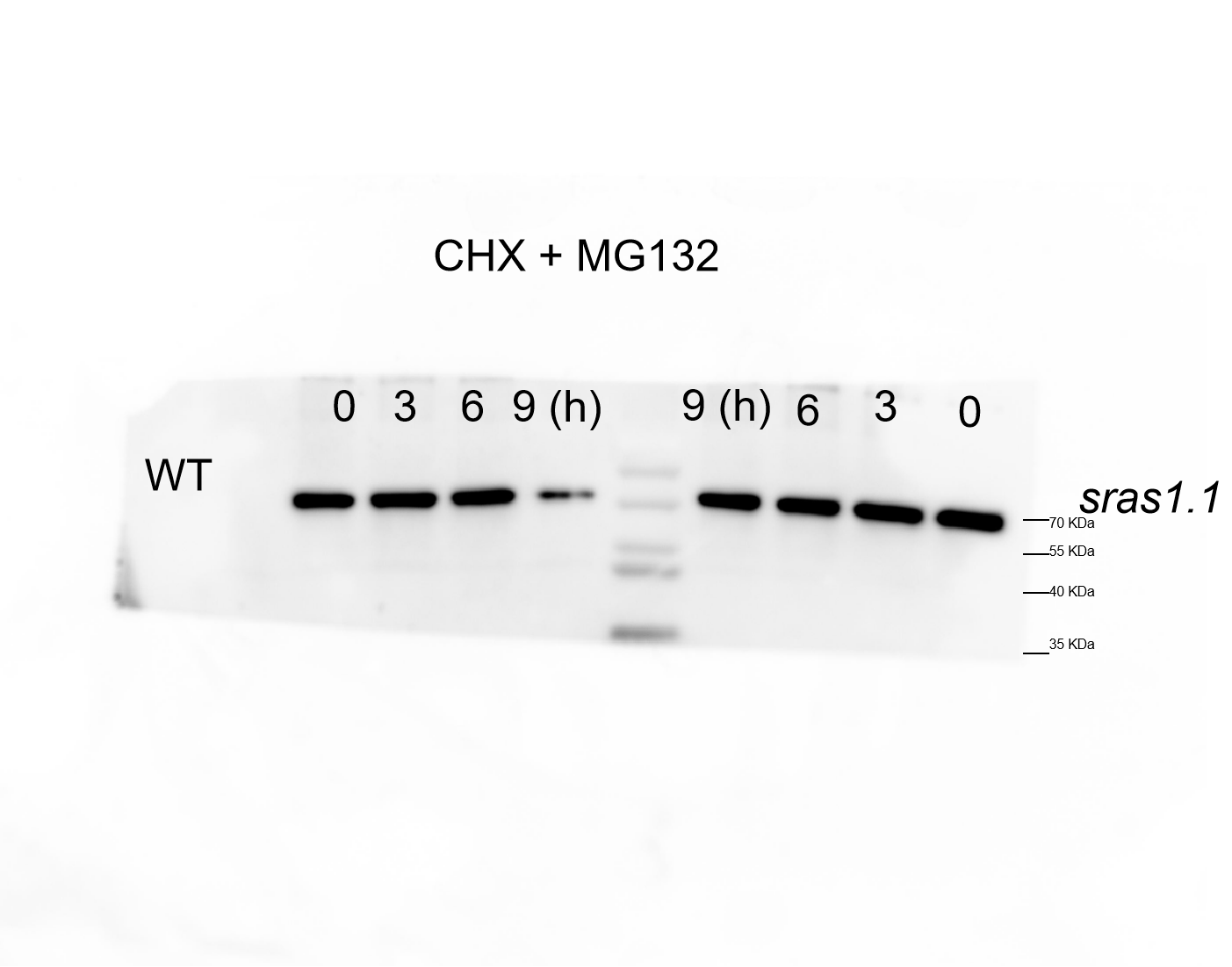

Supplement: Supplementary file 11 — Source data Fig. 5 [file 44319_2025_556_MOESM11_ESM.zip › Figure 5/5G/图片3.tif]

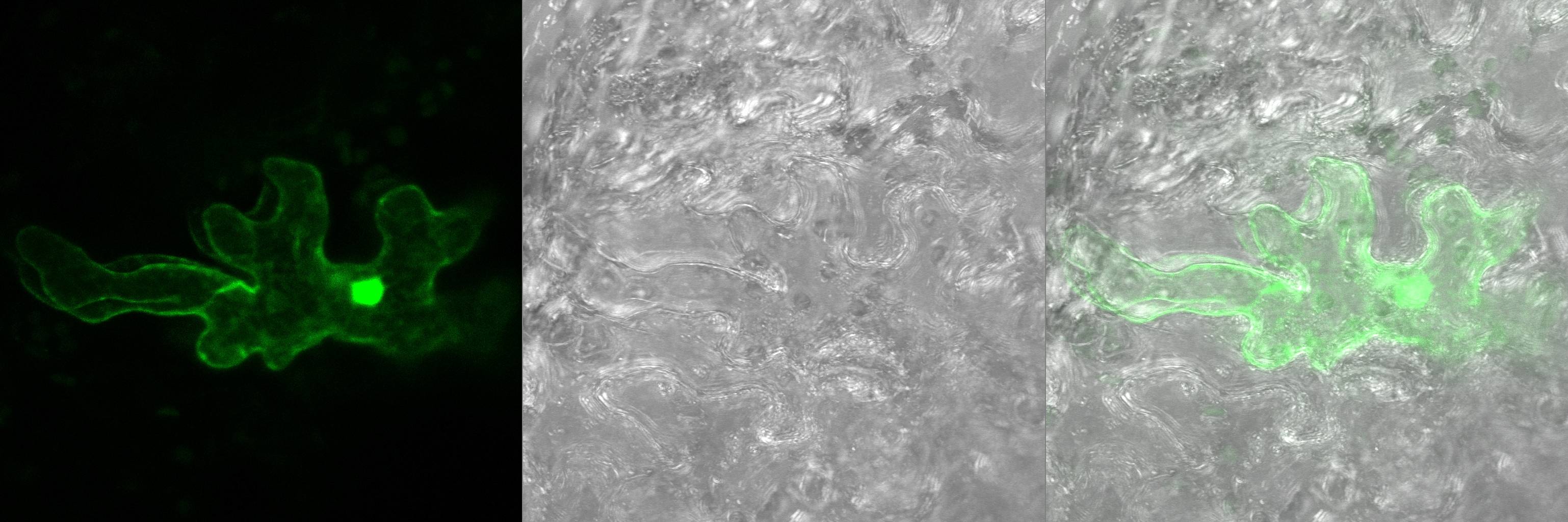

Supplement: Supplementary file 12 — Source data Fig. 6 [file 44319_2025_556_MOESM12_ESM.zip › Figure 6/6A/Man+ConA.jpg]

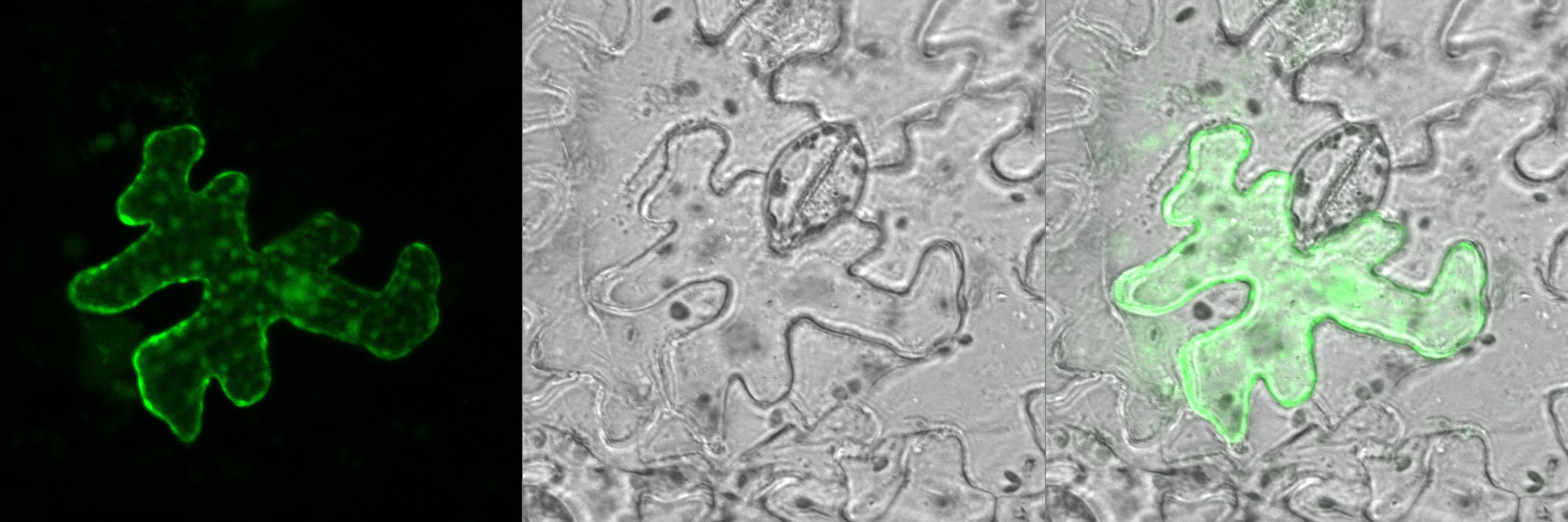

Supplement: Supplementary file 12 — Source data Fig. 6 [file 44319_2025_556_MOESM12_ESM.zip › Figure 6/6A/Man.jpg]

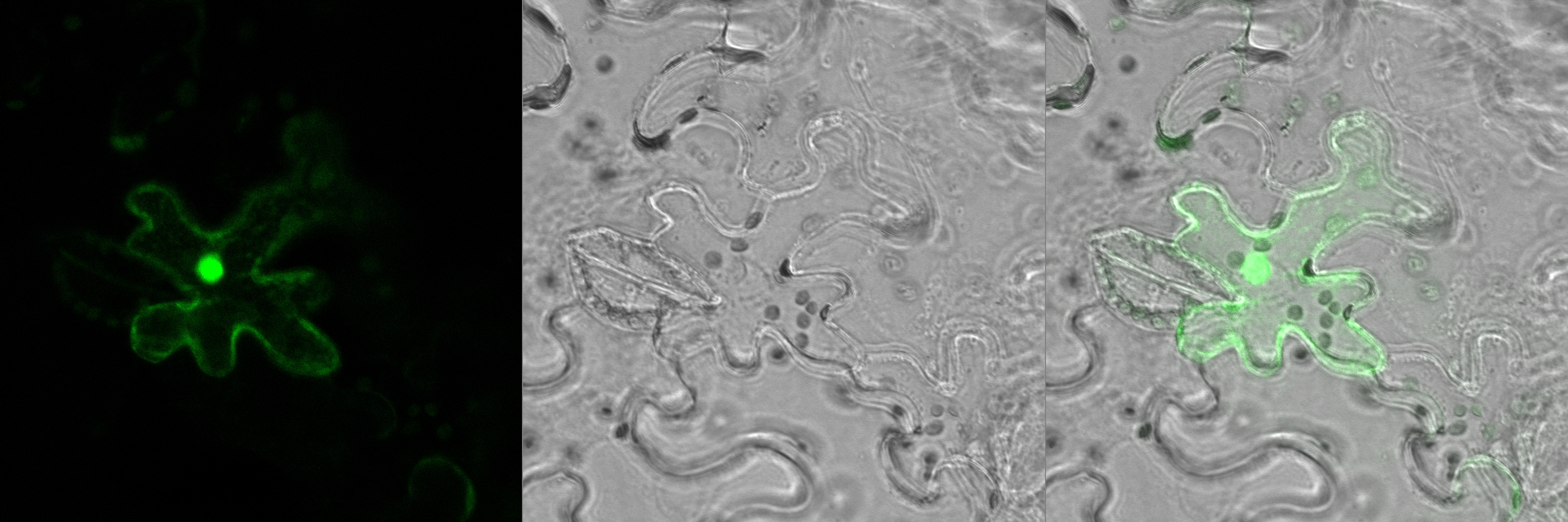

Supplement: Supplementary file 12 — Source data Fig. 6 [file 44319_2025_556_MOESM12_ESM.zip › Figure 6/6A/Mock.jpg]

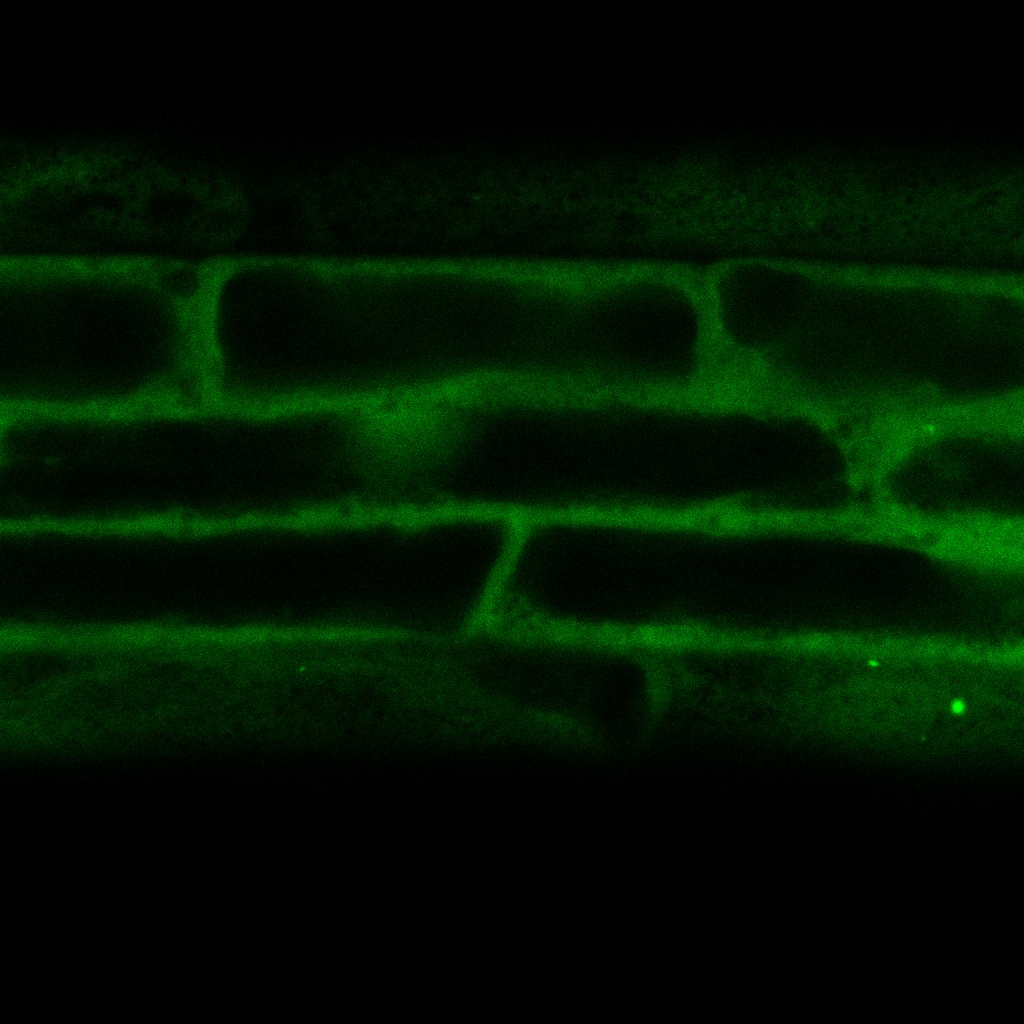

Supplement: Supplementary file 12 — Source data Fig. 6 [file 44319_2025_556_MOESM12_ESM.zip › Figure 6/6B/MOCK-WT.jpg]

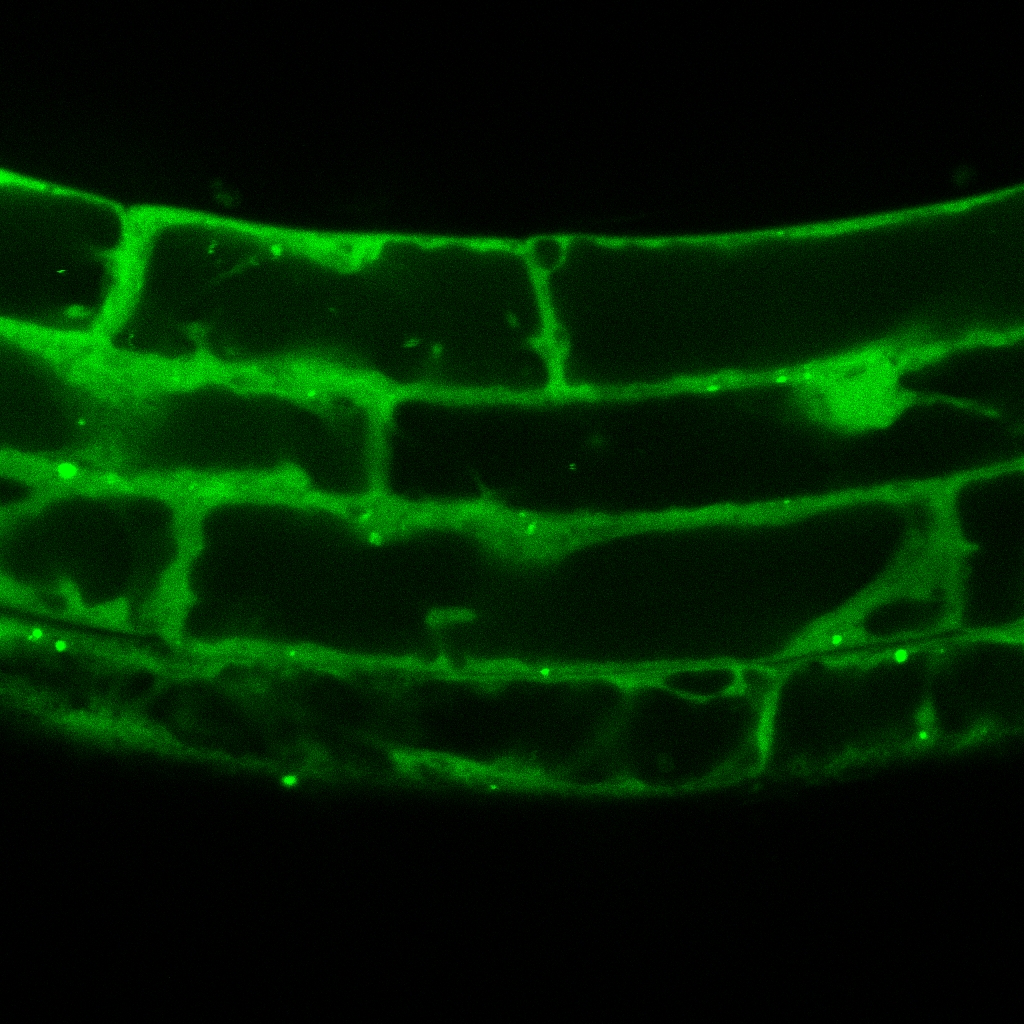

Supplement: Supplementary file 12 — Source data Fig. 6 [file 44319_2025_556_MOESM12_ESM.zip › Figure 6/6B/MOCK-sras1.1.jpg]

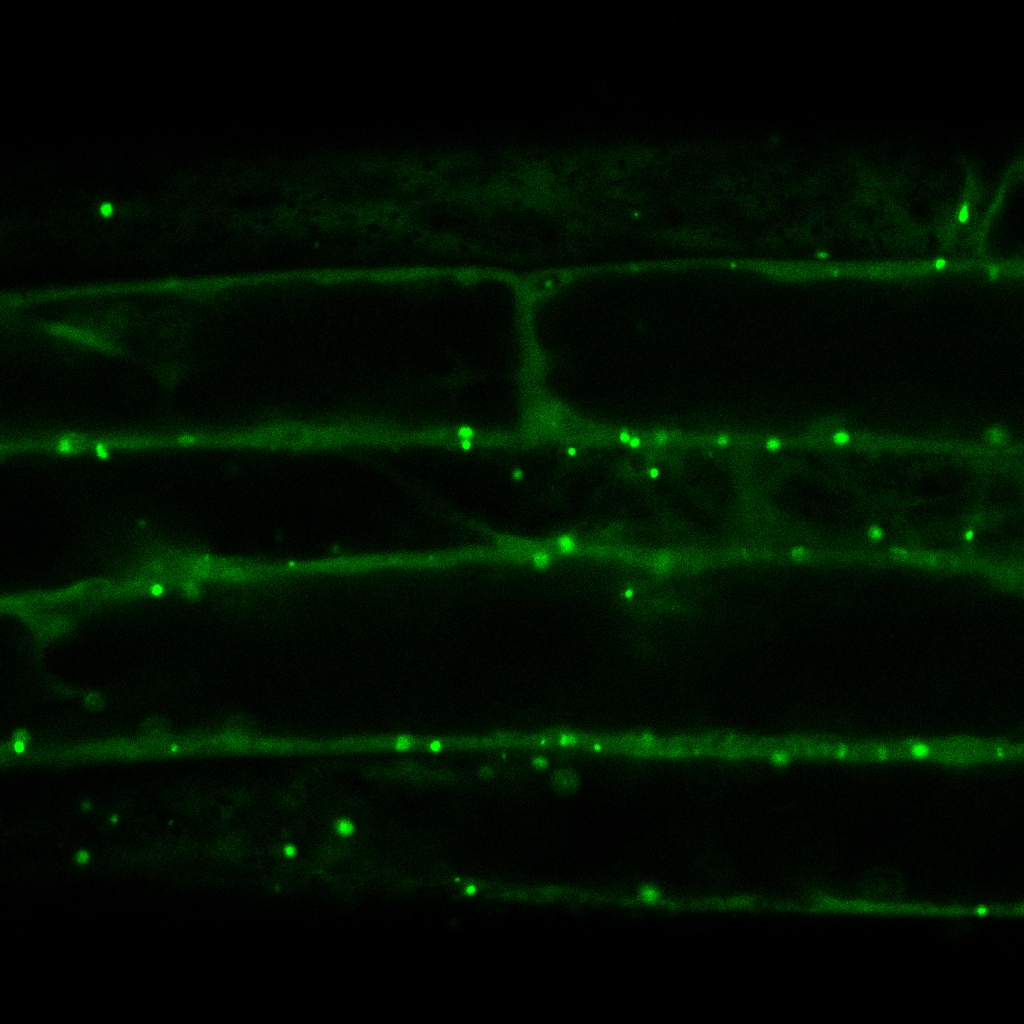

Supplement: Supplementary file 12 — Source data Fig. 6 [file 44319_2025_556_MOESM12_ESM.zip › Figure 6/6B/Man10min-WT.jpg]

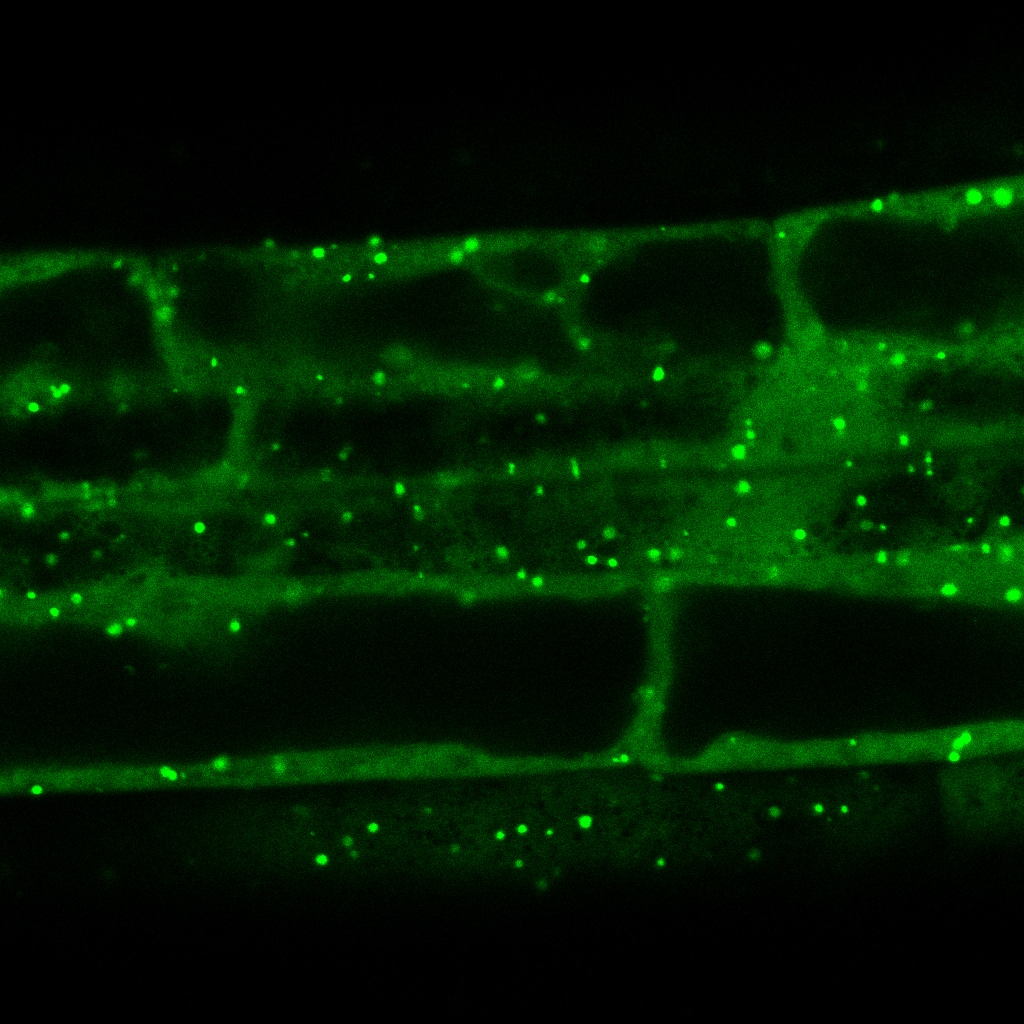

Supplement: Supplementary file 12 — Source data Fig. 6 [file 44319_2025_556_MOESM12_ESM.zip › Figure 6/6B/Man10min-sras1.1.jpg]

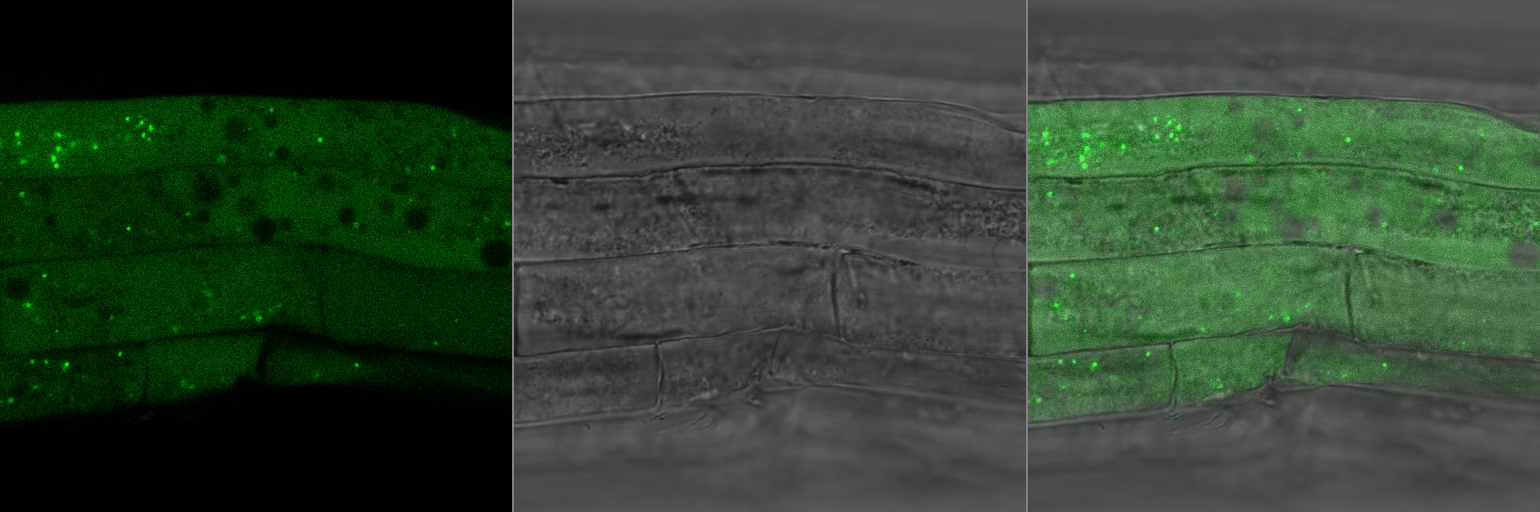

Supplement: Supplementary file 12 — Source data Fig. 6 [file 44319_2025_556_MOESM12_ESM.zip › Figure 6/6B/Man30min-WT.jpg]

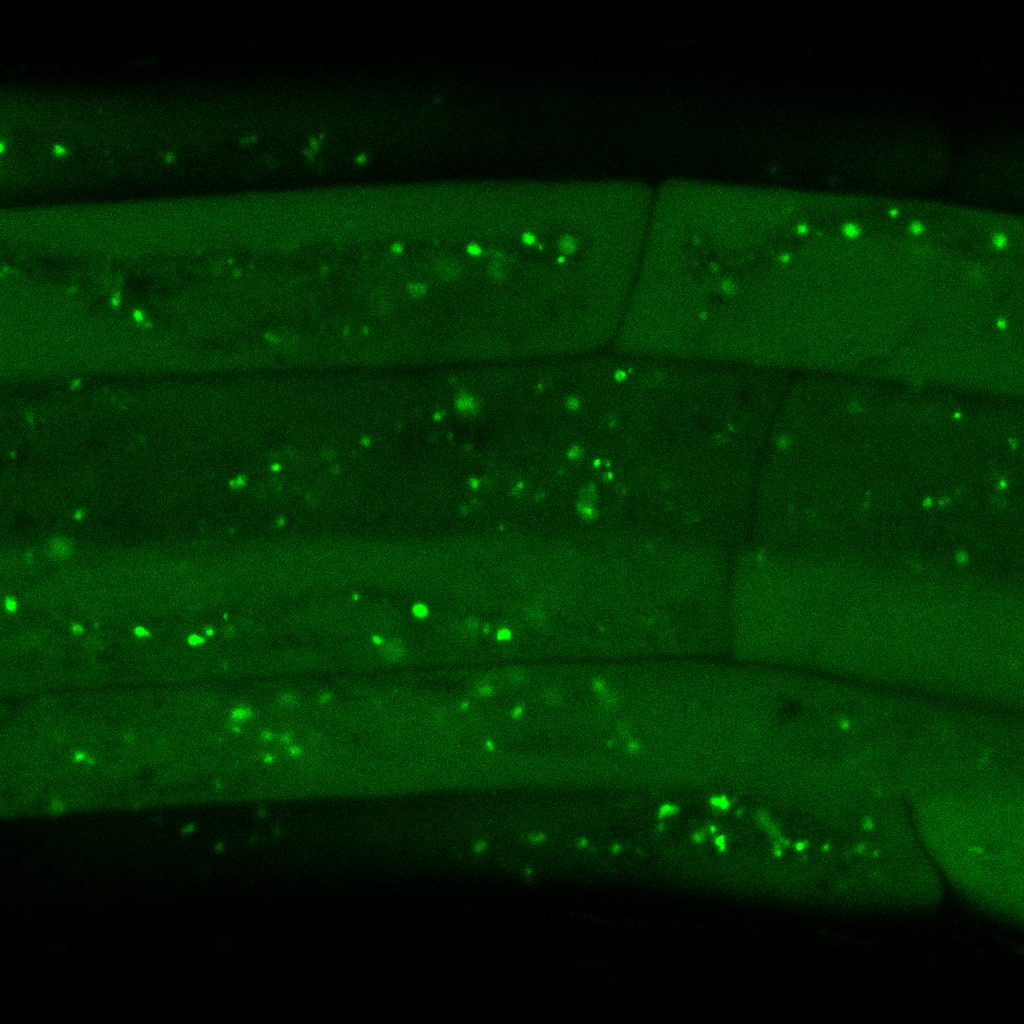

Supplement: Supplementary file 12 — Source data Fig. 6 [file 44319_2025_556_MOESM12_ESM.zip › Figure 6/6B/Man30min-sras1.1.jpg]

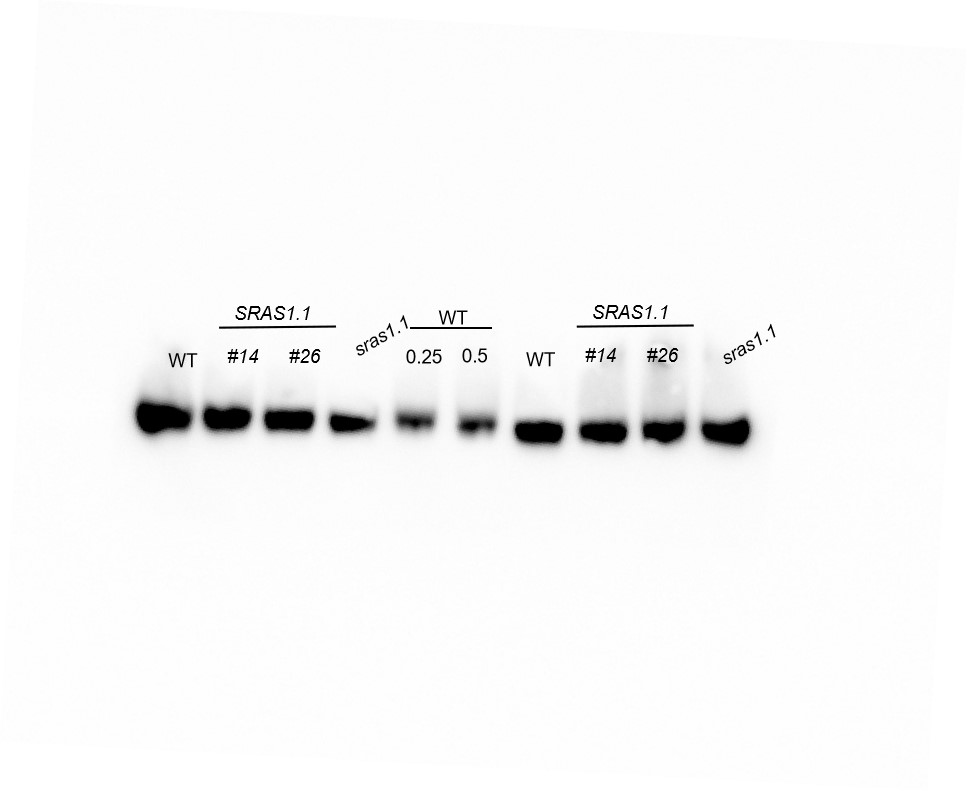

Supplement: Supplementary file 12 — Source data Fig. 6 [file 44319_2025_556_MOESM12_ESM.zip › Figure 6/6F/ACTIN.jpg]

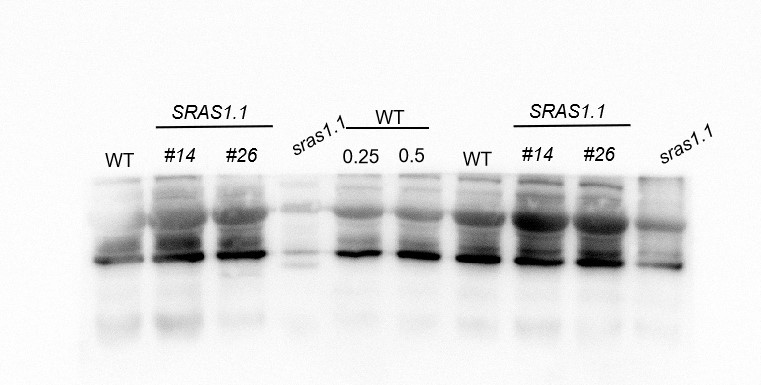

Supplement: Supplementary file 12 — Source data Fig. 6 [file 44319_2025_556_MOESM12_ESM.zip › Figure 6/6F/BES1.jpg]

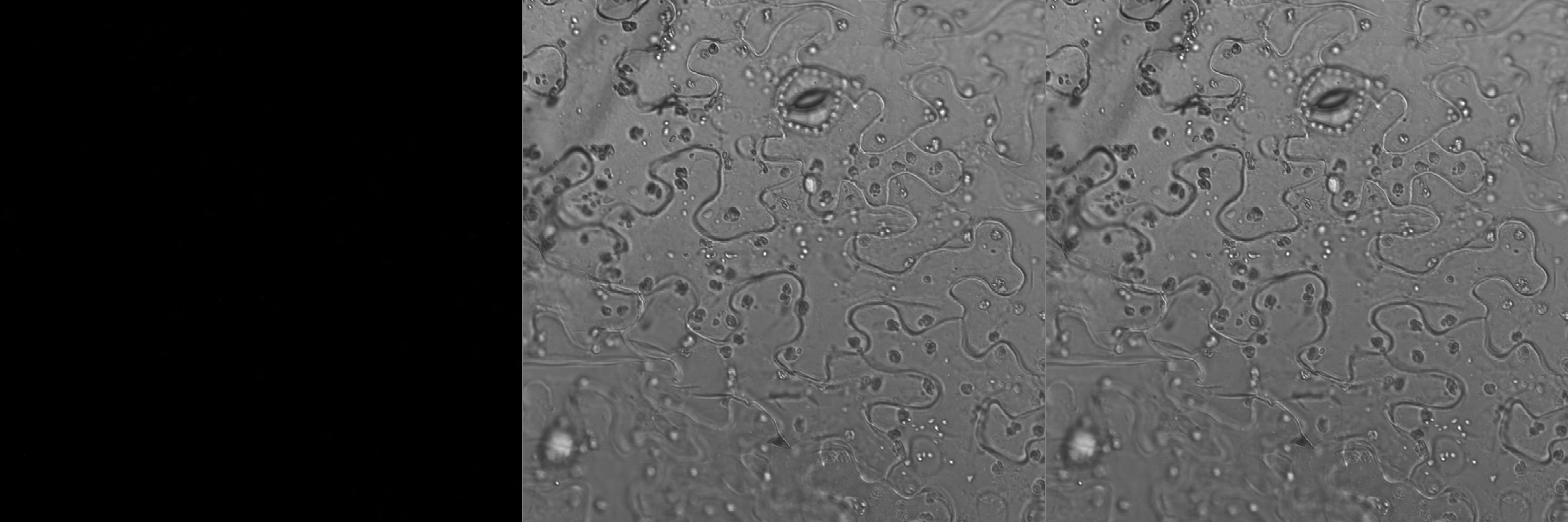

Supplement: Supplementary file 13 — Appendix Figure S8 Source Data [file 44319_2025_556_MOESM13_ESM.zip › Appendix Figure S8/S8A/SRAS1.2-cYFP+nYFP-DSK2A.jpg]

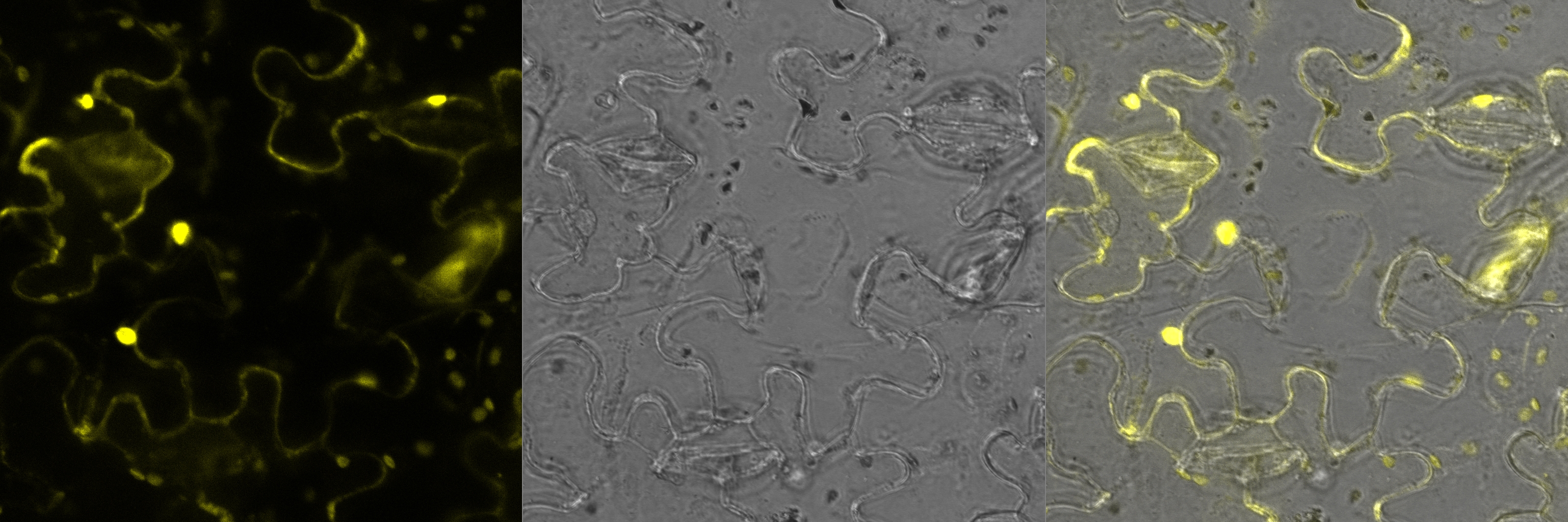

Supplement: Supplementary file 13 — Appendix Figure S8 Source Data [file 44319_2025_556_MOESM13_ESM.zip › Appendix Figure S8/S8A/SRAS1.2-cYFP+nYFP-DSK2B.jpg]

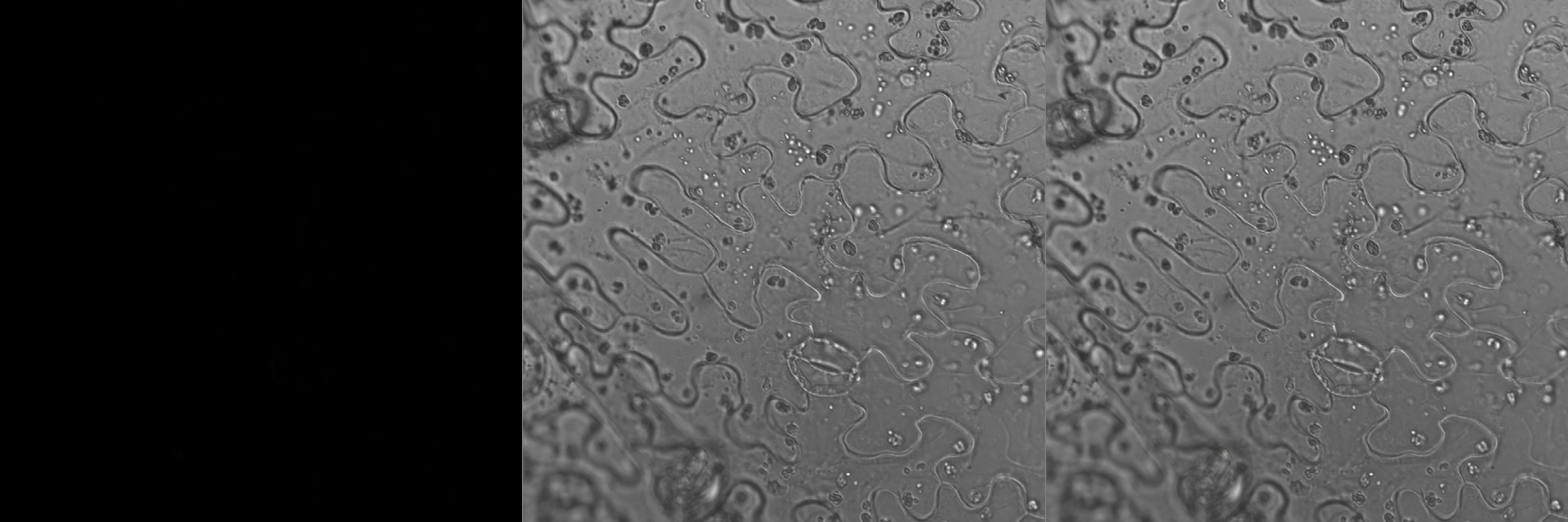

Supplement: Supplementary file 13 — Appendix Figure S8 Source Data [file 44319_2025_556_MOESM13_ESM.zip › Appendix Figure S8/S8A/SRAS1.2-cYFP+nYFP.jpg]

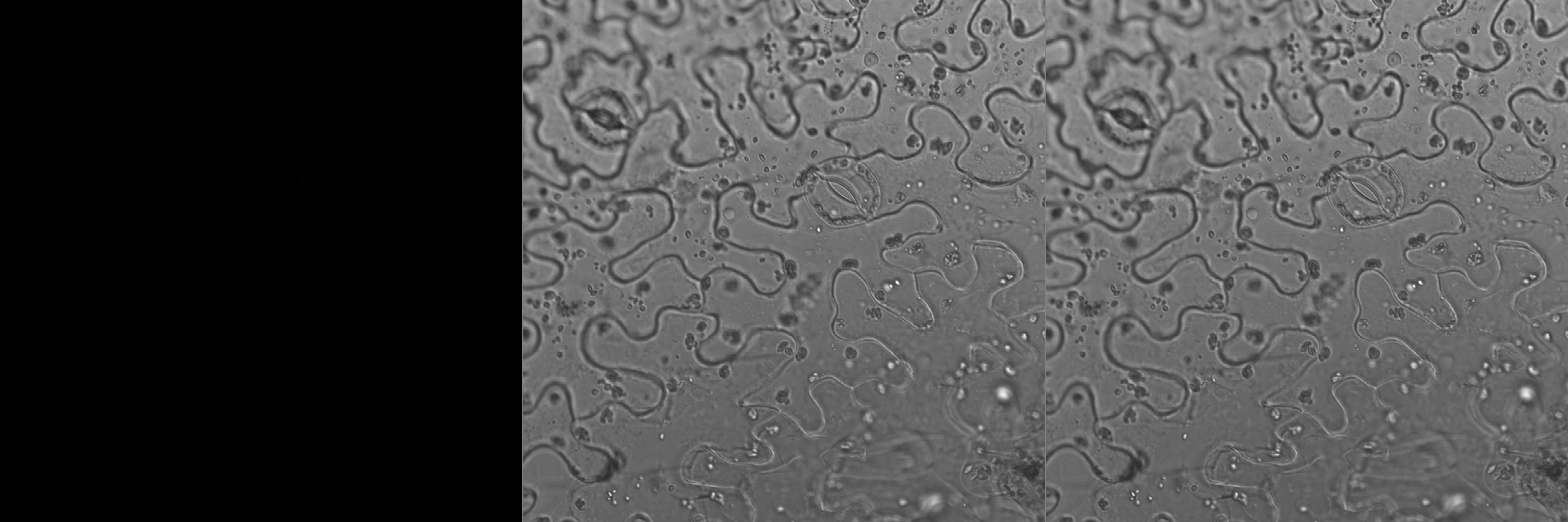

Supplement: Supplementary file 13 — Appendix Figure S8 Source Data [file 44319_2025_556_MOESM13_ESM.zip › Appendix Figure S8/S8A/cYFP+nYFP-DSK2B.jpg]
