# Supplementary material for: SRAS1.1 E3 ligase mediates DSK2A degradation to regulate autophagy and drought tolerance in Arabidopsis
Source: EMBO Rep. 2025 Aug 22;26(19):4794–819. doi: 10.1038/s44319-025-00556-9 (PMC12508185; doi:10.1038/s44319-025-00556-9)
Supplement: Supplementary file 14 — Expanded View Figures [file 44319_2025_556_MOESM14_ESM.pdf]

Expanded View Figures

Figure EV1. The effects of SRAS1.1 on *Arabidopsis* drought tolerance.

(**A–C**) Representative seedlings of wild-type, *SRAS1.1-14*, *sras1.1* mutants and *proSRAS1.1:SRAS1.1/sras1.1* complementation line (*COM1*) after 14 days of growth in 1/2 MS medium with or without 250 mM mannitol. Scale bars = 1 cm. Primary root length (**B**) and lateral root density (**C**) of seedlings shown in (**A**). Values represent means ± SD ($n = 3$ biological replicates), with 20 plants analyzed per replicate. (**B**) *P* values < 0.0001 (wild-type vs *SRAS1.1-14*), <0.0001 (wild-type vs *sras1.1*), 0.0217 (wild-type vs *COM1*), <0.0001 (*SRAS1.1-14* vs *sras1.1*), <0.0001 (*SRAS1.1-14* vs *COM1*) < 0.0001 (*sras1.1* vs *COM1*). (The following is the same order). (**C**) *P* values < 0.0001, < 0.0001, 0.8803, <0.0001, < 0.0001, 0.0013. (**D–I**) Phenotypic analysis of wild-type, *SRAS1.1-OE*, *sras1.1* mutants, and *COM1* seedlings grown on 1/2 MS medium with or without 250 mM mannitol (**D**, **G**). Images were taken 7 days after germination. Comparison of germination rates under normal conditions (**E**, **H**) and 250 mM mannitol treatment (**F**, **I**) between wild-type and transgenic plants. Values shown are means ± SD ($n = 3$ biological replicates). Significance was determined using Student's *t* test. (**E**) T = 24 h: *P* values > 0.9999 (wild-type vs *SRAS1.1-14*), >0.9999 (wild-type vs *SRAS1.1-26*), 0.9984 (wild-type vs *sras1.1*). (The following is the same order). T = 48 h: *P* values = 0.9999, 0.1836, >0.9999. T = 72 h: *P* values > 0.9999, >0.999, >0.9999. (**F**) T = 24 h: *P* values > 0.9999, >0.9999, >0.9999. T = 48 h: *P* values > 0.9999, >0.9999, >0.9999. T = 72 h: *P* values = 0.0092, 0.6742, 0.0476. T = 96 h: *P* values = 0.0067, 0.0083, 0.0347. T = 120 h: *P* values = 0.0044, 0.0057, 0.0433. T = 144 h: *P* values < 0.0001, <0.0001, 0.0191. T = 168 h: *P* values < 0.0001, <0.0001, 0.0207. (**H**) T = 24 h: *P* values = 0.6449 (wild-type vs *SRAS1.1-14*), 0.7372 (wild-type vs *sras1.1*), 0.0927 (wild-type vs *COM1*). (The following is the same order). T = 48 h: *P* values > 0.9999, 0.8942, 0.3219. T = 72 h: *P* values > 0.9999, >0.9999, >0.9999. (**I**) T = 24 h: *P* values > 0.9999, >0.9999, >0.9999. T = 48 h: *P* values > 0.9999, >0.9999, >0.9999. T = 72 h: *P* values = 0.0039, 0.0652, >0.9999. T = 96 h: *P* values = 0.0058, 0.0476, 0.7557. T = 120 h: *P* values = 0.0012, 0.0074, 0.9801. T = 144 h: *P* values = 0.0007, 0.0386, 0.9817. T = 168 h: *P* values < 0.0001, 0.0217, >0.9999. (**J–M**) Quantitative expression analysis of *DREB2A* (**J**), *RD20* (**K**), *RD29A* (**L**), and *RD26* (**M**). The data were normalized against *UBQ10* expression. Values shown are means ± SD ($n = 3$ biological replicates). (**J**) *P* values = 0.0212 (wild-type vs *SRAS1.1-14*), 0.0473 (wild-type vs *SRAS1.1-26*), <0.0001 (wild-type vs *sras1.1*), 0.9217 (*SRAS1.1-14* vs *SRAS1.1-26*) < 0.0001 (*SRAS1.1-14* vs *sras1.1*), <0.0001 (*SRAS1.1-26* vs *sras1.1*). (The following is the same order). (**K**) *P* values < 0.0001, <0.0001, <0.0001, 0.4539, <0.0001, <0.0001. (**L**) *P* values < 0.0001, <0.0001, <0.0001, 0.127, <0.0001, <0.0001. (**M**) *P* values = 0.0024, 0.0016, <0.0001, >0.9999, <0.0001, <0.0001.
Data information: For (**B**, **C**, **J–M**) different lowercase letters represent significant differences, as determined by one-way ANOVA in combination with Tukey's multiple comparisons test ($P < 0.05$). For (**E**, **F**, **H**, **I**) significance was determined using Student's *t* test.

 © The Author(s)

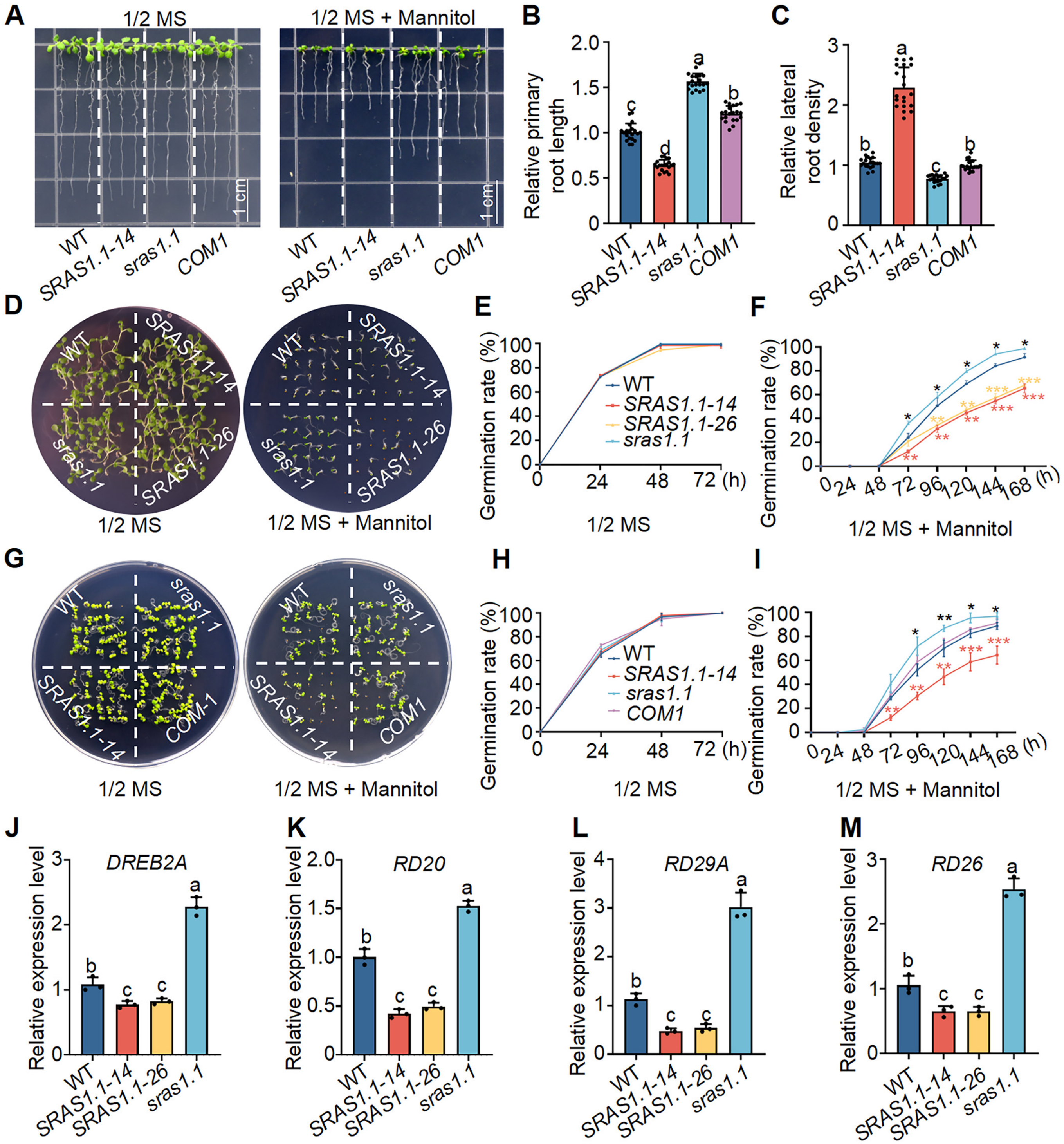
A
1/2 MS
1/2 MS + Mannitol
1 cm
WT
SRAS1.1-14
sras1.1
COM1
B
Relative primary root length
C
Relative lateral root density
D
WT
SRAS1.1-14
sras1.1
SRAS1.1-26
1/2 MS
1/2 MS + Mannitol
E
Germination rate (%)
WT
SRAS1.1-14
SRAS1.1-26
sras1.1
1/2 MS
F
1/2 MS + Mannitol
G
WT
sras1.1
SRAS1.1-14
COM-1
1/2 MS
1/2 MS + Mannitol
H
Germination rate (%)
WT
SRAS1.1-14
sras1.1
COM1
1/2 MS
I
1/2 MS + Mannitol
J
DREB2A
Relative expression level
K
RD20
L
RD29A
M
RD26
WT
SRAS1.1-14
SRAS1.1-26
sras1.1

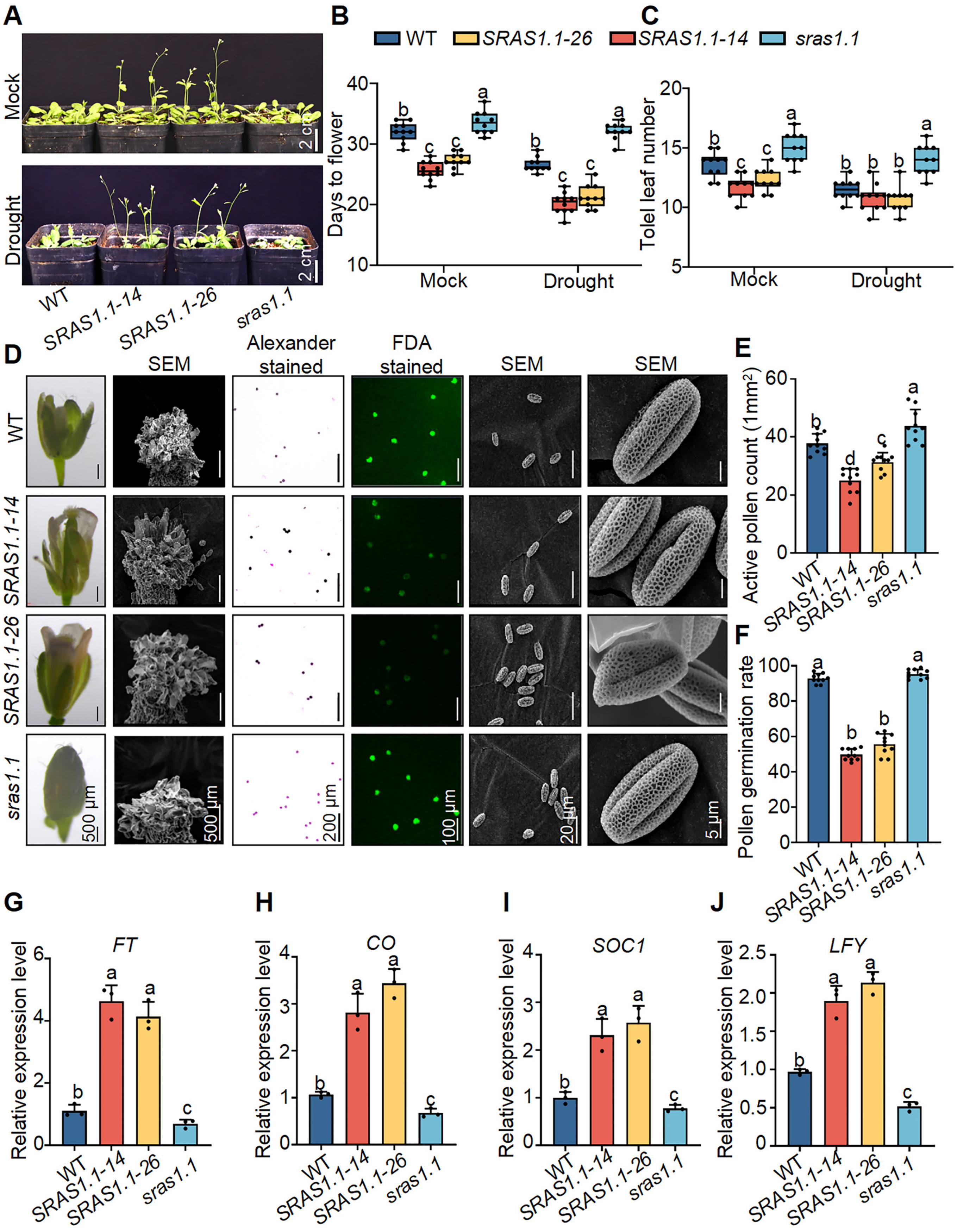
A
B
C
WT
SRAS1.1-26
SRAS1.1-14
sras1.1
Mock
Drought
2 cm
WT
SRAS1.1-14
SRAS1.1-26
sras1.1
Days to flower
Tolel leaf number
D
SEM
Alexander stained
FDA stained
SEM
SEM
500 μm
500 μm
200 μm
100 μm
20 μm
5 μm
E
Active pollen count (1mm²)
F
Pollen germination rate
G
FT
H
CO
I
SOC1
J
LFY
Relative expression level

 © The Author(s)

Figure EV2. Overexpression of *SRAS1.1* promotes flowering in *Arabidopsis*.

(**A**) Morphology of wild-type and *SRAS1.1-14*, *SRAS1.1-26*, and *sras1.1* mutants at the flowering stage under control and drought conditions. Scale bars = 2 cm. (**B**, **C**) Total leaf number at flowering (**B**) and days to flowering (**C**) in wild-type, *SRAS1.1-14*, *SRAS1.1-26*, and *sras1.1* mutants grown with and without drought treatment. Data in (**B**, **C**) are plotted with box–whisker plots: the whiskers represent maximum and minimum values, and boxes represent the upper quartile, median, and lower quartile, dots represent data points. (**B**) Mock: P values < 0.0001 (wild-type vs *SRAS1.1-14*), <0.0001 (wild-type vs *SRAS1.1-26*), 0.0021 (wild-type vs *sras1.1*), 0.0917 (*SRAS1.1-14* vs *SRAS1.1-26*), <0.0001 (*SRAS1.1-14* vs *sras1.1*), <0.0001 (*SRAS1.1-26* vs *sras1.1*). (The following is the same order). Drought: P values < 0.0001, <0.0001, <0.0001, 0.4112, <0.0001, <0.0001. (**C**) Mock: P values = 0.0016, 0.0452, 0.0274, 0.5916, <0.0001, <0.0001. Drought: P values = 0.4801, 0.3633, <0.0001, 0.9968, <0.0001, < 0.0001. (**D**) Analysis of pollen grains from wild-type, *SRAS1.1-14*, *SRAS1.1-26*, and *sras1.1* mutants using alexander staining, fluorescein diacetate (FDA) staining, and scanning electron microscopy (SEM), respectively. (**E**, **F**) Number of FDA-stained pollen grains per 1 mm^2 (**E**), in vitro pollen germination rates (**F**) of wild-type, *SRAS1.1-14*, *SRAS1.1-26*, and *sras1.1* mutants. Values represent means ± SD ($n = 3$ biological replicates), with 10 plants analyzed per replicate. (**E**) P values < 0.0001 (wild-type vs *SRAS1.1-14*), 0.0104 (wild-type vs *SRAS1.1-26*), 0.0037 (wild-type vs *sras1.1*), 0.0297 (*SRAS1.1-14* vs *SRAS1.1-26*), <0.0001 (*SRAS1.1-14* vs *sras1.1*), <0.0001 (*SRAS1.1-26* vs *sras1.1*). (The following is the same order). (**F**) P values < 0.0001, <0.0001, 0.4716, 0.0576, <0.0001, <0.0001. (**G–J**) Quantitative expression analysis of *FT* (**G**), *CO* (**H**), *SOC1* (**I**), and *LFY* (**J**). The data were normalized against *UBQ10* expression. Values shown are means ± SD ($n = 3$ biological replicates). (**G**) P values < 0.0001 (wild-type vs *SRAS1.1-14*), <0.0001 (wild-type vs *SRAS1.1-26*), 0.0055 (wild-type vs *sras1.1*), 0.4145 (*SRAS1.1-14* vs *SRAS1.1-26*), <0.0001 (*SRAS1.1-14* vs *sras1.1*), <0.0001 (*SRAS1.1-26* vs *sras1.1*). (The following is the same order). (**H**) P values = 0.0002, <0.0001, 0.0313, 0.0711, <0.0001, <0.0001. (**I**) P values = 0.0011, 0.0003, 0.0389, 0.616, 0.0004, 0.0001. (**J**) P values < 0.0001, <0.0001, 0.0099, 0.1706, <0.0001, <0.0001. Data information: For (**B**, **C**, **E**, **F**, **G–J**) different lowercase letters represent significant differences, as determined by one-way ANOVA in combination with Tukey's multiple comparisons test ($P < 0.05$).

© The Author(s)

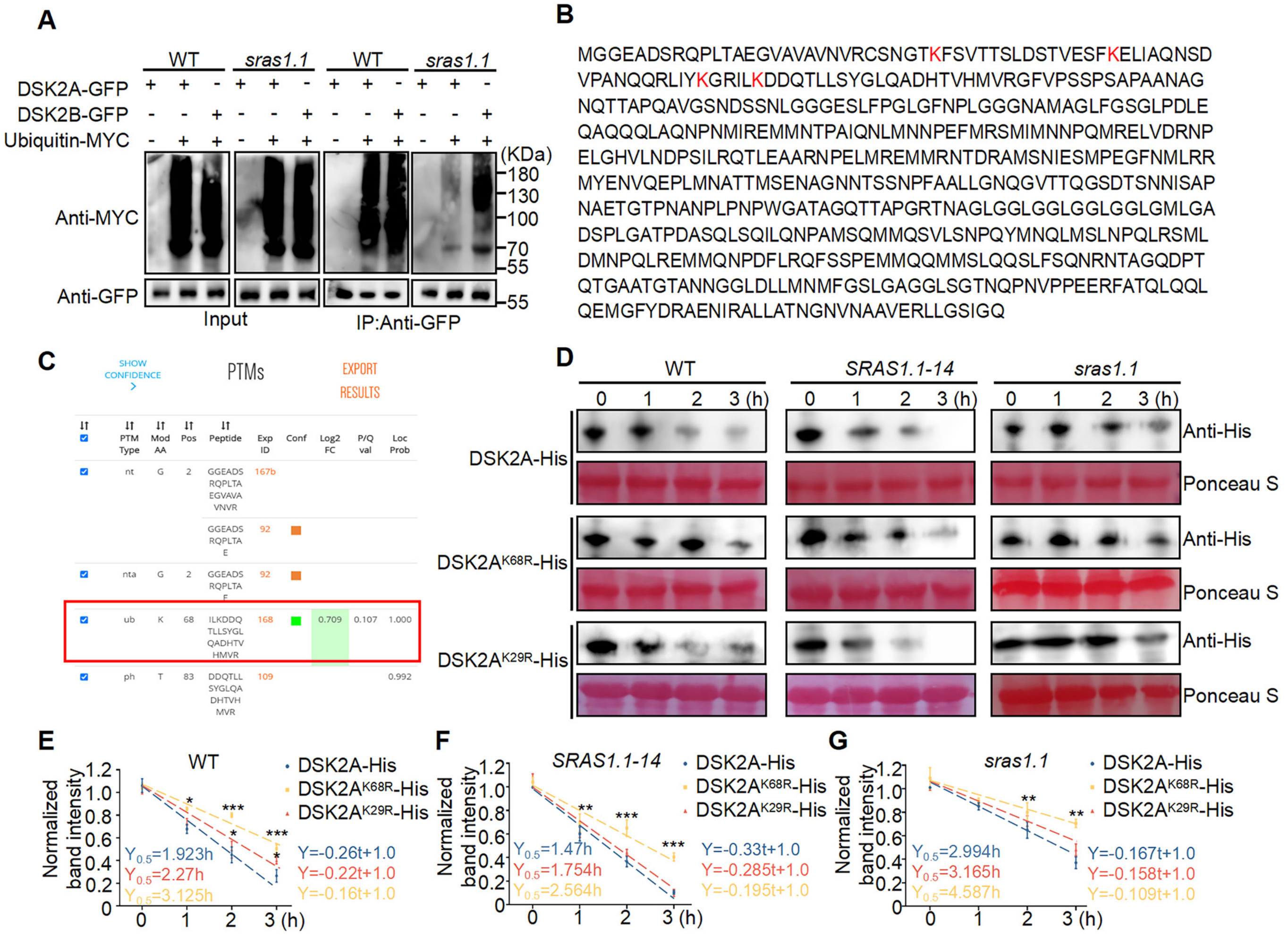

Figure EV3. DSK2A is ubiquitinated by SRAS1.1, and Lys68 is the major ubiquitination site.

(**A**) Ubiquitination of DSK2A-GFP and DSK2B-GFP in wild-type and *sras1.1* plants, detected in an *Arabidopsis* protoplast transient transformation assay. Anti-GFP antibody was used to immunoprecipitate DSK2A-GFP and DSK2B-GFP, and anti-MYC antibody was used to detect Ubiquitin-MYC. (**B**) Amino acid sequence of DSK2A with lysine residues highlighted in red. (**C**) Predicted post-translational modifications of DSK2A, with Lys68 indicated as the major ubiquitination site. (**D**) Degradation rates of DSK2A-His, DSK2AK^{68}R-His, and DSK2AK^{29}R-His in cell-free degradation assays using protein extracts from wild-type, *SRAS1.1-14*, and *sras1.1* mutant plants. Proteins were detected by immunoblotting with an anti-His antibody. Ponceau S staining was used as a loading control. (**E–G**) Quantified degradation rates of DSK2A-His (E), DSK2A^{K68R}-His (**F**), and DSK2A^{K29R}-His (**G**) plotted as linear regression curves. $Y_{0.5}$ denotes the time required for 50% degradation. Values shown are means ± SD ($n = 3$ biological replicates). All comparisons were made against DSK2A-His and analyzed by linear regression and Student's t test (*$P < 0.05$; **$P < 0.01$; ***$P < 0.001$). (**E**) T = 1 h: P values = 0.0273 (DSK2A-His vs DSK2A^{K68R}-His), 0.3123 (DSK2A-His vs DSK2A^{K29R}-His). (The following is the same order). T = 2 h: P values = 0.0009, 0.0433. T = 3 h: P values = 0.0007, 0.0322. (**F**) T = 1 h: P values = 0.0072, 0.4917. T = 2 h: P values = 0.0005, 0.8971. T = 3 h: P values < 0.0001, 0.3707. (**G**) T = 1 h: P values = 0.2442, 0.9017. T = 2 h: P values = 0.0074, 0.0981. T = 3 h: P values = 0.0095, 0.0672.

 © The Author(s)

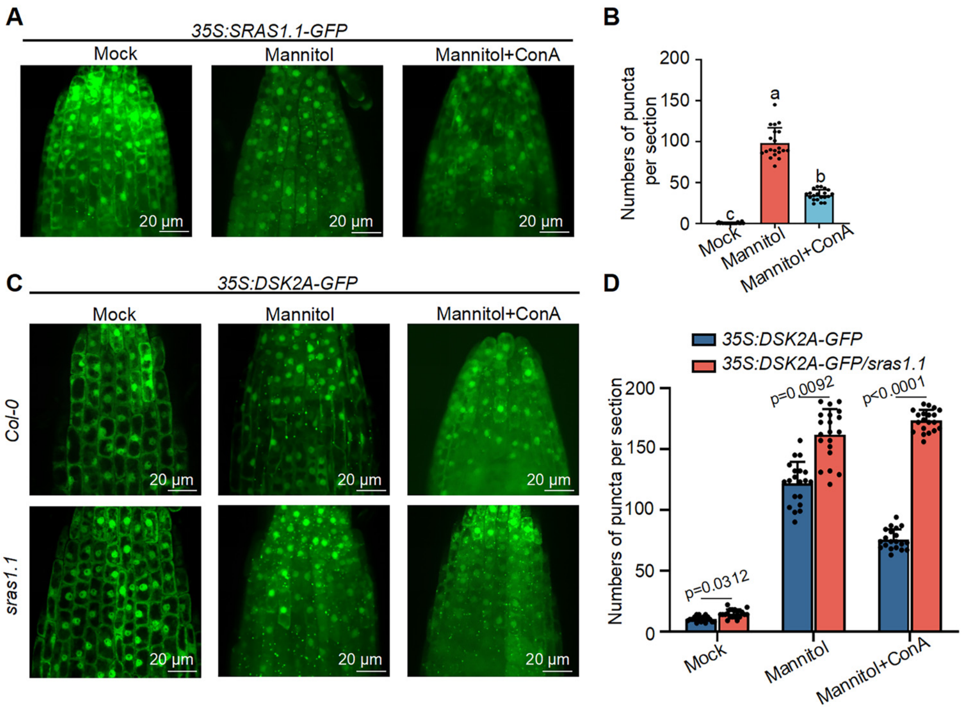

Figure EV4. SRAS1.1 is involved in the regulation of cellular autophagy.

(**A**) Confocal analysis of *35S:SRAS1.1-GFP* transgenic *Arabidopsis*. 5-day-old seedlings were exposed to 250 mM mannitol liquid medium for 30 min and then visualized by confocal laser scanning microscopy. Scale bars = 20 µm. (**B**) Numbers of puncta per section in the root cells of the *35S:SRAS1.1-GFP* transgenic *Arabidopsis* in (**A**). Values shown are means ± SD ($n = 3$ biological replicates), with 20 plants analyzed per replicate. *P* values < 0.0001 (Mock vs Mannitol), <0.0001 (Mock vs Mannitol + ConA), <0.0001 (Mannitol vs Mannitol + ConA). (**C**) Confocal analysis of *35S:DSK2A-GFP* and *35S:DSK2A-GFP/sras1.1* transgenic plants. Five-day-old seedlings were exposed to 250 mM mannitol liquid medium for 30 min and then visualized by confocal laser scanning microscopy. Scale bars = 20 µm. (**D**) Numbers of puncta per section in the root cells of the transgenic plants in (**C**). Values shown are means ± SD ($n = 3$ biological replicates), with 20 plants analyzed per replicate. Significance was determined using Student's *t* test. Mock: *P* values = 0.0312 (*35S:DSK2A-GFP* vs *35S:DSK2A-GFP/sras1.1*). Mannitol: *P* values = 0.0092. Mannitol + ConA: *P* values < 0.0001. Data information: For (**B**) different lowercase letters represent significant differences, as determined by one-way ANOVA in combination with Tukey's multiple comparisons test ($P < 0.05$). For (**D**) significance was determined using Student's *t* test.

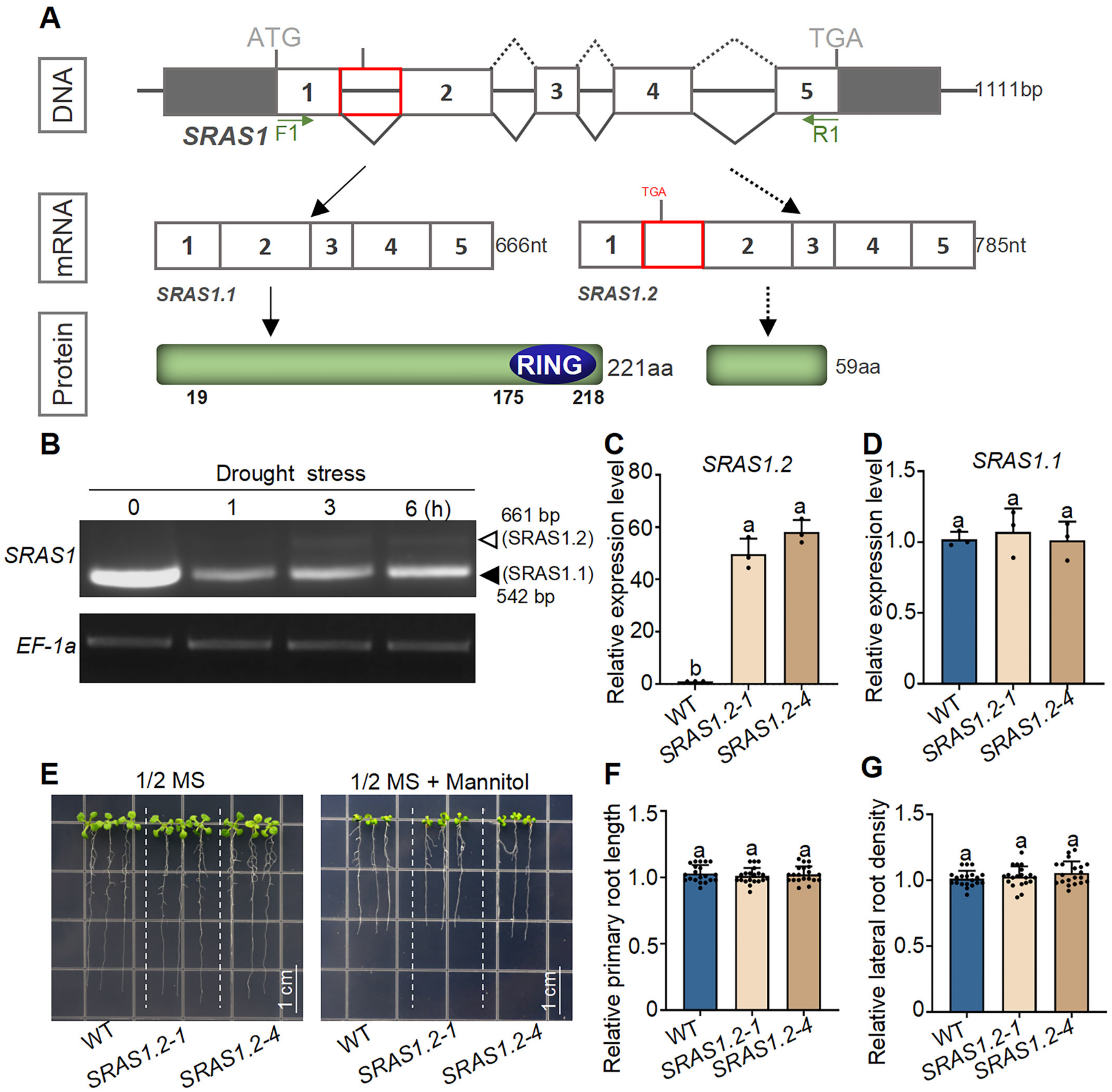

Figure EV5. Alternative splicing of *SRAS1* in response to drought stress.

(**A**) Schematic diagram of *SRAS1* gene. Two different intron splice sites are indicated in the gene diagram. Arrows (F1 and R1) indicate the location of primers used in RT-PCR and green color represents the exon region; blue region represents the RING domain. (**B**) RT-PCR analysis of the expression levels of *SRAS1.1* and *SRAS1.2* at 0, 1, 3, and 6 h after 250 mM mannitol treatment. Elongation factor 1α (*EF-1α*) was used as an internal control. (**C**, **D**) Quantitative measurement of the expression levels of *SRAS1.2* (**C**) and *SRAS1.1* (**D**) in wild-type, *SRAS1.2* overexpressing plants (*SRAS1.2-1*, *SRAS1.2-4*). *UBQ10* was used as an internal control. Values shown are means ± SD ($n = 3$ biological replicates). (**C**) *P* values < 0.0001 (wild-type vs *SRAS1.2-1*), <0.0001 (wild-type vs *SRAS1.2-4*), 0.1163 (*SRAS1.2-1* vs *SRAS1.2-4*). (The following is the same order). (**D**) *P* values = 0.865, 0.9977, 0.8333. (**E–G**) Representative seedlings of wild-type, *SRAS1.2-1*, and *SRAS1.2-4* after 10 days of growth in 1/2 MS medium with or without 250 mM mannitol. Scale bars = 1 cm. Primary root length (**F**) and lateral root density (**G**) of seedlings shown in (**E**). Values shown are means ± SD ($n = 3$ biological replicates), with 20 plants analyzed per replicate. (**F**) *P* values = 0.6465 (wild-type vs *SRAS1.2-1*), 0.9411 (wild-type vs *SRAS1.2-4*), 0.8409 (*SRAS1.2-1* vs *SRAS1.2-4*). (The following is the same order). (**G**) *P* values = 0.7984, 0.1795, 0.4835. Data information: For (**C**, **D**, **F**, **G**) different lowercase letters represent significant differences, as determined by one-way ANOVA in combination with Tukey's multiple comparisons test ($P < 0.05$).

 © The Author(s)
